# Supramolecular Frameworks from Graphene Edge-Grafted with Ni-Salphen Complexes and Pd-PTA Linkers

**DOI:** 10.1021/acsomega.5c04731

**Published:** 2025-08-09

**Authors:** Paulina Hernández-Pacheco, Gustavo A. Zelada-Guillén, Martha V. Escárcega-Bobadilla

**Affiliations:** Department of Organic Chemistry, School of Chemistry, 7180 National Autonomous University of Mexico (UNAM). Circuito Escolar s/n, Ciudad Universitaria, Mexico City 04510, Mexico

## Abstract

Edge oxidized graphene oxide (EOGO) is a promising graphene variant that has remained broadly unexplored as a two-dimensional (2D) supramolecular building block. This work aimed to construct graphene supramolecular frameworks (GSFs) using EOGO building blocks and [PdCl_2_(3,5,7-triaza-7-phosphaadamantane)_2_] (Pd-PTA) as ditopic guest linkers through noncovalent reticulation. Pd-PTA facilitated host–guest–host interconnection of the graphene units selectively through supramolecular hinges based on a Ni-Salphen scaffold hydroxylated at variable positions but formerly grafted to the carboxyl moieties exclusively present at the EOGO edges to maximize directional efficiency in reticulation. Synthesis and characterization of the supramolecular complexes formed between the Ni-Salphen variants and Pd-PTA, together with spectrophotometric titrations in solution, have confirmed concurrence of 1:1 and 2:1 stoichiometries for the [Ni-Salphen]/[Pd-PTA] systems. In contrast, EOGO-*g*-[Ni-Salphen] hybrid counterparts developed stoichiometry 1:1 for [Ni]/[Pd-PTA] at up to 1000-fold higher *K*_11_ and depicted the emergence of successive host–guest–host concatenation at [Ni] via 1:2 species formed through similar strength (*K*_11_∼*K*_12_). This stoichiometric inversion apparently enabled a cooperative oligomerization, as was further suggested by Hill plots and a concentration-dependent rise in fluorescence that progressed asymptotically upon the addition of Pd-PTA. Kinetically controlled assembly and solvent evaporation facilitated long-range arrangements, which we confirmed qualitatively and quantitatively by solid-state evaluations, thus offering a next generation of supramolecular frameworks.

## Graphical abstract

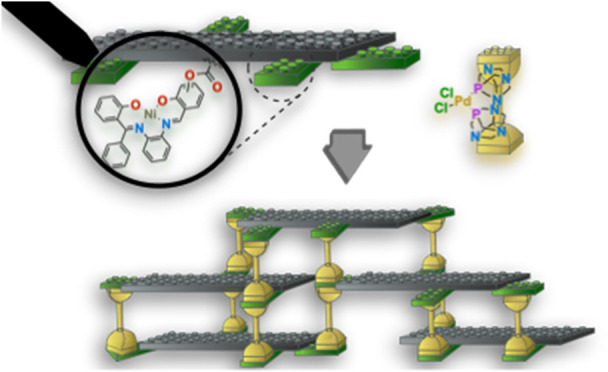

## Introduction

Spatially specific derivatization of one-dimensional (1D) and 2D nanostructured materials is a challenging task that could open the door to a next generation of anisotropically tailored properties at the nanoscale.^1–3^ For instance, it could enable matter relocation controlled through the induction of orientationally selective noncovalent interactions, thus triggering autonomous organization of nanostructures and, eventually, revealing new properties at the microscale.^4–6^ Unfortunately, the activation of chemical anisotropy through highly selective supramolecular processes upscaled for long-range self-organization as in earlier reports that have used discrete molecules^7^ and 1D carbonaceous allotropes such as carbon nanotubes^8–11^ has remained unexploited in 2D counterparts such as graphene due to technological barriers.

Edge oxidized graphene oxide (EOGO) is a unique carbonaceous nanomaterial that is produced by the selective oxidation of graphene sheets to exclusively introduce coplanar carboxylic acid groups in the periphery.^12^ As a result, the internal preservation of an sp^2^ network at each sheet maintains graphene’s original properties, such as electron mobility, photophysical sensitivity, and thermal conductivity, whereas new amphiphilic interfacial characteristics are incorporated at the same time that subsequently yield individually exfoliated sheets. Furthermore, the resultant anisotropic localization of carboxylic acid moieties enables spatial selectivity in, for example, nucleophilic acyl substitution reactions (S_N_Ac), which could then proceed only at the rim of each sheet. This exceptional situation unlocks covalent functionalization in a center-edge disparity fashion, thus endowing synthetic control in the fine-tuning of novel nanomaterials. Unfortunately, there are scarce reports in the literature on material design that make use of EOGO, which is used to date only as a valuable additive or a filler,^12–15^ that respectively improved either redox^16^ or mechanical^17^ properties of matrices such as cement, among others. However, our group has reported that EOGO can also be useful in the design of multifunctional host–guest (H/G) systems with potential applications in nonlinear optics, through the extension of their chromophoric traits under supramolecular stimuli.^18^ We showed that covalent functionalization of EOGO with a metal-Salphen complex could occur without limiting the host-type binding capabilities of the incorporated Lewis acidic metal centers toward diversely sized or sterically constrained monodentate N-donor pyridinic guests. In contrast, the coexistence of large π-conjugated domains at the core of the graphene component together with the active binding sites grafted at the borders rendered tunability of the native host–guest association capabilities of the complex, both in terms of stoichiometry and in strength of the same toward dynamic and more efficient regimes, respectively. This also opened future opportunities for addressing self-assembly of EOGO units, depending on the size, shape, or topicity of external supramolecular guests. However, thus far, it remained unclear whether an H/G supramolecular strategy could be indeed effective in building multimeric architectures through a combination of noncovalent reticulation guest components and graphene sheets grafted with hosts at the borders. Therefore, the objective of this study was to evaluate whether the preparation of graphene supramolecular frameworks (GSF) was possible by the supramolecular reticulation of host metal-Salphen complexes covalently tethered to the edges of EOGO (2D-) building blocks utilizing a suitable N-donor ditopic guest linker.

## Results and Discussion

### Design of Framework Components

In the current work, we capitalized upon the squared planar geometry present at the N_2_O_2_ tetradentate cavity around the metal center of a Ni-Salphen scaffold, to trigger a supramolecular frameworking (reticulation) of their covalent hybrids obtained from EOGO, namely, EOGO-*g*-[Ni-Salphen]. We carried this out looking forward to gain two free axial sites per metal-Salphen unit left identical for further bilateral H/G interactions in the form of a bidirectional noncovalent hinge. In this way, both planar sides of each hinge could offer opposite binding spots of noncompeting equivalent guest association strength capabilities (*K*_11_∼*K*_12_), each with wide angular permissiveness and low steric constraint and thus, larger conformational binding flexibility for an easier orthogonal access in noncovalent reticulation among the graphene planes.^18–20^ The selected scaffold also forms a π-conjugated system which yields photoresponsiveness,^21–23^ and offers synthetic flexibility in its design and functionalization.^24–27^ Therefore, we systematically explored the influence of a free hydroxyl group positioned ortho- (**1**), meta- (**2**), and para- (**3**) relative to the phenolate moiety in the N_2_O_2_ cavity within the salicylic backbone of the Salphen ligand component (Chart 1, Scheme S1, Figures S1–S9). In this form, we aimed to have the possibility to modulate the grafting orientation of the scaffold relative to the carboxylic groups on EOGO and, consequently, to manipulate the torsional freedom of the C–O–C bridge in the ester moieties that ultimately regulates flexibility in the dihedral angle occurring between the N_2_O_2_ plane and the carbonaceous plane (Chart 2). With this in mind, we wanted to gain access to different pointing directions for the hinges that in turn addressed guest alignment and maximized their mutual accessibility during reticulation, while a chromophoric efficiency was achieved, looking forward to a spectrophotometric follow-up through titrations and fluorimetry.

**Chart 1:**
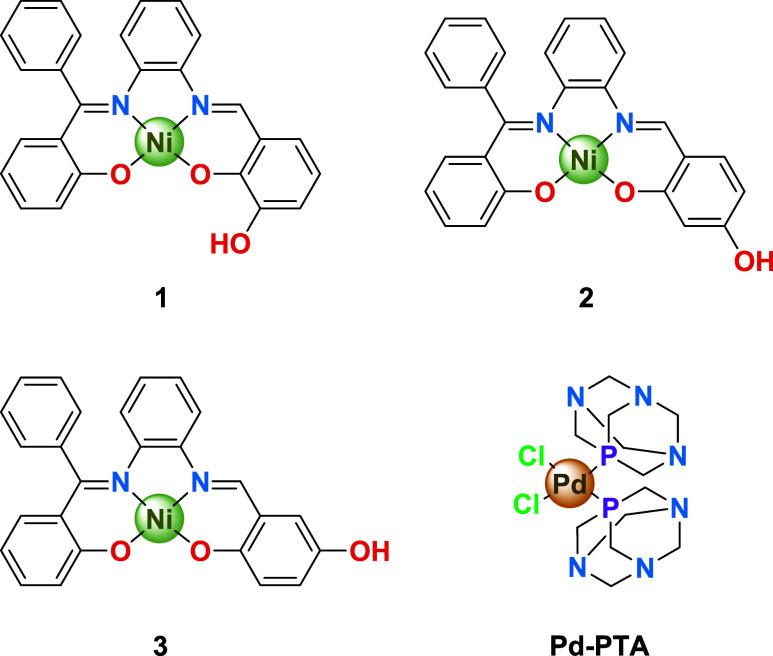
Ni-Salphen Scaffold Variants **1**-**3** and Pd-PTA Complex Synthesized

**Chart 2:**
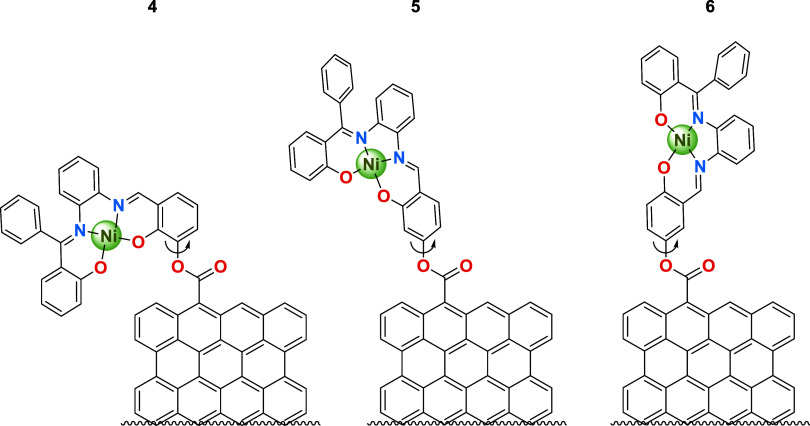
Different Rotational Flexibilities Expected for the Grafting Orientations at the Ni-Salphen Components in Their Respective EOGO-*g*-[Ni-Salphen] Hybrid Nanomaterials **4**–**6**

We evaluated the three grafting orientations of the Ni-Salphen complexes **1**, **2**, and **3** in their respective EOGO-*g*-[Ni-Salphen] product variants **4**, **5**, and **6** (Chart 2, Scheme S2, Figures S10–S21), to determine their effect on H/G stoichiometry (H_n_G_m_) and association strength (*K*_nm_), ideally, against ditopic N-type Lewis bases that offered simultaneously reticulation and stratification possibilities either in solution or in solid state. Nevertheless, given the potentially irregular insertions expected for Ni-Salphen units over each sheet of EOGO due to either incomplete grafting or imperfect carboxyl group frequencies at the edges of EOGO in the raw material, we had to follow compensatory strategies. On one side, we optimized grafting efficiency toward the saturation regime during synthesis by depletion of reactants and validation by spectrophotometric acid–base titrations. However, on the other side, we needed to maximize host–host reticulation success at the large scale by interconnecting guests that met ditopic and rigid interlinking properties, but at the same time, they could offer the necessary degree of freedom for directional interactions. The latter in sum denoted an empirical challenge, since, at a molecular level, it would represent to have designed a two-driveshaft differential system with independent rotation in their drive axles and articulation freedom, both possessing a terminal pinion as well that could bind simultaneously to two moveable hinges in a dynamic, reversible, and multiangular manner. For this purpose, we synthesized [PdCl_2_(3,5,7-triaza-7-phosphaadamantane)_2_] complex, Pd-PTA (Chart 1), as a suitable candidate in terms of topicity, size, rotatory articulation capabilities, and binding orientation opportunities. In this regard, the multiple mutually equivalent neighboring Lewis basic N atoms at the 3,5,7-triaza-7-phosphaadamantane ligand (PTA) have shown multisite monodentate interactions with Lewis acids such as certain coordinated hard metals, whereas the single P atom typically proceeds monodirectionally against softer metal counterparts.^28^ This bifunctional attribute offers vectorial binding selectivity and multimodal monodentate association opportunities at the same time, which can be reinforced by the incorporation of the Pd center through the P atom, thus creating rotational freedom in each ligand moiety through their respective Pd–P bonds, while up to 3^2^ chemically equivalent noncovalent binding form combinations are kept by the mutually identical 2 × 3 N atoms across the PTA domains. Furthermore, such phosphine could offer additional advantages such as water solubility and oxidation resistance when compared to similarly sized phosphines (e.g., trimethylphosphine).^29^ These advantages have facilitated earlier applications for PTA derivatives in homogeneous and biphasic catalysis,^30–32^ as well as in supramolecular chemistry, where the differential hardness of the P and N Lewis bases within PTA has been useful in otherwise, conformationally challenging, long distance arrangements such as in coordination polymers,^33–35^ metal–organic frameworks (MOFs),^36,37^ and luminescent supramolecular aggregates.^38^

### Preparation of EOGO-*g*-[Ni-Salphen] Grafted Materials

We reached our target of an optimal grafting efficiency in pristine EOGO by considering that their oxygen content was in the form of purely carboxylic acid moieties (0.15 mol of COOH per 100 g of EOGO) and used this to optimize the molar ratio of reactants. This allowed for esterification reaction yields of 93, 95, and 98%, respectively, for **4**, **5**, and **6**, and graft contents of 37.3, 38.2, and 39.6% wt., according to thermogravimetric analysis, TGA (Figures S12, S16, and S20), which indicate almost full grafting in all cases. This in turn facilitated stoichiometric control in H/G evaluations as discussed later, and enabled seamless cross-validations of these grafting efficiencies by spectrophotometric titrations as reported earlier.^18^ In our assessments, a careful stepwise Fourier transform infrared spectroscopy (FT-IR) follow-up of each modification confirmed that free carboxylic acids remained at least undetectable and should not participate in supramolecular equilibria.^18,28^ In this regard, an offset of FT-IR spectra from pristine EOGO, Ni-Salphen varieties **1**-**3**, and their grafted counterparts **4**-**6** (Figures S32, S37, and S42) shows the occurrence of ester C=O st. band at 1720–1724 cm^–1^ together with phenolic ester C–O st. bands at 1220–1240 cm^–1^ for grafted nanomaterials **4**-**6**, while not exhibiting other carbonyl bands attributable to any remaining carboxylic acid moieties from EOGO, such as C=O st. at 1714 cm^–1^. Furthermore, the esterified materials **4**-**6** exhibited no evidence of the strong doublet aromatic C–OH st. characteristic of the phenolic hydroxyls, observed in compounds **1**-**3** at 1219–1250, 1213–1242, and 1200–1244 cm^–1^, respectively. Additionally, **4**-**6** show the presence of both C=N st. bands from the ketimine and aldimine moieties (1530–1650 cm^–1^) from the grafted Ni-Salphen complex. The exhibition of a trend in **4**-**6**, mirroring the profile of molecular components **1**-**3** and the emergence of ester bands in EOGO postgrafting, alongside the absence of free carboxylic acids and free phenolic hydroxyl bands, strongly suggests a successful esterification process without adsorbed reactants. Broadening of the band observed around 3400 cm^–1^ for the hybrid systems is, on the contrary, consistent with remaining water because of the hygroscopicity of the materials, as proven by TGA.^28^ The previous FT-IR and TGA results in combination with energy-dispersive X-ray elemental mapping for Ni, N, and O (Figures S11, S15, and S19) confirmed that compounds **1**-**3** were accordingly grafted to EOGO in **4**-**6**.

### Molecular Association of Pd-PTA and Ni-Salphen

The titrimetric assessment of the H/G association profile of the Pd-PTA complex toward structures **1**, **2**, and **3** via ultraviolet–visible (UV–vis) spectrophotometry showed that Pd-PTA complex and Ni-Salphen complexes **1**-**3** produced two [Pd-PTA]/[Ni-Salphen] stoichiometries, 1:1 and 1:2, and these coexisted at variable ratios depending on the progress of titration. In this regard, Pd-PTA thus performed as the host, and Ni-Salphen complexes **1**-**3** played the role of guests (Figure S22). The titrations rendered single equivalence points of 1.98 for **1** (confidence interval, CI, *p* < 0.05:1.90–2.08), 1.97 for **2** (CI *p* < 0.05:1.89–2.05), and 1.95 for **3** (CI *p* < 0.05:1.81–2.07), which shows that species 1:2, [Pd-PTA]/[Ni-Salphen]_2_, might be the predominant form. A more detailed assessment using TGA and differential scanning calorimetry, DSC, (Figures S23–S25) of their respectively synthesized [Pd-PTA]/[Ni-Salphen] supramolecules (Scheme S3), namely, **7** (from **1**), **8** (from **2**), and **9** (from **3**), confirmed that up to two Ni-Salphen units were able to effectively bind to each Pd-PTA, regardless of the hydroxyl substitution pattern, which indicated the preservation of the same stoichiometry either in solution or in the solid state. Titration data from solutions indicated that formation of [Pd-PTA]/[Ni-Salphen] systems took place in a stepwise manner, first yielding stoichiometry 1:1, followed then by the progressive achievement of 1:2 species, whereas the process likely occurred by the participation of one nitrogen atom per PTA unit being bound to each Ni center. Their respective trends in the binding constants *K*_nm_ (Table 1), *cf*. *K*_11_(**1**)>*K*_11_(**2**)∼*K*_11_(**3**) and *K*_12_(**2**)∼*K*_12_(**1**)≫*K*_12_(**3**), as well as the appearance of Hill coefficients larger than the unit in the respective Hill plots extracted from each UV–vis titration (Figures S44–S46) suggested that a cooperative effect in the form of intramolecular participation of hydrogen bonding to one of the adjacent nitrogen atoms from each PTA ligand is likely to occur for **1** and **2**, driven by the free hydroxyls located at the closest positions to the Ni centers. Contrarily, an antagonistic effect seems to occur during the second association when the free hydroxyl is placed farthest from the Ni as in **3**, as shown by *K*_12_ now appearing in the dissociation regime (*K*_nm_ < 1), *i.e., K*_12_(**3**)≪*K*_11_(**3**). If the latter is compared to the lack of any effect observed for **2** when transitioning from the first to the second association, *i.e., K*_12_(**2**)∼*K*_11_(**2**), the scenario depicts the probable occurrence of noncovalent parasite equilibria when a second unit of **3** tries to bind to the already formed 1:1 species, which seems to be overridden by the apparent cooperativity effect that might be already occurring at the same time (Figure S46). We potentially ascribe this hurdle to competitive intermolecular interactions likely triggered by the −OH when it is present in the most exposed form, as in **3**.^7^ Nevertheless, we speculate that an interplay of either inter- or intramolecular interactions from the free hydroxyl moieties in the Salphen’s salicylic portions to the PTA ligand parts could derive in effects that go far beyond a simple solution-restricted phenomena and could represent an entropic barrier in further assembling steps even in the solid state. The latter was evidenced by the fact that progressive thermal decomposition of **7**-**9** (Figures S23–S25) proceeded in a different manner depending on the hydroxyl substitution pattern of the Ni-Salphen components, i.e., weight losses appearing at different first derivative peak temperatures (T_p_). The overall situation supports the idea that any occurrence of parasite interactions may impede efficient frameworking and, by design, they must be overridden for those purposes.

**Table 1:** H/G Roles and Binding Constants Assessed by UV–Vis Titration for Molecular (**1-3**) and EOGO-*g*-[Ni-Salphen] (**4-6**) Systems vs. Pd-PTA

	**1** ^a^	**2** ^a^	**3** ^a^	**4** ^b^	**5** ^b^	**6** ^b^
H/G^c^	[Pd-PTA]/[Ni-Salphen]			[Ni]/[Pd-PTA]		
*K*_11_ ^d^	(7.99 ± 0.20) × 10^4^ M^–1^	(3.70 ± 0.01) × 10^3^ M^–1^	(10.53 ± 0.04) × 10^3^ M^–1^	(8.81 ± 0.89) × 10^5^ M^–1^	(3.72 ± 0.79) × 10^6^ M^–1^	(6.91 ± 0.58) × 10^5^ M^–1^
*K*_12_ ^d^	(8.69 ± 0.13) × 10^2^ M^–1^	(3.42 ± 0.02) × 10^3^ M^–1^	(5.93 ± 0.06) × 10^–6^ M^–1^	(1.42 ± 0.11) × 10^6^ M^–1^	(2.47 ± 0.06) × 10^5^ M^–1^	(9.14 ± 0.55) × 10^5^ M^–1^

a Nominal concentrations: 2.00 × 10^–5^ mol L^–1^ for H = Pd-PTA, 6.00 × 10^–4^ mol L^–1^ for G = **1**–**3**.

b Nominal concentrations: 1.50 × 10^–5^ mol L^–1^for H = [Ni] in **4**–**6** at 0.05 g mL^–1^ of these, 5.20 × 10^–5^ mol L^–1^for G = Pd-PTA.

c Titrated solutions 2.00 mL.

d Parameters and stoichiometries determined with Bindfit^39–41^ by iterative optimizations using absorbance readouts from five λ values.

### Association of EOGO-*g*-[Ni-Salphen] and Pd-PTA

These possibilities seem valid after comparing the earlier data with further H/G evaluations carried out for the [Ni] units in the Ni-Salphen hybrid counterparts **4**, **5**, and **6**, against Pd-PTA once each scaffold version was grafted to EOGO through esterification under Steglich conditions (Scheme S2). These evaluations showed that, in contrast to the molecular systems **1**-**3** in which up to two peripheric Ni-Salphen units were assembled to a central Pd-PTA, the [Ni]/[Pd-PTA] species from **4** to **6** in turn coexisted in two forms, an equimolar version 1:1 and another one with one [Ni] center equally binding to two Pd-PTA units. This suggests that [Ni] centers within grafted nanomaterials **4**-**6** played the role of host species and the Pd-PTA complex worked as the guest. The respective titration data are shown (Figure S26), indicating that, at least in solution, 1:1 species are formed at 10- to 1000-fold stronger conditions than in their nongrafted versions (Table 1). Furthermore, Ni-Salphen moieties now can bear up to two Pd-PTA units per [Ni] center, as seen when solvated sheets are titrated. These second association events remained almost equally in strength than their first associations for **4** and **6**, *i.e., K*_12_(**4**)≈*K*_11_(**4**) and *K*_12_(**6**)≈*K*_11_(**6**), while **5** evolved through the second binding event at similarly competitive strength degrees with *K*_12_ just 1 order of magnitude below *K*_11_, *i.e., K*_12_(**5**) = 10^5.4^ M^–1^ and *K*_11_(**5**) = 10^6.6^ M^–1^. Both events progressed in a concentration-dependent fashion through Le Châtelier type trends in a form that for the higher concentration of Pd-PTA, the more displaced to the product the reaction. Furthermore, a Hill plot analysis over the titration data (Figures S47–S49) also suggested that the apparent cooperativity effect already detected between the individual components Ni-Salphen **1**-**3** and Pd-PTA, might have remained unaffected when the respective hybrid versions **4**-**6** were used. The whole picture suggests that, upon esterification, we also tackled any agonistic or antagonistic parasite participation from intermolecular or intramolecular interactions that could in any case alter the binding process purely occurring between a N atom of PTA and the [Ni] centers and, thus, promoted a neat mutual access from both facial sides in the N_2_O_2_ plane that in sum, facilitated their free coupling and articulation freedom. Indeed, all the spectrophotometric titrations of **4**-**6** (Figure S26, right column) portrayed sharp drops in absorbance (concentration of their unbound [Ni] components) until stoichiometry 1:1 was reached, followed afterward by the preservation of an absorbance minima in an asymptotic fashion. In the titrations, the equivalence points represent the transitional shift among these two regions and indicate the stoichiometry of the predominant species; they resulted slightly closer to the unit for **4** and **6** than for **5**, *cf*. 1.02 for **4** (CI *p* < 0.05:0.90–1.20), 1.17 for **5** (CI *p* < 0.05:0.98–1.40) and 0.98 for **6** (CI *p* < 0.05:0.88–1.12). Such behavior, in combination with the absence of collateral chromogenic species, was evidence of titrations proceeding with no further acid–base equilibria. This, on the one hand, confirmed a lack of unreacted carboxylic acids as earlier shown by FT-IR, while on the other hand, it showed that stoichiometry 1:1 was predominantly formed with **4** and **6**. The observed trend is a typical feature not only in equimolar (1:1) bicomponent systems but also in alternate copolymeric arrangements, i.e., the [-A-B-]*_n_* type that, in practical terms, are the monodimensional version of reticulation phenomena. Therefore, we believe that reticulation must proceed more efficiently when the ratio [Ni]/[Pd-PTA] approaches 1:1, even from diluted conditions as in titrations, and that once this equivalence is reached, progressive phase separation should be likely to occur by the larger size of freshly assembled oligomers. The latter was evidenced by direct observation of the quartz cells after titration, which exhibited a silver pale tonality that was removable only by mechanical means.

The emission profiles shown in fresh solutions from **4** to **6** at the [Ni]/[Pd-PTA] ratios 1:1 and 1:2 (Figure 1) evidenced as well an enhancement of intensity of up to 200% (**6** at λ_em_ = 548 nm), which was produced at a greater extent for the materials grafted with **3** and at a lower level for the counterparts from **1**, giving an order of relative increase in %ΔI_λem_ as follows: **6 > 5 > 4**. However, a kinetic follow-up of emission at 548 nm after additions of 1 or 2 equiv of Pd-PTA to [Ni] in **4**-**6** showed that these intensity enhancements proceeded progressively upward for all the systems in a nonlinear manner (Figure S27). This emission growth progressed asymptotically without reaching the final plateau for at least ca. 3 h for the three structures. The phenomenon occurred in an apparently sigmoidal fashion for **4** with a lag phase that depended on the Pd-PTA ratio used, **6** offered a more moderate behavior than the earlier example, while **5** showed the least remarked impact among the others. The overall scenario suggests that the increase of emission might be correlated with a progressive association and simultaneous substrate depletion occurring in the quartz cell when aggregation phenomena proceed between Pd-PTA and the grafted [Ni] units. Although the photophysical mechanism falls beyond the scope of this work, the fluorescence data (Figures 1 and S27) suggest the existence of a grafting orientation dependence on the emission profiles. Likewise, the titrimetric association patterns found earlier (Figure S26) suggest the existence of aggregation derived from the binding events that take place on the Ni-Salphen complex component with a certain correlation with the grafting orientation employed. Therefore, we speculate that, upon association with Pd-PTA, it is plausible that the Ni-Salphen moieties could find it harder to proceed with their rotational movement over the C–O–C ester bridge, and this would occur differently for each grafting orientation evaluated in **4**, **5**, and **6**. In this manner, the grafting orientation would restrict the degree in which the free movement of the Ni-Salphen portions may occur, and this eventually might have repercussions in the long-range aggregation possibilities. Thus, it is reasonable to suppose that, for 1:1 stoichiometries, a more pronounced motion restriction in the unassociated PTA pendant terminals in each tethered Pd-PTA would modify the opportunity for dimerization schemes by entropic and enthalpic gains. The whole picture correlates with the effect seen on the fluorescence and absorbance values, perhaps through a progressive oligomerization as the most feasible scenario.

**Figure 1:**
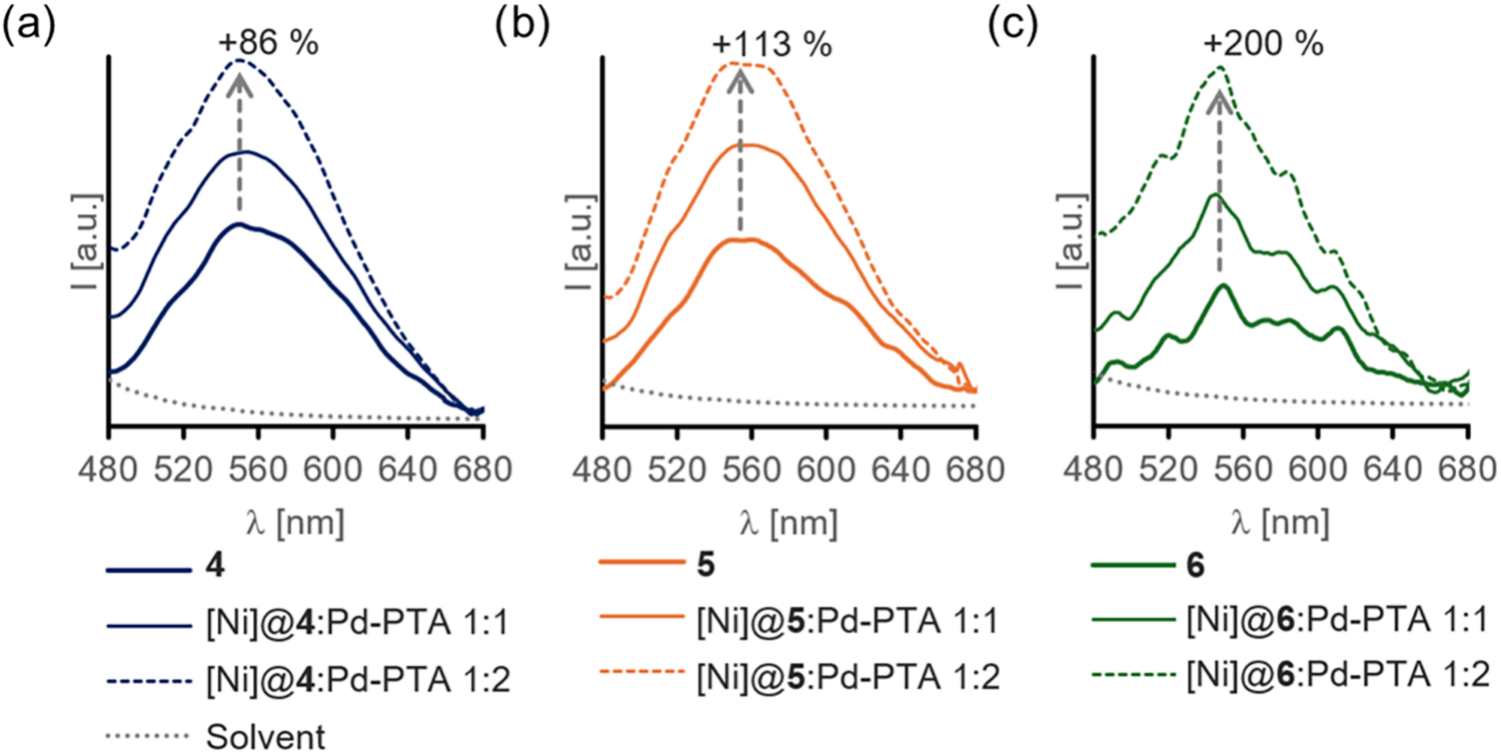
Fluorescence emission spectra of solutions from EOGO-*g*-[Ni-Salphen] materials **4** (a), **5** (b), and **6** (c) before (solid bold) and after additions of Pd-PTA at 1 equiv (solid narrow) and 2 equiv (dashed) to [Ni] concentration; gray dotted line indicates solvent used.

### Graphene Supramolecular Frameworks from EOGO-*g*-[Ni-Salphen] and Pd-PTA

To test the last hypotheses, we synthesized and analyzed solid-state supramolecular framework counterparts **10**-**12**, respectively, departing from solutions of **4**-**6** after their exposition to Pd-PTA (Scheme 1, Scheme S4). FT-IR spectra compared in a stepwise fashion (Figures S32, S37, S42) show for supramolecular frameworks **10**-**12** the bands attributed to the CN-C and PC-N vibrations (1239–1281 cm^–1^) from the PTA moiety of the Pd-PTA complex, while the NC-H vibration appears completely overlapped with the C–H band (2927 cm^–1^). On the other hand, we can also observe the bands from the grafted nanomaterial component from which each supramolecular framework is derived, such as ester C=O st. 1719–1722 cm^–1^, ester C–O st. 1224–1229 cm^–1^ and, finally, C=N st. from both imines, occurring at 1613–1621 cm^–1^ for the ketimines and at 1596–1602 cm^–1^ for the aldimines. As well, we found a marked long-range aggregation pattern in these products by scanning electron microscopy, SEM (Figure 2), that did not appear in the analysis of samples equally prepared from only EOGO, **4**, **5**, or **6**. These assemblies appeared for **10** and **11**, as irregularly shaped layered aggregates or, as in the form of micro- to mesoscale stratified structures for **12** with anisotropic aspect ratio, having dimensions of less than 20 μm in thickness and +10^2^ μm in width and depth (Figures S43 and 2i). EDS compositional mapping revealed the presence of O, N, Ni, Pd, P, and Cl (Figure 2) for **10**-**12**, with intensity variations matching the grain boundaries and particle locations, thus providing evidence for spatially selective grafting and reticulation. Likewise, TGA data supported degradation profiles in accordance with the available [Ni] units in EOGO-*g*-[Ni-Salphen] equimolarly reticulated by Pd-PTA components with degrees of reticulation of 63% for **10**, 37% for **11**, and 71% for **12** (Figures S29, S34, and S39). Furthermore, a structural repercussion of the Ni-Salphen component on the degradation temperatures was detected depending on the system, which facilitated an onset temperature for **12** (439 °C) located 86 °C earlier than that of **10** (525 °C), and this one occurring 55 °C before that of **11** (580 °C), meaning an easier combustion for the nanomaterial from the most accessible Ni-Salphen grafts. X-ray diffraction, XRD (Figures S28, S33, and S38), confirmed the interlayer spacing of 3.37 Å to be slightly higher than that of pristine layered graphene, as would be expected from flexible large sheets assembled through the edges that after stratification suffer deflection or flexural bending. Differential scanning calorimetry, DSC (Figures S29, S34, and S39), exhibited one glassy transition for each system, which we interpreted as the facility for the stratified products to be longitudinally displaced among their planes. The transition proceeded ca. 10 degrees earlier for **12** (220 °C) than that for **10** and **11**, which happened both at equivalent temperatures (228–231 °C). However, **12** also showed a narrow exothermic event at 283 °C, just a few degrees above the end-set of the change in heat capacity that corresponded to its *T*_g_, which could be associated with either residual reticulation or crystallization phenomena.

**Figure 2:**
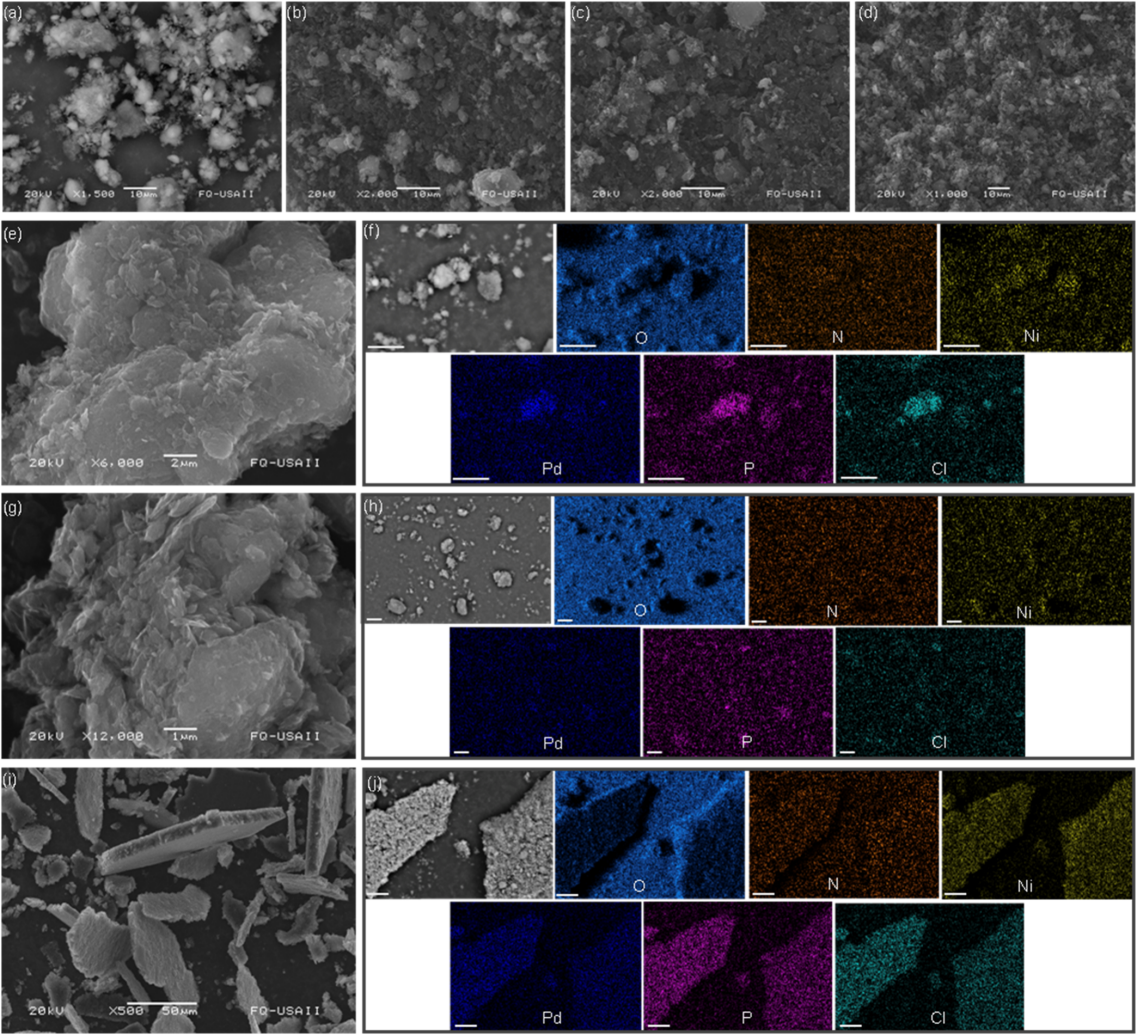
SEM micrographs of pristine EOGO (a), grafted nanomaterials **4** (b), **5** (c), and **6** (d), and supramolecular frameworks **10** (e), **11** (g), and **12** (i). Elemental mapping (EDS) for O, N, Ni, Pd, P, and Cl of the micrographs from selected zones of **10** (f), **11** (h), and **12** (j); scalebars in panel components: 10 μm.

**Scheme 1:**
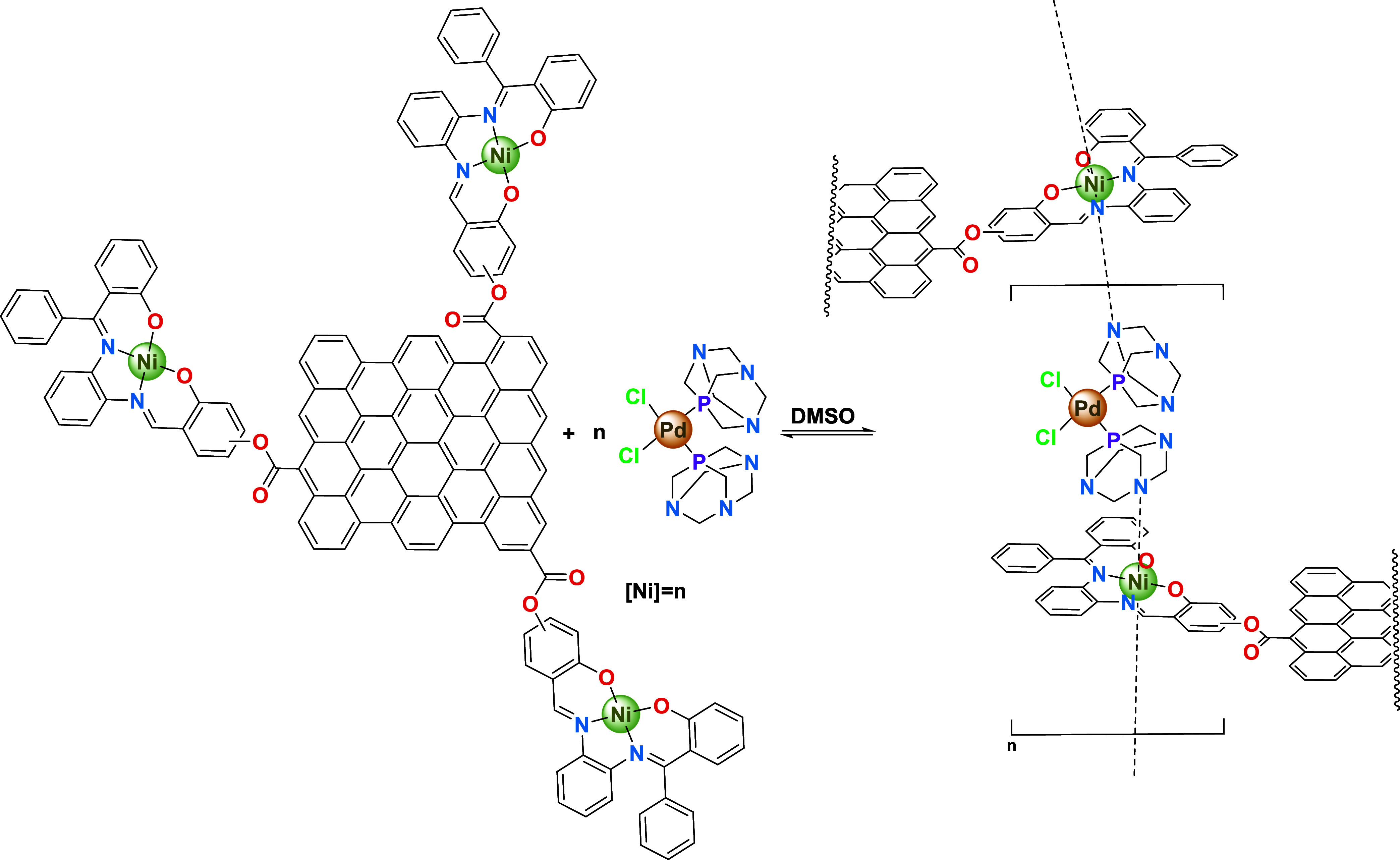
Synthesis of Graphene Supramolecular Frameworks (**10-12**) from EOGO-*g*-[Ni-Salphen] Variants (**4**-**6**) with Pd-PTA^a^

a The product shows an ideally extrapolated version of the most probable interatomic connectivity according to XPS results from **12**; note that the N atoms from PTA chosen to complete each octahedral coordination in the axial positions of [Ni] are chemically equivalent.

### Interatomic Connectivity of a Selected GSF System

Material history has proven to be decisive in the type of aggregation for each GSF at the micro- to mesoscale as much as in their thermoanalytical parameters. The latter suggests that the grafting orientation derived from the o-, m-, and p-substitution pattern at Ni-Salphen level plays a more significant role (than initially believed) in the yield efficiency of each stepwise product and, ultimately, on the overall properties of the final GSF. This in turn also suggests differences in the accessibility of the [Ni] binding sites toward Pd-PTA as a result of the grafting orientation as well. In this regard, **12** has gathered appealing results, showing not only the largest degrees of grafting (EOGO with **3** at 98%) and reticulation (**6** with Pd-PTA at 71%), but also calorimetric evidence of either noncovalent reticulation or crystallization which is, in any case, an indicative of a successful energy minimization in the components via supramolecular forces. Such a scenario is accompanied in **12** by the highest efficiency found in long-range anisotropic self-assembly in contrast to the counterparts from **10** or **11** that rather rendered isotropic aggregates. Therefore, we considered **12** and its nonreticulated nanomaterial **6** as model candidates for an in-depth assessment of the interatomic bonding profiles (Figures 3, S50, and S51, Table S1) using X-ray photoelectron spectroscopy (XPS).

**Figure 3:**
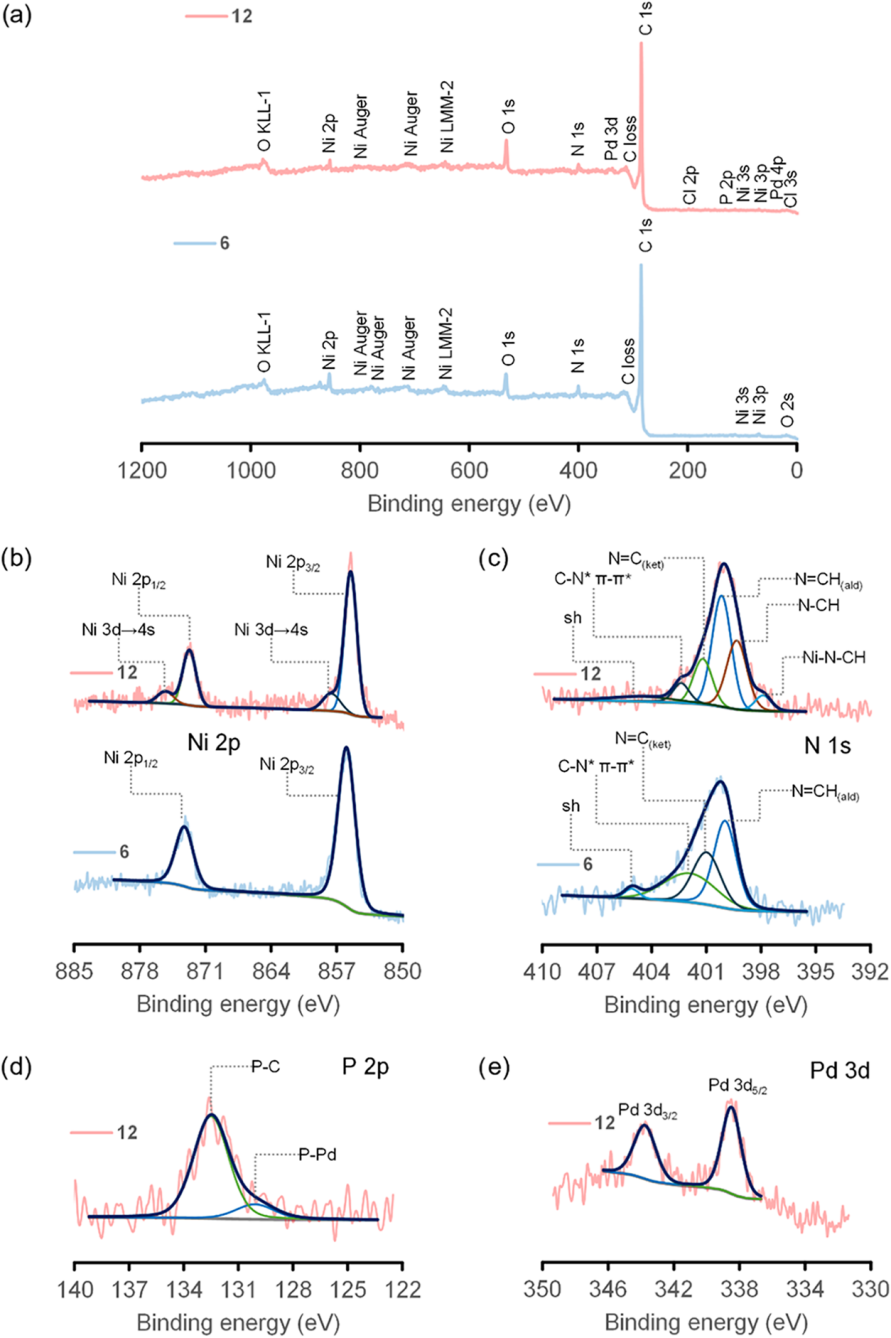
Selected XPS spectra of GSF **12** compared to its nonreticulated counterpart **6**. Survey spectra (a) and high-resolution spectra in the regions Ni 2p (b), N 1s (c), P 2p (d), and Pd 3d (e).

Survey spectra from both systems clearly exhibited the presence of Ni, N, O, and C as expected for EOGO sheets grafted to Ni-Salphen moieties (Figure 3), while **12** showed, in addition to the earlier composition, P, Pd, and Cl. The two materials exhibited minor peaks according to the elemental composition of each one (Ni Auger, O KLL-1, Ni KLMM-2, Ni 3s, Ni 3p, O 2s, Cl 3s, Cl 2p, Pd 4p, etc. in Figure 3a). However, high-resolution spectra of the regions Ni 2p, O 1s, N 1s, Pd 3d, C 1s, and P 2p were thoroughly deconvoluted (Figures 3b–e, S50 and S51) and the resulting peaks were cross-validated with the literature.^42–55^ This analysis afforded a comprehensive profile of interatomic connectivity (Table S1) before and after the formation of our selected GSF, which subsequently allowed us to structurally suggest a reasonable framework arrangement (Scheme 1).

The binding region corresponding to Ni 2p (Figure 3b) showed the coexistence of peaks in 873.25 eV (2p_1/2_) and 855.95 eV (2p_3/2_) for **6** and the absence of any satellite or shakeup. The latter demonstrated a square planar Ni^2+^ environment instead of an octahedral geometry, while the binding energy values correlated very well with other reports on similar Schiff base Ni (II) complexes.^42,43^ On the other hand, in **12**, the coexistence of peaks at 872.71 eV for 2p_1/2_ and 855.51 eV for 2p_3/2_ with contiguous shakeup transitions at 875.28 and 857.58 eV (3d→4s) is, on the contrary, evidence of an octahedral coordination arrangement for Ni^2+^.^44^ The low-energy shifted peaks in **12** (−0.54 eV for 2p_1/2_ and −0.44 eV for 2p_3/2_) could be on the other hand correlated with two additional contributions of electron density donors which apparently proceed axially to the metal center until the initially square planar arrangements developed into their octahedral counterparts.^45^ The changes observed are compatible with our proposal of two monodentate coordination events of Pd-PTA linkers occurring axially per each [Ni] center, one by face of the N_2_O_2_ square plane; this should be enabled by any of the available and chemically equivalent electron-donor N atoms in each PTA moiety.

Analysis of the O 1s region (Figure S50) showed single events for **6** and **12** that could be deconvoluted into seven peaks in both cases, all of them in good agreement with the literature,^42,43,45,46^ while these could then be sequentially assigned to O–C=O, O–Ni, C=O, C–O–C, two O–C for the metal-bonding N_2_O_2_ cavity, and one satellite. Typical Schiff base coordination compound O 1s shakeup satellites (*sh*) appeared at 538.54 eV for **6** and 538.07 eV for **12**.^45^ Metal-bonded O–C appeared at 535.79 and 534.84 eV for **6**, at 535.17 and 534.33 eV for **12**, being the higher binding energy (BE) ascribed to the oxygen atom located at the ketimine portion,^42,43,45^ whereas the lower BE to the respective one at the aldimine^42,43^ counterpart. Binding energies of 531.55 and 531.25 eV represent the oxygen bonding with Ni (O–Ni), respectively, of **6** and **12**.^42,43^ The remaining peaks consistently matched those of graphene-derivative ester groups: C–O–C at 533.91 eV for **6** and 533.4 eV for **12**, C=O at 532.74 eV for **6** and 532.23 eV for **12**, and finally, O–C=O at 530.31 eV for **6** and 530.49 eV for **12**.^46^

The high-resolution XPS spectra of the core level in the N 1s region (Figure 3c) could be fitted to four peaks for nanomaterial **6** and to six peaks for GSF **12** in accordance with earlier publications.^42,43,45,47–49^ The energy positions of the shakeup satellites are 405.08 eV for **6** and 404.42 eV for **12**,^45^ while the peaks that appear at 401.90 eV for **6** and at 402.38 eV for **12**, correspond to the π bond in each of the imine groups that undergo π-π* type processes (C–N*).^42,43^ The peaks at 400.98 and 399.97 eV from **6** and at 401.19 and 400.16 eV from **12** can be identified as nitrogen bonding with carbon in either the ketimine (N = C) or the aldimine (N=CH) form.^42,43,45,47^ The additional contributions found for **12** at the binding energies 399.33 and 397.89 eV are ascribed to amine-type nitrogen–carbon bonding in two forms, the former from free tertiary amine groups in the PTA component,^47–49^ while the latter is produced by the tertiary amine groups being coordinated to the Ni centers.^47^

A careful analysis of the high-resolution spectra of the portions Pd 3d_3/2_ and Pd 3d_5/2_ in **12** (Figure 3e) allowed us to confirm the prevalence of Pd­(II) chemical state together with an absence of adventitious Pd(0).^50^ Binding energies of the peaks in these regions appeared at 343.77 and 338.50 eV, respectively. These values extracted from the deconvolution profile were in good agreement with earlier reports of compatible systems containing Pd­(II) bonded to bidentate phosphine-type P-donors and Cl^51^ as well as with Pd­(II)­Cl_2_ *cis*-coordinated to P-donor-containing ligands^50^ in a square planar geometry fashion. The picture is consistent with the preservation of a core Pd-PTA structure identical to the free complex^29,71^ under a *cis*–P–Pd-P square planar geometry in **12** and, at the same time, shows no evidence of ligand exchange, degradation, or coordinative rearrangement at the PTA portions.

The C 1s spectral region was fitted to six peaks for **6** and seven peaks for **12** (Figure S51). The binding energies of 291.04, 287.72, 287.01, 286.20, and 284.81 eV of the former, and BE of 290.50, 287.64, 286.96, 286.15, and 284.82 eV of the latter, are in close match with the literature.^46^ These BE values are respectively associated with graphene component groups π-π* satellite bonds, O–C=O, C=O, C–O, and C–C/C=C for both systems in that order. The peak with BE values of 285.56 eV in **6** and 285.55 eV in **12** are both ascribed to C=N/C–N groups^47,49,52^ that could arise from either the imine groups or the PTA moieties, whereas the peak at 283.76 eV in **12** could be correlated with C–P bonding^48,53^ as shown in earlier reports.

Finally, the deconvolution analysis of the high-resolution spectrum of P 2p revealed two types of chemical species, P–C and P–Pd, as well as the absence of oxidized phosphorus.^47,48,51–55^ The peak at binding energy of 132.49 eV is compatible with reports on phosphorus bonded to carbon (P–C).^47,48,51,53–55^ On the contrary, the peak located at a lower binding energy value of 130.08 eV could correspond to phosphorus bonded to palladium centers.^47,48,51^

In view of the titration equivalence point found close to the unit for **6** vs Pd-PTA and the equimolar Ni/Pd metal composition found earlier in **12**, the most probable scenario is that a high fraction of Pd-PTA units in our GSF were shared each by two [Ni] centers and, in the same manner, a high proportion of [Ni] centers also proceed each with two Pd-PTA, while everything occurs through an octahedral geometry. Therefore, we believe that it is likely that GSF assembly will proceed by octahedral concatenation of Pd-PTA units and [Ni] centers alternately until a ratio close to 1:1 was reached, as long as a suitable accessibility for successive binding events still subsists. Scheme 1 indicates the last proposal, where the concatenated elements represent a repetitive unit, as shown in brackets. We believe that at least for our model system, this framework could be valid and is the motive force for the anisotropic aggregation found for **12**.

## Conclusions

With the data from titration, fluorimetry, electron microscopy, elemental composition, infrared spectroscopy, NMR, calorimetry, thermogravimetry, X-ray diffraction, and photoelectron spectroscopy, we have demonstrated the possibility to prepare a new family of supramolecular frameworks using graphene as a 2D building block. We showed that the framework growth likely proceeded by supramolecular reticulation of the host Ni-Salphen complexes bonded covalently to the edges of graphene using [PdCl_2_(3,5,7-triaza-7-phosphaadamantane)_2_] complex as a suitable N-donor ditopic guest linker. Reticulation of the framework also was possible via repetitive units octahedrally interconnected through axial N–Ni–N portions and *cis*-type P–Pd–P linkers enabled by 3,5,7-triaza-7-phosphaadamantane moieties.

Furthermore, so far, a clear correlation emerged between anisotropy, structural regularity, reticulation efficiency, and cooperativity in the construction of this new type of supramolecular frameworks. Notably, this correlation also coincided with the higher grafting degrees of the nonreticulated hybrid versions, where these features corresponded to the highest host–guest accessibility and, at least in appearance, to the rotational freedom granted by the hydroxyl substitution pattern in the Ni-Salphen moiety used. Furthermore, this correlation is apparently extended to emission effects triggered by host–guest phenomena, which are observed more steeply for the highest reticulation degrees achieved and, to this point, seem to have a certain degree of kinetic implications. It is interesting to highlight that the overall scenario in combination with the observed equimolar stoichiometric profile and the interatomic connectivity found in the selected systems strongly suggests an interplay among self-assembly, host availability, and adaptability that also reveals plausible in bidimensional counterparts for [-A-B-]*_n_* systems.

All these phenomena, alongside with the tailored evolution in the respective binding constants of the Ni-Salphen scaffold variants, reveal not only a quantitative association of Pd-PTA complexes per [Ni] binding unit, but strongly suggest a breakthrough in framework design as the one proposed in this work, that might compete with the current drawbacks in porous analogues such as MOFs, covalent organic frameworks (COFs), hydrogen-bonded organic frameworks (HOFs), etc.^56–58^ The possibility to achieve long-range carbon-based auto-organized morphologies from 2D components, as those shown for the supramolecular frameworks, triggered by Pd-PTA and controlled in their anisotropy and efficiency by the scaffold’s grafting orientation, represent a straightforward approach that opens further opportunities not yet covered by current multifunctional materials with optoelectronic, catalytic, energy-harvesting, water-splitting or environmental remediation properties.^59–69^ However, further research must be done in this regard to answer the yet unsolved questions related to the reticulation process mechanism and kinetics, the photophysical mechanisms underlying the changes in emission and their correlation to cooperativity, and applicative details such as modularity and deployment.

## Methods

### General

All reagents were obtained from commercial sources and used without any further purification, with the exception of (*E*)-2-(((2-aminophenyl)­imino)­(phenyl)­methyl) phenol (***I***) and Pd-PTA, which were synthesized following previously reported methods.^70,71^ EOGO was acquired from Sigma-Aldrich with a certified oxygen content of 4.8% wt., equivalent to 1.5 mmol g^–1^ of carboxylic acid groups; d_50_ = 4.2 μm, span = 3.2. All NMR measurements were carried out on either a Varian VNMRS 400 MHz spectrometer or a Jeol ECZ600R 600 MHz spectrometer at room temperature unless otherwise stated, and chemical shifts are given in parts per million vs TMS. High-resolution MS (APCI-TOF) measurements were acquired via a PerkinElmer AxION 2 TOF. Infrared spectra were recorded on a PerkinElmer FT-IR Spectrum RXI equipment. Powder X-ray diffraction measurements were acquired via a D8 ADVANCE DAVINCI X-ray diffractometer with Cu *K*_a_ radiation wavelength (λ) = 1.5405 Å. Thermogravimetric analyses (TGA) were performed under air flow with a ramp of 10 °C min^–1^ on a PerkinElmer TGA400 apparatus; starting temperature 25 °C, final temperature 800 °C.^72,73^ Differential scanning calorimetry (DSC) measurements were performed under air atmosphere with a ramp of 10 °C min^–1^ on a Mettler Toledo DSC1 calorimeter; conditioning cycles: 25 to 130 °C, isothermal at 130, 130 to −20 °C, isothermal at −20 °C; Measuring cycle: −20 to 600 °C.^72,73^ All SEM determinations were acquired with a JEOL JSM-5900-LV instrument with an Oxford Aztek 100 EDS detection system. All characterization techniques were performed at the Unidad de Servicios de Apoyo a la Investigación y a la Industria (USAII) of the School of Chemistry, UNAM. X-ray photoelectron spectroscopy analyses were performed with a PHI 5000 Versa Probe II Scanning XPS Microprobe spectrometer. All UV–vis titration experiments were performed with an XB-10 Dynamica Halo spectrophotometer. All spectrofluorimetric experiments were carried out with an F96PRO Hinotek apparatus equipped with an emission filter of λ = 365 nm.

### Synthesis and Characterization of Ni-Salphen Complexes

All Ni-Salphen complexes were synthesized using the procedure described previously for **1**,^18^ with variations only in the reactant dihydroxybenzaldehyde used, in order to produce the different positioning of the hydroxy group in the different products.

Complex **1** is a greenish-brown solid (256 mg, > 99%); *T*_m_ (DSC) = 283.20 °C (281.3–284.9 °C); *T*_d_ (TGA): *T*_O_ = 336.63 °C, *T*_f_ = 506.70 °C, *T*_p1_ = 370.62 °C, *T*_p2_ = 454.38 °C, *T*_p3_ = 477.64 °C (first derivative peaks); moisture 4.05%; drops p1-p3:11.02%, 21.69%, 48.20%; metal residues determined 15.69%, requires 16.06% (NiO), estimated purity 97.7%.

Complex **2** is a purplish-brown solid (394 mg, 81%); ^1^H NMR (600 MHz, DMSO-*d*_6_)­δ = 10.23 (s, 1H), 8.50 (s, 1H), 7.83 (d, 1H, ^3^ *J*_HH_ = 8.22 Hz), 7.57 (t, 1H, ^3^ *J*_HH_ = 7.53), 7.52 (t, 2H, ^3^ *J*_HH_ = 7.50 Hz), 7.37 (d, 1H, ^3^ *J*_HH_ = 8.70 Hz), 7.33 (d, 2H, ^3^ *J*_HH_ = 7.62 Hz), 7.20 (t, 1H, ^3^ *J*_HH_ = 7.89 Hz), 7.00 (t, 1H, ^3^ *J*_HH_ = 7.53 Hz), 6.85 (d, 1H, ^3^ *J*_HH_ = 8.52 Hz), 6.78 (d, 1H, ^3^ *J*_HH_ = 8.40 Hz), 6.55 (t, 1H, ^3^ *J*_HH_ = 8.01 Hz), 6.42 (t, 1H, ^3^ *J*_HH_ = 7.65 Hz), 6.21 (d, 1H, ^3^ *J*_HH_ = 8.98 Hz), 6.17 (s, 1H), 6.15 (d, 1H, ^3^ *J*_HH_ = 8.52 Hz) ppm. ^13^C {^1^H} NMR (100 MHz, DMSO-*d*_6_), δ = 169.2, 166.2, 164.6, 153.7, 144.2, 142.7, 136.6, 135.8, 134.1, 133.1,129.8, 129.0, 126.6, 124.4, 123.2, 122.9, 121.4, 115.6, 114.4, 107.8, 103.6 ppm. FT-IR (ATR)/ cm^–1^: 3373 (ν_ar_ O–H, st.), 3057 (ν_ar_ C–H, st.), 1598 (ν C = N_ketimine_, st.), 1546 (ν C = N_aldimine_, st.), 1242 and 1213 (ν_ar_ C–OH, st.). APCI-TOF: *m*/*z* 465.0779 *m*/*z* [Ni-Salphen]^+^, C_26_H_18_N_2_NiO_3_ requires 465.13440. *T*_d_ (TGA): *T*_O_ = 164.66 °C, *T*_f_ = 512.14 °C, *T*_p1_ = 196.75 °C, *T*_p2_ = 255.04 °C, *T*_p3_ = 364.68 °C, *T*_p4_ = 397.82 °C, T_p5_ = 441.85 °C, T_p6_ = 476.26 °C (first derivative peaks); moisture 0.43%; drops p1-p6 3.86, 2.19, 2.38, 4.47, 13.29, 57.45%; metal residues determined 16.00%, requires 16.06% (NiO), estimated purity 99.6%.

Complex **3** is a dark brown solid (422 mg, 85%); ^1^H NMR (400 MHz, DMSO-*d*_6_), δ = 8.80 (s, 1H), 8.61 (s, 1H), 7.93 (d, 1H, ^3^ *J*_HH_ = 8.24 Hz), 7.59–7.51 (m, 3H), 7.33 (d, 2H, ^3^ *J*_HH_ = 7.04 Hz), 7.19 (ddd, 1H, ^3^ *J*_HH_ = 7.12 Hz, ^3^ *J*_HH_ = 8.48 Hz, ^4^ *J*_HH_ = 1.12 Hz), 7.01 (t, 1H, ^3^ *J*_HH_ = 7.60 Hz), 6.90 (dd, 1H, ^3^ *J*_HH_ = 9.00 Hz, ^4^ *J*_HH_ = 2.96 Hz), 6.87 (d, 1H, ^4^ *J*_HH_ = 2.96 Hz), 6.83 (d, 1H, ^3^ *J*_H–H_ = 8.44 Hz), 6.77 (dd, 1H, ^3^ *J*_HH_ = 8.40 Hz, ^4^ *J*_HH_ = 0.88 Hz), 6.73 (d, 1H, ^3^ *J*_HH_ = 9.00 Hz), 6.58 (t, 1H, ^3^ *J*_HH_ = 7.76 Hz), 6.41 (t, 1H, ^3^ *J*_HH_ = 7.42 Hz), 6.16 (d, 1H, ^3^ *J*_HH_ = 8.40 Hz) ppm. ^13^C­{^1^H} NMR (100 MHz, DMSO-*d*_6_), δ = 169.3, 166.3, 159.7, 154.6, 146.4, 143.9, 143.0, 136.5, 134.2, 133.2, 129.8, 129.0, 126.9, 126.6, 125.1, 123.2, 122.9, 121.4, 120.8, 119.5, 116.1, 114.7, 114.3 ppm. FT-IR (ATR)/ cm^–1^: 3086 (ν_ar_ O–H, st.), 3059 (ν_ar_ C–H, st.), 1629 (ν C=N_ketimine_, st.), 1594 (ν C=N_aldimine_, st.), 1244 and 1200 (ν_ar_ C–OH, st.). APCI-TOF: 465.0660 *m*/*z* [Ni-Salphen]^+^, and C_26_H_18_N_2_NiO_3_ requires 465.13440. *T*_d_ (TGA): *T*_O_ = 331.58 °C, *T*_f_ = 491.39 °C, *T*_p1_ = 341.89 °C, *T*_p2_ = 395.97 °C, T_p3_ = 461.3 °C (first derivative peaks); moisture 0.06%; drops p1-p3 1.64, 6.49, 75.85%; metal residues determined 15.97%, requires 16.06% (NiO), estimated purity 99.5%.

### Synthesis and Characterization of EOGO-*g*-[Ni-Salphen] Grafted Nanomaterials

All grafted nanomaterials were synthesized according to the procedure described previously for **4**,^18^ with variations only in the Ni-Salphen complex used (**1**–**3**), to achieve the different grafting positions on the nanomaterials. With that in mind, we present the general method for the synthesis of EOGO-*g*-[Ni-Salphen] grafted nanomaterials.

#### Grafted Nanomaterial **4** (207 mg)

*T*_d_ (TGA): *T*_O_ = 379.40 °C, *T*_f_ = 604.61 °C, *T*_p1_ = 411.87 °C, *T*_p2_ = 587.78 °C (first derivative peaks), residue (799.93 °C) 0.62%, residue (*T*_f_) 3.66%; moisture 1.45%; Ni-Salphen content determined 37.26%, requires 40.23%, estimated grafting degree 93%. *T*_g_ (DSC): 230.75 °C. XRD (Cu *K*α_1_ = 0.15406 nm, 2θ [°], (d [Å])): 26.48 (3.36), 54.75 (1.68), 77.54 (1.23).

#### Grafted Nanomaterial **5** (211 mg)

FT-IR (ATR)/ cm^–1^: 2966 (ν C–H, st.), 1728 (ν_ar_ C=O, st.), 1595 (ν C=N_ketimine_, st.), 1532 (ν C=N_aldimine_, st.), 1219 (ν C–O, st.). Td (TGA): *T*_O_ = 219.99 °C, *T*_f_ = 547.26 °C, *T*_p1_ = 236.53 °C, *T*_p2_ = 405.79 °C, *T*_p3_ = 510.53 °C (first derivative peaks), residue (799.94 °C) 0.70%, residue (*T*_f_) 5.38%; moisture 1.49%; Ni-Salphen content determined 38.24%, requires 40.23%, estimated grafting degree 95%. XRD (Cu Kα_1_ = 0.15406 nm, 2θ [°], (d [Å])): 26.49 (3.36), 53.84 (1.70), 76.96 (1.24).

#### Grafted Nanomaterial **6** (268 mg)

FT-IR (ATR)/ cm^–1^: 3065 (ν C–H, st.), 1698 (ν_ar_ C=O, st.), 1647 (ν C=N_ketimine_, st.), 1580 (νald. C=N_aldimine_, st.), 1235 (ν C–O, st.). *T*_d_ (TGA): *T*_O_ = 384.74 °C, *T*_f_ = 678.87 °C, *T*_p1_ = 390.15 °C, *T*_p2_ = 401.78 °C (first derivative peaks), residue (799.92 °C) 2.98%, residue (*T*_f_) 5.51%; moisture 1.38%; Ni-Salphen content determined 39.59%, requires 40.23%, estimated grafting degree 98%. *T*_g_ (DSC): *T*_g1_ = 165.75 °C, and *T*_g2_ = 227.58 °C. XRD (Cu Kα_1_ = 0.15406 nm, 2θ [°], (d [Å])): 26.47 (3.36), 54.54 (1.68), 77.54 (1.23).

### UV–Vis Spectrophotometric Titrations of [Pd-PTA]/[Ni-Salphen] Systems

For these systems, the host species (H) is Pd-PTA and the guest species (G) is the respective Ni-Salphen complex (**1**–**3**). In a typical experiment, 1.0 mL of guest solution in DMSO (6.0 × 10^–4^ mol L^–1^ nominal) was added in aliquots of 25.0 μL to 2.0 mL of host solution (2.0 × 10^–5^ mol L^–1^ nominal) contained on a 1.00 cm optical length quartz cuvette. UV–vis spectrum was acquired after each addition in a wavelength range of 250–650 nm; absorbance and H/G concentrations were corrected for dilution. From the absorbance data, selected wavelengths were used to determine predominant H/G stoichiometries and binding constants (*K*_11_ and *K*_12_) by using Bindfit software^39–41^ and linear least squares^72–74^ together with intersection analysis; wavelengths were selected via a case-by-case analysis to avoid undesirable absorbance contributions.

### Synthesis and Characterization of [Pd-PTA]/[Ni-Salphen] Supramolecules

[Pd-PTA]/[Ni-Salphen] supramolecules (**7**–**9**) were synthesized by incubation of Pd-PTA with Salphen variants (**1**–**3**). For the synthesis of supramolecular complexes (**7**–**9**), Pd-PTA (0.028 mmol) and the respective Ni-Salphen complex (0.056 mmol) were added to a 5 mL vial and later dissolved in 250 μL of DMSO with the help of a tip sonicator. System was allowed to dry under vacuum and further dried with a rotary evaporator at 65 °C for 16 h.

#### Supramolecule **7** (20 mg)

Brownish-green solid. *T*_d_ (TGA): *T*_O_ = 272.86 °C, *T*_f_ = 689.02 °C, *T*_p1_ = 291.85 °C, *T*_p2_ = 312.62 °C, *T*_p3_ = 330.0 °C, *T*_p4_ = 499.37 °C, *T*_p5_ = 678.88 °C (first derivative peaks); drops p1-p3 21.17%; drops p4-p5 45.19, 2.97%; moisture 5.11%, residual DMSO 2.21%, metal residues determined 24.25%, requires 22.35% (Pd:Ni 1:2).

#### Supramolecule **8** (20 mg)

Red solid. *T*_d_ (TGA): *T*_O_ = 200.09 °C, *T*_f_ = 643.66 °C, *T*_p1_ = 209.88 °C, *T*_p2_ = 302.18 °C, *T*_p3_ = 351.03 °C, *T*_p4_ = 485.50 °C, *T*_p5_ = 624.94 °C (first derivative peaks); drop p1 10.50%, drops p2-p3 9.65%, drop p4 51.47%, drop p5 2.52%; moisture 4.36%, residual DMSO 2.34%; metal residues determined 20.01%, requires 22.35% (Pd:Ni 1:2).

#### Supramolecule **9** (20 mg)

Brown solid. *T*_d_ (TGA): *T*_O_ = 254.15 °C, *T*_f_ = 661.01 °C, *T*_p1_ = 265.52 °C, *T*_p2_ = 304.32 °C, *T*_p3_ = 481.86 °C, *T*_p4_ = 621.92 °C (first derivative peaks); drops p1-p2 18.40%, drop p3 25.17%, drop p4 14.7%; moisture 12.16%, residual DMSO 5.47%; metal residues determined 23.81%, requires 22.35% (Pd:Ni 1:2)

### UV–Vis Spectrophotometric Titrations of EOGO-*g*-[Ni-Salphen]/Pd-PTA Systems

For the study of the systems in which the Ni-Salphen binding units grafted onto the nanomaterials (**4**–**6**) ([Ni]) play the role of host and Pd-PTA complex is the guest, on a typical experiment, 1 mL of guest solution in DMSO (5.2 × 10^–5^ mol L^–1^ nominal) was added, in different aliquots, to 2.0 mL of host solution in DMSO (0.05 mg mL^–1^ nominal, 1.5 × 10^–5^ mol L^–1^ nominal of [Ni]), utilizing a 1.00 cm optical length quartz cuvette; [Ni] nominal content was estimated from stoichiometric conversion of carboxylic acid groups as of FT-IR results, validated by volumetry and TGA. UV–vis spectra were acquired after each aliquot addition in a wavelength range of 250–750 nm; absorbance and H/G concentrations were corrected for dilution. From the absorbance data, selected wavelengths were used to determine predominant H/G stoichiometries and binding constants (*K*_11_ and *K*_12_) by using Bindfit online software^39–41^ and linear least squares^72–74^ together with intersection analysis; wavelengths were selected via a case-by-case analysis to avoid undesirable absorbance contributions.

### Fluorimetric Analysis of EOGO-*g*-[Ni-Salphen]/Pd-PTA Systems

The study of systems composed of Ni-Salphen grafted nanomaterials (**4**–**6**) was carried out by the addition of guest solution in spectroscopic grade THF (Pd-PTA, 1.0 × 10^–4^ mol L^–1^ nominal) to 2.0 mL of host solution in spectroscopic grade THF (0.05 mg mL^–1^, [Ni] = 1.5 × 10^–5^ mol L^–1^ nominal) in a 1.00 cm optical length quartz cuvette. Emission spectra were acquired before and after each aliquot addition, at stoichiometries 1:1 and 1:2, in a wavelength range of 480–680 nm using an excitation filter of 365 nm (±5 nm) at 300 nm min^–1^ and constant photomultiplier tube gain 14 for comparative assessment purposes (Figure 1). Solvent background and scattering was considered for normalized analyses.

Kinetically monitored fluorimetric experiments were carried out under similar conditions, using a single emission wavelength of 548 nm and collected at intervals of 0.5 s at stoichiometries 1:1 and 1:2 (Figure S27).

### Synthesis and Characterization of Supramolecular Framework

Synthesis of the supramolecular frameworks was performed by adding the corresponding EOGO-*g*-[Ni-Salphen] nanomaterial ([Ni] = 0.004 mmol) and Pd-PTA connector to a 5 mL vial, and the latter was later dissolved in 250 μL of DMSO with a tip sonicator. System was allowed to dry under vacuum and further dried with a rotary evaporator and left at 65 °C for 16 h. All of the supramolecular frameworks produced have the appearance of black solids.

#### Supramolecular Framework **10** (85 mg)

FT-IR (ATR) cm^–1^: 2939–2869 (ν C–H, st.), 1722 (ν C=O), 1613 (ν C=N_ketimine_, st.), 1602 (ν C=N_aldimine_, st.), 1282 (ν C–N), 1229 (ν C–O). *T*_d_ (TGA): *T*_O_ = 525.59 °C, T_F_ = 713.68 °C, *T*_p1_ = 619.66 °C, *T*_p2_ = 682.81 °C (first derivative peaks); drops p1-p2 87.60%; moisture 1.75%, DMSO 2.97%; metal content determined 8.06%, requires 9.52% (Ni/Pd equimolar); estimated degree of reticulation 63%. *T*_g_ (DSC): *T*_g1_ = 231.4 °C, *T*_g2_ = 313.3 °C. XRD (Cu *K*α_1_ = 0.15406 nm, 2θ [°], (d [Å])): 26.44 (3.37), 54.52 (1.68), 77.48 (1.23).

#### Supramolecular Framework **11**(85 mg)

FT-IR (ATR)/ cm^–1^: 2973–2868 (ν C–H, st.), 1719 (ν C=O), 1619 (ν C=N_ketimine_, st.), 1601 (ν C=N_aldimine_, st.), 1294 (ν C–N), 1224 (ν C–O). *T*_d_ (TGA): *T*_O_ = 580.95 °C, *T*_F_ = 684.32 °C, *T*_p1_ = 653.76 °C (first derivative peak); drop p1 88.37%; moisture 2.02%, DMSO 2.96%; metal content determined 7.00%, requires 9.77% (Ni/Pd equimolar); estimated degree of reticulation 37%. *T*_g_ (DSC): *T*_g1_ = 228.2 °C, and *T*_g2_ = 491.8 °C. XRD: (Cu Kα_1_ = 0.15406 nm, 2θ [°], (d [Å])): 26.44 (3.37), 54.56 (1.68), 77.50 (1.23).

#### Supramolecular Framework **12** (85 mg)

FT-IR (ATR)/ cm^–1^: 2964–2870 (ν C–H, st.), 1722 (ν C=O), 1621 (ν C=N_ketimine_, st.), 1596 (νald. C=N_aldimine_, st.), 1289 (ν C–N), 1218 (ν C–O). *T*_d_ (TGA): *T*_O_ = 439.75 °C, T_F_ = 705.53 °C, *T*_p1_ = 507.34 °C, *T*_p2_ = 638.16 °C (first derivative peak); drop p1 38.61%, drop p2 47.60%; moisture 1.95%, DMSO 3.52%; metal content determined 8.80%, requires 10.12% (Ni/Pd equimolar); estimated degree of reticulation 71%. DSC: *T*_g_ = 220.1 °C; *T*_p(exo)_ = 283 °C. XRD: (Cu Kα_1_ = 0.15406 nm, 2θ [°], (d [Å])): 26.40 (3.37), 54.34 (1.69), 77.52 (1.23).

### Hill Plots

All the Hill plots were prepared from spectrophotometric titration data reported in Figures S22 and S26 and species distribution values from Bindfit estimations.^39–41^ In all cases, Y is the occupancy parameter and the slope is the Hill coefficient (η_H_);^75^ Ln­[G] is the natural logarithm of the concentrations at the equilibrium for **1**-**3** (Figures S44–S46) or Pd-PTA (Figures S47–S49).

## Supplementary Material



## Notes

Schemes and figures corresponding to the synthesis, and characterizations and diverse experiments conferred in the present article (PDF)

## Abbreviations

EOGO: Edge Oxidized Graphene Oxide
GSFs: Graphene Supramolecular Frameworks
Pd-PTA: [PdCl_2_(3,5,7-triaza-7-phosphaadamantane)_2_]
S_N_Ac: nucleophilic acyl substitution
H/G: host–guest
PTA: 3,5,7-triaza-7-phosphaadamantane
MOFs: metal–organic frameworks
TGA: thermogravimetric analysis
FT-IR: fourier transform infrared
CI: confidence interval
DSC: differential scanning calorimetry
*T*_m_: fusion temperature
*T*_p_: peak temperature
SEM: scanning electron microscopy
EDS: energy-dispersive X-ray spectroscopy
XRD: X-ray diffraction
*T*_g_: glass transition temperature
XPS: X-ray photoelectron spectroscopy
COFs: covalent organic frameworks
HOFs: hydrogen-bonded organic frameworks
NMR: nuclear magnetic resonance
APCI: atmospheric pressure chemical ionization
TOF: time-of-flight

## References

[1] Han K., Zhang Z., Tezcan F. A. (2023). Spatially Patterned, Porous Protein
Crystals as Multifunctional Materials. J. Am.
Chem. Soc..

[2] Gao X., Yang X., Lv J., Zhao L., Sui X., Zhang X., Xie Y., Tang Z. (2024). Induced Huge Optical
Activity in Nanoplatelet Superlattice. J. Am.
Chem. Soc..

[3] Farsheed A. C., Zevallos-Delgado C., Yu L. T., Saeidifard S., Swain J. W. R., Makhoul J. T., Thomas A. J., Cole C. C., Huitron E. G., Grande-Allen K. J., Singh M., Larin K. V., Hartgerink J. D. (2024). Tunable Macroscopic Alignment of Self-Assembling Peptide
Nanofibers. ACS Nano.

[4] Wu B., Fan J.-Z., Han J.-Y., Su Y., Zhou M.-P., Sun J.-H., Gao Y., Chen S., Wu J.-J., Wang Z.-S., Wang X.-D. (2023). Dynamic Epitaxial Growth of Organic
Heterostructures for Polarized Exciton Conversion. Adv. Mater..

[5] Bak Y., Park G., Hong T., Lee C., Lee H., Bae T.-H., Park J. G., Yoon D. K. (2023). Utilization of Physical
Anisotropy in Metal–Organic Frameworks via Postsynthetic Alignment
Control with Liquid Crystal. Nano Lett..

[6] Li S., Aizenberg M., Lerch M. M., Aizenberg J. (2023). Programming
Deformations of 3D Microstructures: Opportunities Enabled by Magnetic
Alignment of Liquid Crystalline Elastomers. Acc. Mater. Res..

[7] Escárcega-Bobadilla M. V., Maldonado-Domínguez M., Romero-Ávila M., Zelada-Guillén G. A. (2022). Turing patterns
by supramolecular
self-assembly of a single salphen building block. iScience.

[8] Escárcega-Bobadilla M. V., Zelada-Guillén G. A., Pyrlin S. V., Wegrzyn M., Ramos M. M. D., Giménez E., Stewart A., Maier G., Kleij A. W. (2013). Nanorings and rods
interconnected by self-assembly
mimicking an artificial network of neurons. Nat. Commun..

[9] Oliveira E.
Y. S., Bode R., Escárcega-Bobadilla M. V., Zelada-Guillén G. A., Maier G. (2016). Polymer nanocomposites from self-assembled polystyrene-grafted carbon
nanotubes. New J. Chem..

[10] Arenas-García J., Escárcega-Bobadilla M. V., Zelada-Guillén G. A. (2018). Grafting
Multiwalled Carbon Nanotubes with Polystyrene to Enable Self-Assembly
and Anisotropic Patchiness. J. Vis. Exp..

[11] Zelada-Guillén G. A., Escárcega-Bobadilla M. V., Wegrzyn M., Giménez E., Maier G., Kleij A. W. (2018). Enhanced Conductivity for Carbon
Nanotube Based Materials through Supramolecular Hierarchical Self-Assembly. Adv. Mater. Interfaces.

[12] Park J., Kim Y. S., Sung S. J., Kim T., Park C. R. (2017). Highly
dispersible edge-selectively oxidized graphene with improved electrical
performance. Nanoscale.

[13] Cho Y. M., Kim K. T., Lee G. S., Kim S. H. (2019). The role of edge-oxidized
graphene to improve the thermopower of p-type bismuth telluride-based
thick films. Appl. Surf. Sci..

[14] Jung S.-H., Kim K. T., Lee G.-S., Sung J.-Y., Kim D. W., Eom Y. S., Yang D. Y., Yu J., Park J. M., Hyeon D. Y., Park K.-I. (2021). Synergistically
Improved Thermoelectric
Energy Harvesting of Edge-Oxidized-Graphene-Bridged N-Type Bismuth
Telluride Thick Films. ACS Appl. Mater. Interfaces.

[15] Tarannum F., Muthaiah R., Danayat S., Foley K., Annam R. S., Walters K. B., Garg J. (2022). Chemically
Edge-Carboxylated Graphene
Enhances the Thermal Conductivity of Polyetherimide–Graphene
Nanocomposites. ACS Appl. Mater. Interfaces.

[16] Ghosh A., Seth S. K., Purkayastha P. (2018). Undulation induced tuning of electron
acceptance by edge-oxidized graphene oxide. Spectrochim. Acta, Part A.

[17] Alanazi H., Alharbi Y. R., Abadel A. A., Elalaoui O. (2022). Effect of edge oxidized
graphene oxide on micro and macromechanical properties and microstructure
of cement paste. Int. J. Mater. Res..

[18] Hernández-Pacheco P., Zelada-Guillén G. A., Flores-Álamo M., Escárcega-Bobadilla M. V. (2021). Supramolecular host-guest and fluorescence
studies on Ni-Salphen complex as a binding unit on edge oxidised graphene
oxide grafted nanomaterial. Supramol. Chem..

[19] Kleij A. W., Kuil M., Lutz M., Tooke D. M., Spek A. L., Kamer P. C. J., van Leeuwen P. W. N.
M., Reek J. N. H. (2006). Supramolecular
zinc­(II)­salphen motifs: Reversible dimerization and templated dimeric
structures. Inorg. Chim. Acta.

[20] Anselmo D., Gramage-Doria R., Besset T., Escárcega-Bobadilla M. V., Salassa G., Escudero-Adán E. C., Martínez
Belmonte M., Martin E., Reek J. N. H., Kleij A. W. (2013). Supramolecular
bulky phosphines comprising 1,3,5-triaza-7-phosphaadamantane and Zn­(salphen)­s:
structural features and application in hydrosilylation catalysis. Dalton Trans..

[21] Carbonaro L., Isola M., La Pegna P., Senatore L., Marchetti F. (1999). Spectrophotometric
Study of the Equilibria between Nickel­(II) Schiff-Base Complexes and
Alkaline Earth or Nickel­(II) Cations in Acetonitrile Solution. Inorg. Chem..

[22] Blondel B., Delarue F., Lopez M., Madeira-Mallet S., Alary F., Renaud C., Saski I. (2017). Investigation
of a
sterically hindered Pt­(II) complex to avoid aggregation-induced quenching:
Applications in deep red electroluminescent and electrical switching
devices. Synth. Met..

[23] Enríquez-Palacios E., Robledo-Patiño A. V., Zelada-Guillén G. A. (2024). Zn-Salphen
Acrylic Films Powered by Aggregation-Induced Enhanced Emission for
Sensing Applications. J. Fluoresc..

[24] Coucouvanis D., Rosa D., Pike J. (2003). Recognition
and transport of amphiphilic
molecules by a new class of inorganic ditopic receptors. The synthesis
of M-t Bu4-salphen-3n-cr-n complexes and their use (M = Mn,Fe, n =
6) in the transport of tryptophan and serotonin across bulk liquid
membranes. C. R. Chim..

[25] Chong J. H., Ardakani S. J., Smith K. J., MacLachlan M. J. (2009). Triptycene-Based
Metal Salphens—Exploiting Intrinsic Molecular Porosity for
Gas Storage. Chem. - Eur. J..

[26] Xu Z.-X., Huang Z.–T., Chen C.–F. (2009). Synthesis and structures of novel
enantiopure inherently chiral calix[4]­arene-derived salphen ligands
and their transition-metal complexes. Tetrahedron
Lett..

[27] Uhrmacher F., Elbert S. M., Rominger F., Mastalerz M. (2021). Synthesis
of Large [2 + 3] Salicylimine Cages with Embedded Metal-Salphen Units. Eur. J. Inorg. Chem..

[28] Battistin F., Vidal A., Cavigli P., Balducci G., Iengo E., Alezzio E. (2020). Orthogonal Coordination Chemistry of PTA toward Ru­(II)
and Zn­(II) (PTA = 1,3,5-Triaza-7-phosphaadamantane) for the Construction
of 1D and 2D Metal-Mediated Porphyrin Networks. Inorg. Chem..

[29] Phillips A. D., Gonsalvi L., Romerosa A., Vizza F., Peruzzini M. (2004). Coordination
chemistry of 1,3,5-triaza-7-phosphaadamantane (PTA): Transition metal
complexes and related catalytic, medicinal and photoluminescent applications. Coord. Chem. Rev..

[30] Bravo J., Bolaño S., Gonsalvi L., Peruzzini M. (2010). Coordination
chemistry of 1,3,5-triaza-7-phosphaadamantane (PTA) and derivatives.
Part II. The quest for tailored ligands, complexes and related applications. Coord. Chem. Rev..

[31] Bandaru J. S. S. M., Bhilare S., Schulzke C., Kapdi A. R. (2020). 1,3,5-Triaza-7-phosphaadamantane
(PTA) Derived Caged Phosphines for Palladium-Catalyzed Selective Functionalization
of Nucleosides and Heteroarenes. Chem. Rec..

[32] Hua J. K., Liu X., Chen J., Wei B., Wang H., Sun Y. (2021). Regioselective
Hydroxymethylation of Alkenes to Linear Alcohols with CO2/H2 Using
a Rh/Ru Dual Catalyst. ACS Sustainable Chem.
Eng..

[33] Lidrissi J. C., Romerosa A., Saoud M., Serrano-Ruiz M., Gonsalvi L., Perruzzini M. (2005). Stable, Water-Soluble Pta-Based Ru–Ag
Organometallic Polymers. Angew. Chem., Int.
Ed..

[34] Sierra-Martin J. B., Serrano-Ruiz M., García-Sakai V., Scalambra F., Romerosa A., Fernandez-Barbero A. (2018). Self-Organization
and Swelling of
Ruthenium-Metal Coordination Polymers with PTA (Metal = Ag, Au, Co). Polymers.

[35] Jaros J. S. W., Komarnicka U. K., Kyziol A., Pucelik B., Nesterov D. S., Krillov A. M., Smolénski P. (2022). Therapeutic
Potential of a Water-Soluble Silver-Diclofenac Coordination Polymer
on 3D Pancreatic Cancer Spheroids. J. Med. Chem..

[36] Kong J. M., Song H., Zhou J. (2018). Metal–Organophosphine
Framework-Derived
N,P-Codoped Carbon-Confined Cu3P Nanopaticlesfor Superb Na-Ion Storage. Adv. Energy Mater..

[37] Śliwa E. I., Nesterov D. S., Klak J., Jakimowicz P., Kirillov A. M., Smoleński P. (2018). Unique Copper–Organic Networks
Self-Assembled from 1,3,5-Triaza-7-Phosphaadamantane and Its Oxide:
Synthesis, Structural Features, and Magnetic and Catalytic Properties. Cryst. Growth Des..

[38] Gavara J. R., Pinto A., Donamaría R., Olmos M. E., López
de Luzuriaga J. M., Rodríguez L. (2017). Polarized Supramolecular Aggregates
Based on Luminescent Perhalogenated Gold Derivatives. Inorg. Chem..

[39] Online Tools for Supramolecular Chemistry Research and Analysis, OpenDataFit, P. Thordarson, CBNS & UNSW, Patron: Sir Fraser Stoddart. http://supramolecular.org.

[40] Thordarson P. (2011). Determining
association constants from titration experiments in supramolecular
chemistry. Chem. Soc. Rev..

[41] Hibbert D. B., Thordarson P. (2016). The death
of the Job plot, transparency, open science
and online tools, uncertainty estimation methods and other developments
in supramolecular chemistry data analysis. Chem.
Commun..

[42] Li X., Li J., Kang F. (2019). Enhanced electrochemical
performance of salen-type
transition metal polymer with electron-donating substituents. Ionics.

[43] Deng F., Li X., Ding F., Niu B., Li J. (2018). Pseudocapacitive Energy
Storage in Schiff Base Polymer with Salphen-Type Ligands. J. Phys. Chem. C.

[44] Matienzo L. J., Grim S. O. (1973). X-ray photoelectron spectra of square
planar and octahedral
nickel (II) complexes. Inorg. Nucl. Chem. Letters.

[45] Korusenko P. M., Petrova O. V., Vereshchagin A. A., Katin K. P., Levin O. V., Nekipelov S. V., Sivkov D. V., Sivkov V. N., Vinogradov A. S. (2023). A Comparative
XPS, UV PES, NEXAFS, and DFT Study of the Electronic Structure of
the Salen Ligand in the H2­(Salen) Molecule and the [Ni­(Salen)] Complex. Int. J. Mol. Sci..

[46] Al-Gaashani R., Najjar A., Zakaria Y., Mansour S., Atieh M. A. (2019). XPS and
structural studies of high quality graphene oxide and reduced graphene
oxide prepared by different chemical oxidation methods. Ceram. Int..

[47] Lopez A., Amatori S., Olivieri E., Venditti I., Iucci G., Meneghini C., Bertelà F., Del Bello F., Quaglia W., Pellei M., Santini C., Battocchio C. (2024). Cu­(I) Coordination
Compounds Conjugated to Au Nanorods for Future Applications in Drug
Delivery: Insights in Molecular, Electronic and Cu Local Structure
in Solid and Liquid Phase. ChemPhysChem.

[48] Kong M., Song H., Zhou J. (2018). Metal–Organophosphine
Framework-Derived
N,P-Codoped Carbon-Confined Cu3P Nanopaticles for Superb Na-Ion Storage. Adv. Energy Mater..

[49] Mahmoud A. G., Librando I. L., Paul A., Carabineiro S. A. C., Ferraria A. M., Botelho do Rego A. M., Guedes da Silva M. F. C., Geraldes C. F. G. C., Pombeiro A. J. L. (2023). Novel organotin-PTA
complexes supported on mesoporous carbon materials as recyclable catalysts
for solvent-free cyanosilylation of aldehydes. Catal. Today.

[50] López-Vinasco A. M., Favier I., Pradel C., Huerta L., Guerrero-Ríos I., Teuma E., Gómez M., Martin E. (2014). Unexpected bond activations
promoted by palladium nanoparticles. Dalton
Trans..

[51] Kumar G., Blackburn J. R., Albridge R. G., Moddeman W. E., Jones M. M. (1972). Photoelectron
Spectroscopy of Coordination Compounds. II. Palladium Complexes. Inorg. Chem..

[52] Kehrer M., Duchoslav J., Hinterreiter A., Cobet M., Mehic A., Stehrer T., Stifter D. (2018). XPS investigation on the reactivity
of surface imine groups with TFAA. Plasma Processes
Polym..

[53] Peng B., Xu Y., Liu K., Wang X., Mulder F. M. (2017). High-Performance
and Low-Cost Sodium-Ion Anode Based on a Facile Black Phosphorus-Carbon
Nanocomposite. ChemElectroChem.

[54] Zhu J., Jiang S. P., Wang R., Shi K., Shen P. K. (2014). One-pot
synthesis of a nitrogen and phosphorus-dual-doped carbon nanotube
array as a highly effective electrocatalyst for the oxygen reduction
reaction. J. Mater. Chem. A.

[55] Li J.-S., Wang Y., Liu C.-H., Liu S.-L., Wang Y.-G., Dong L.-Z., Dai Z.-H., Li Y.-F., Lan Y.-Q. (2016). Coupled
molybdenum carbide and reduced graphene oxide electrocatalysts for
efficient hydrogen evolution. Nat. Commun..

[56] Hou S., Bai L., Lu D., Duan H. (2023). Interfacial Colloidal
Self-Assembly
for Functional Materials. Acc. Chem. Res..

[57] Wang C., Yao Y., Li J., Yamauchi Y. (2022). Metal–Organic Frameworks:
A Robust Platform for Creating Nanoarchitectured Carbon Materials. Acc. Mater. Res..

[58] Zhang T., Zhang G., Chen L. (2022). 2D Conjugated
Covalent Organic Frameworks:
Defined Synthesis and Tailor-Made Functions. Acc. Chem. Res..

[59] Jaryal V. B., Villa A., Gupta N. (2023). Metal-Free Carbon-Based
Nanomaterials:
Insights from Synthesis to Applications in SustainableCatalysis. ACS Sustainable Chem. Eng..

[60] Ma R., Xue Y., Ma Q., Chen Y., Yuan S., Fan J. (2022). Recent Advances
in Carbon-Based Materials for Adsorptive and PhotocatalyticAntibiotic
Removal. Nanomaterials.

[61] Muschi M., Serre C. (2019). Progress and challenges
of graphene oxide/metal-organic composites. Coord. Chem. Rev..

[62] Mohammed A. K., Usgaonkar S., Kanheerampockil F., Karak S., Halder A., Tharkar M., Addicoat M., Ajithkumar T. G., Banerjee R. (2020). Connecting Microscopic Structures,
Mesoscale Assemblies,
and Macroscopic Architectures in 3D-Printed Hierarchical Porous Covalent
Organic Framework Foams. J. Am. Chem. Soc..

[63] Khan I., Liu W., Zada A., Raziq F., Ali S., Shah M. I. A., Ateeq M., Khan M., Alei D., Fazil P., Khan W., Khan J. A., Cai Y., Jin W., Yun S., Yang L. (2024). Recent progress in emerging materials and hybrid nanocomposites
for peroxymonosulfate and peroxydisulfate activation towards solar
light-driven photocatalytic degradation of emerging pollutants. Coord. Chem. Rev..

[64] Vardali S. C., Manoussi N., Barczak M., Giannakoudakis D. A. (2020). Novel Approaches
Utilizing Metal-Organic Framework Composites for the Extraction of
Organic Compounds and Metal Traces from Fish and Seafood. Molecules.

[65] Shah I. A., Bilal M., Almanassra I. W., Ihsanullah I. (2024). A comprehensive
review of graphene oxide-based membranes for efficient dye removal
from water sources. Sep. Purif. Technol..

[66] Ayati A., Tanhaei B., Beiki H., Krivoshapkin P., Krivoshapkina E., Tracey C. (2023). Insight into the adsorptive removal
of ibuprofen using porous carbonaceous materials: A review. Chemosphere.

[67] Al-Ghouti M. A., Ashfaq M. Y., Khan M., Disi Z. A., Da’na D. A., Shoshaa R. (2023). State-of-the-art adsorption
and adsorptive filtration
based technologies for the removal of trace elements: A critical review. Sci. Total Environ..

[68] Choi J., Wagner P., Jalili R., Kim J., MacFarlane D. R., Wallace G. G., Officer D. L. (2018). A Porphyrin/Graphene
Framework: A
Highly Efficient and Robust Electrocatalyst for Carbon Dioxide Reduction. Adv. Energy Mater..

[69] Bae H., Park M., Jang B., Kang Y., Park J., Lee H., Chung H., Chung C., Hong S., Kwon Y., Yakobson B. I., Lee H. (2016). High-throughput screening of metal-porphyrin-like
graphenes for selective capture of carbon dioxide. Sci. Rep..

[70] Kämpfe A., Kroke E., Wagler J. (2009). Hypercoordinate
Silicon Complexes
of (O,N,N′ vs. O,N,O′) Schiff Base Type N-(2-Carbamidophenyl)­imines:
Examples of Exclusively O-Silylated Carbamides. Eur. J. Inorg. Chem..

[71] Alyea E. C., Ferguson G., Kannan S. (1998). Some water-soluble organometallic
complexes of group 10 transition metal (II) ions with 1,3,5-triaza-7-phosphaadamantane
(TPA). Syntheses, characterization and reactivity. The crystal and
molecular structure of [Ni­(CN)_2_(TPA)_3_]·4.3H_2_O. Polyhedron.

[72] Zelada-Guillén G. A., Hernández-Pacheco P., Romero-Ávila M., Cañas-Alonso R.
C., Flores-Álamo M., Escárcega-Bobadilla M. V. (2020). Acrylic Polymers Containing a Nickel
Salphen Complex: An Approach to Supramolecular and Macromolecular
Systems. ChemPlusChem.

[73] Hernández-Pacheco P., Zelada-Guillén G. A., Romero-Ávila M., Cañas-Alonso R.
C., Flores-Álamo M., Escárcega-Bobadilla M. V. (2023). Enhanced Host-Guest Association and
Fluorescence in Copolymers from Copper Salphen Complexes by Supramolecular
Internalization of Anions. ChemPlusChem.

[74] Miller, J. N. ; Miller, J. C. Statistics and Chemometrics for Analytical Chemistry; Pearson: Essex, UK, 2010; pp 110–153.

[75] Somvanshi, P. R. ; Venkatesh, K. V. Encyclopedia of Systems Biology, Hill Equation; Springer: New York, NY, 2013; pp 892–895.

