# Supplementary material for: Supramolecular Frameworks from Graphene Edge-Grafted with Ni-Salphen Complexes and Pd-PTA Linkers
Source: ACS Omega. 2025 Aug 9;10(32):36382–95. doi: 10.1021/acsomega.5c04731 (PMC12368682; doi:10.1021/acsomega.5c04731)
Supplement: Supplementary file 1 [file ao5c04731_si_001.pdf]

Supporting information for

SUPRAMOLECULAR FRAMEWORKS FROM GRAPHENE EDGE-GRAFTED WITH NI-SALPHEN COMPLEXES AND Pd-PTA LINKERS

*Paulina Hernández-Pacheco, Gustavo A. Zelada-Guillén and Martha V. Escárcega-Bobadilla**

Synthesis and characterization of Ni-Salphen complexes

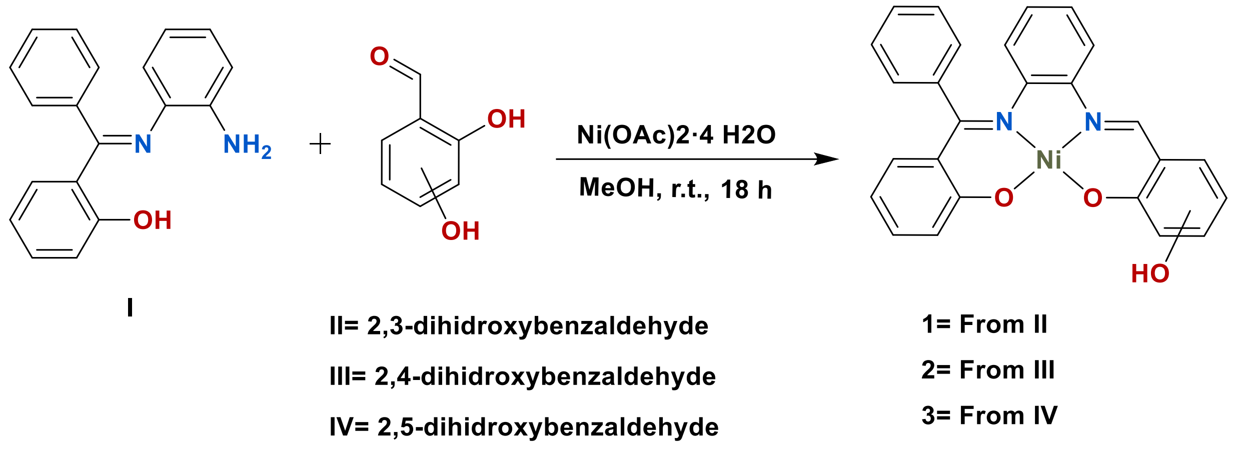

Scheme S1. Synthesis of all Ni-Salphen complexes.

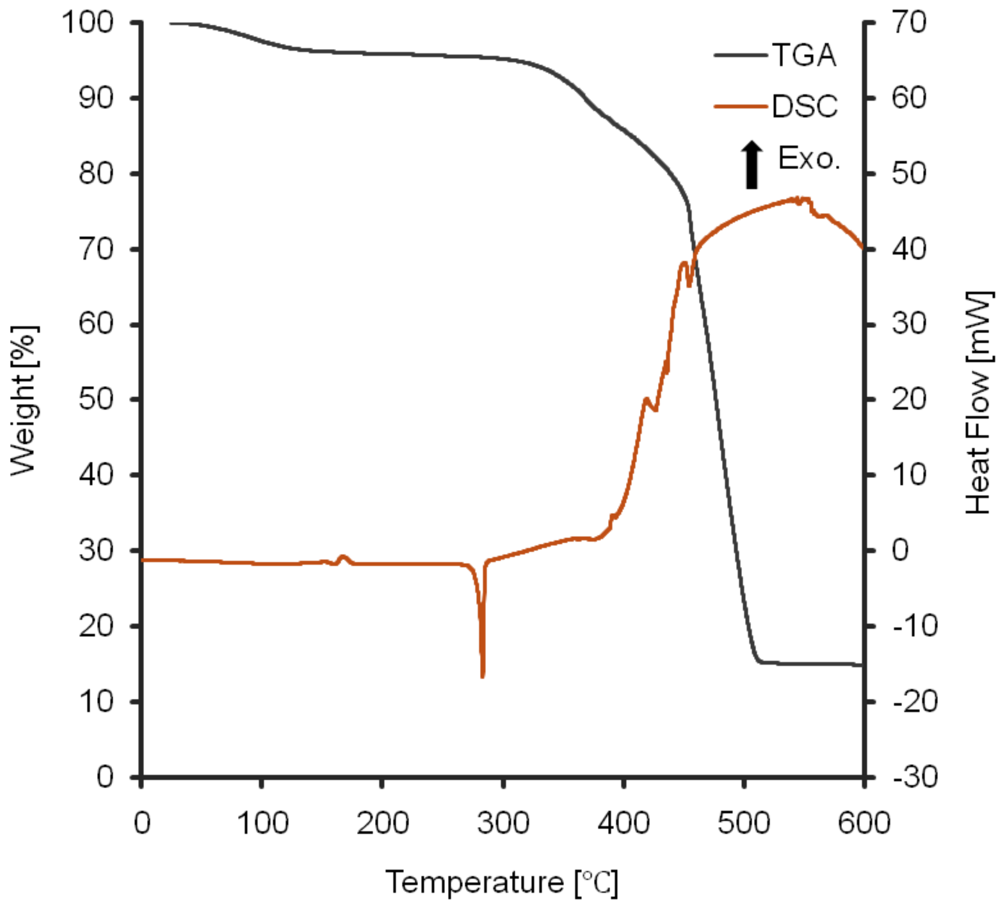

Figure S1. TGA (black) and DSC (orange) curves of complex **1** from 0 to 600 °C.

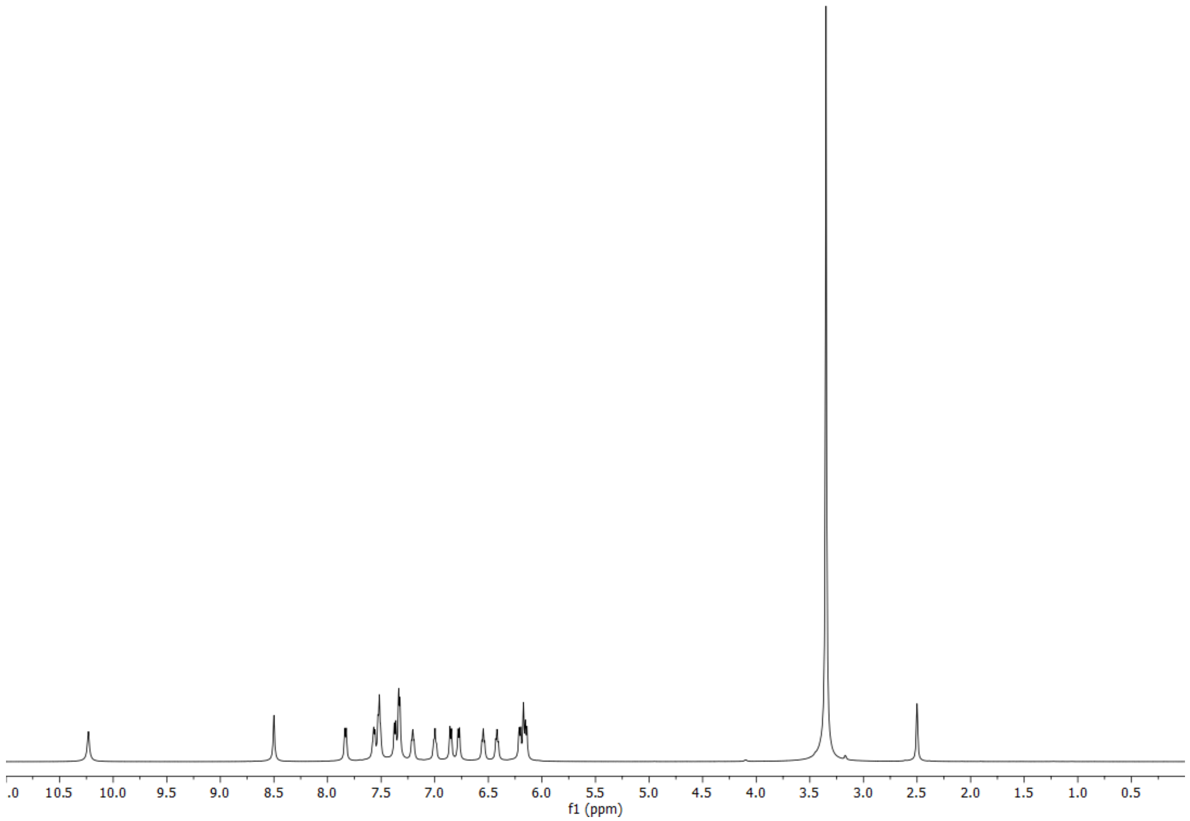

Figure S2.^{1}H NMR spectrum of complex **2**.(600 MHz, DMSO-d_6)

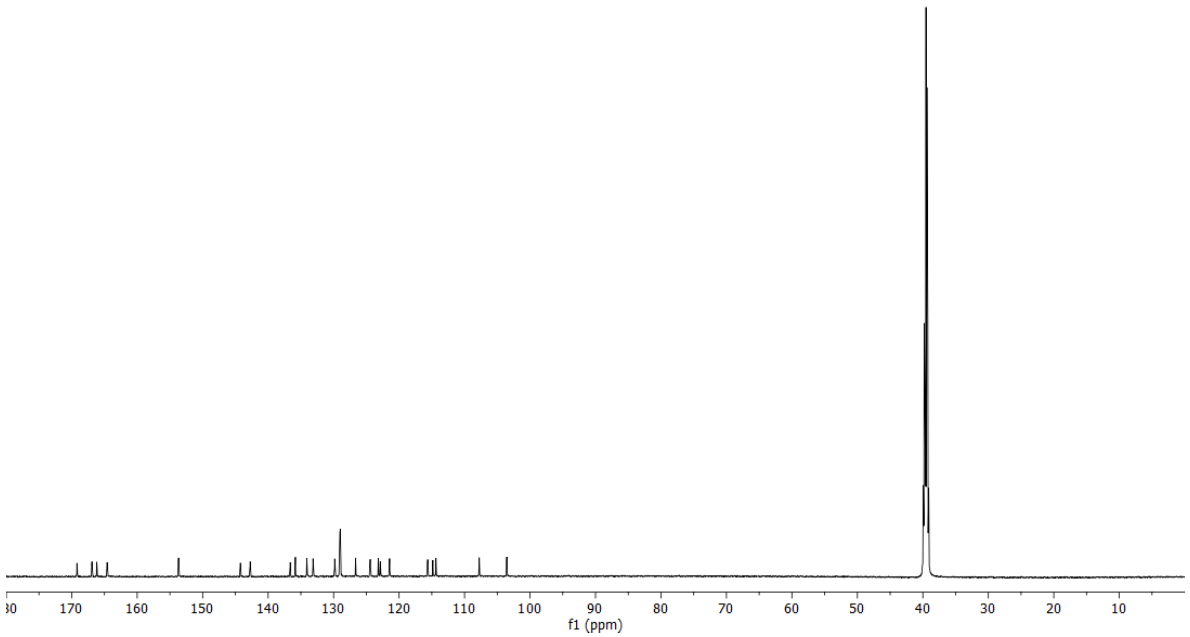

Figure S3.$^{13}C\{^{1}H\}$NMR spectrum of complex **2**.(100 MHZ, DMSO-*d*$_6$).

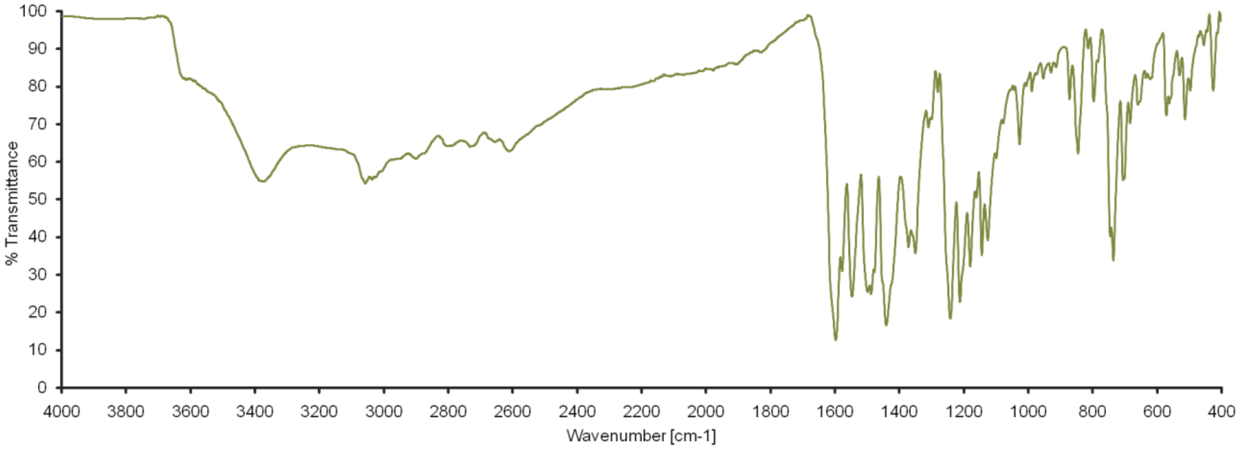

Figure S4. FT-IR spectrum of complex **2**.

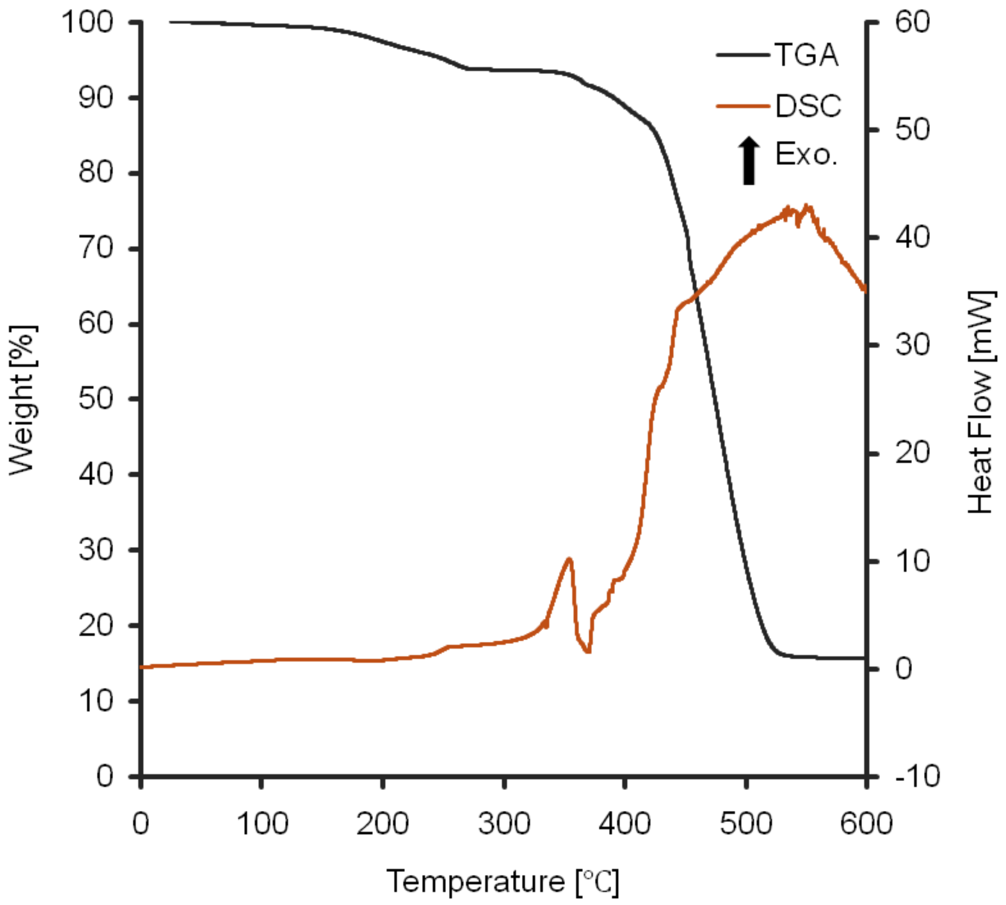

Figure S5. TGA (black) and DSC (orange) curves of complex **2** from 0 to 600 °C.

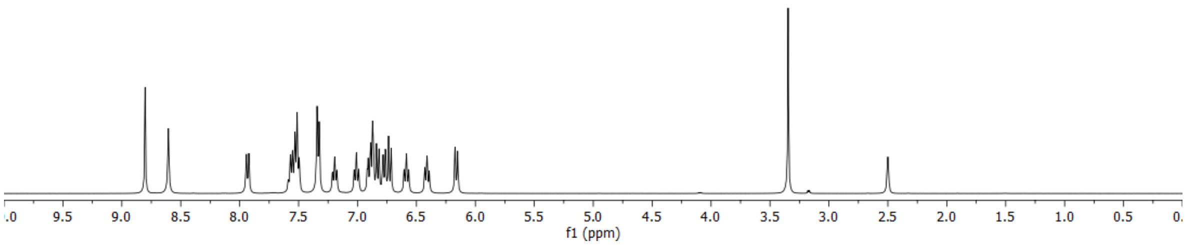

Figure S6. ^{1}H NMR spectrum of complex **3**.(400 MHz, DMSO-d_6).

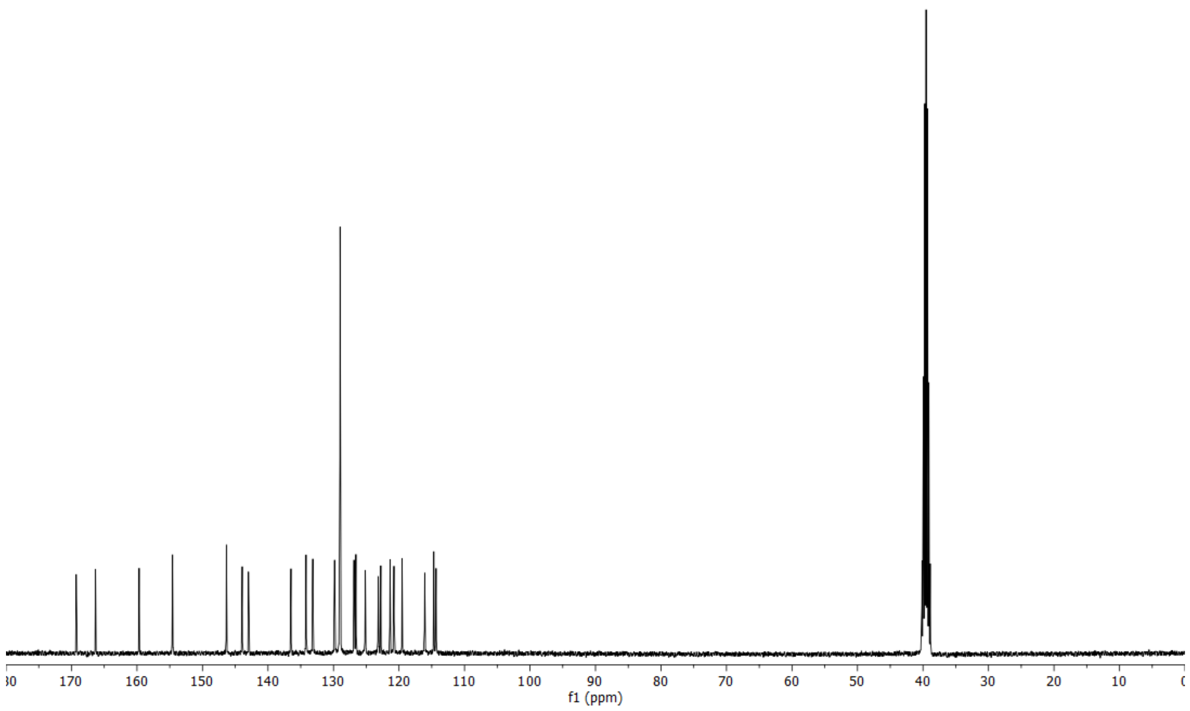

Figure S7. $^{13}C\{^{1}H\}$NMR spectrum of complex **3**. (100 MHz, DMSO-d_6).

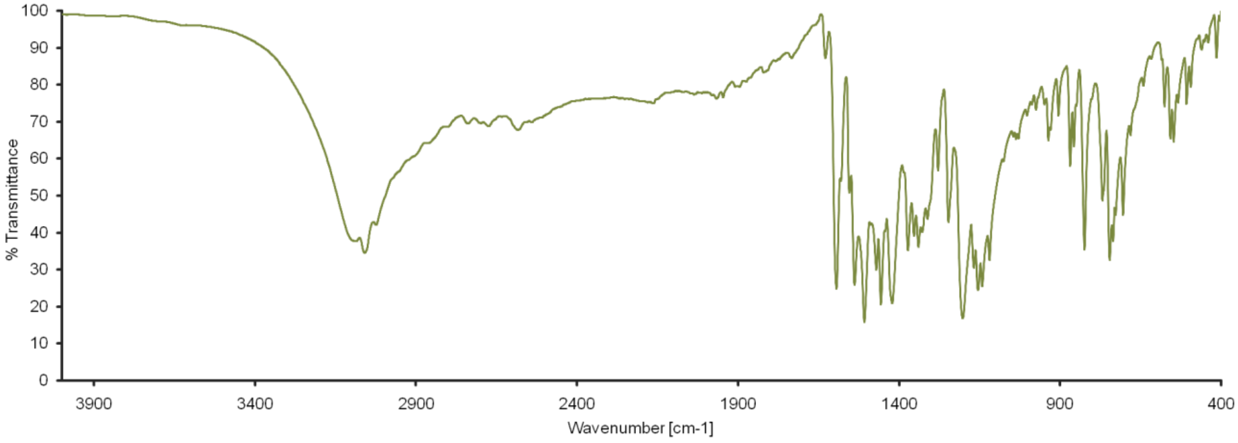

Figure S8. FT-IR spectrum of complex **3**.

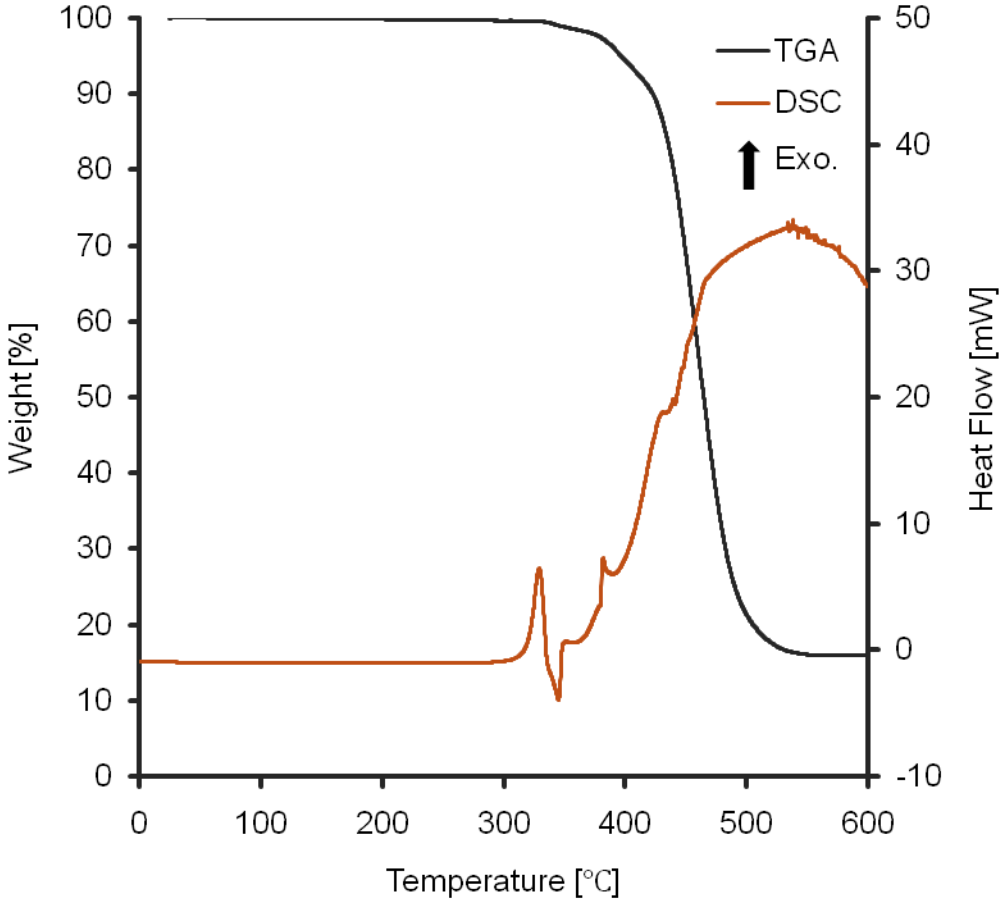

Figure S9. TGA (black) and DSC (orange) curves of complex **3** from 0 to 600 °C.

Synthesis and characterization of EOGO-*g*-[Ni-Salphen] grafted nanomaterials

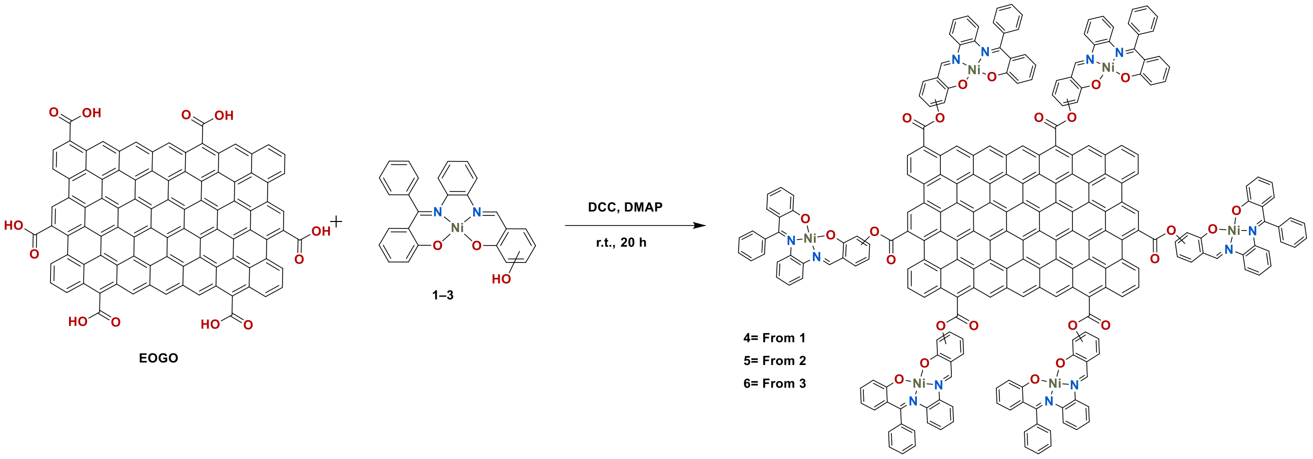

Scheme S2. Synthesis of all grafted nanomaterials using a representation of possible grafting placement.

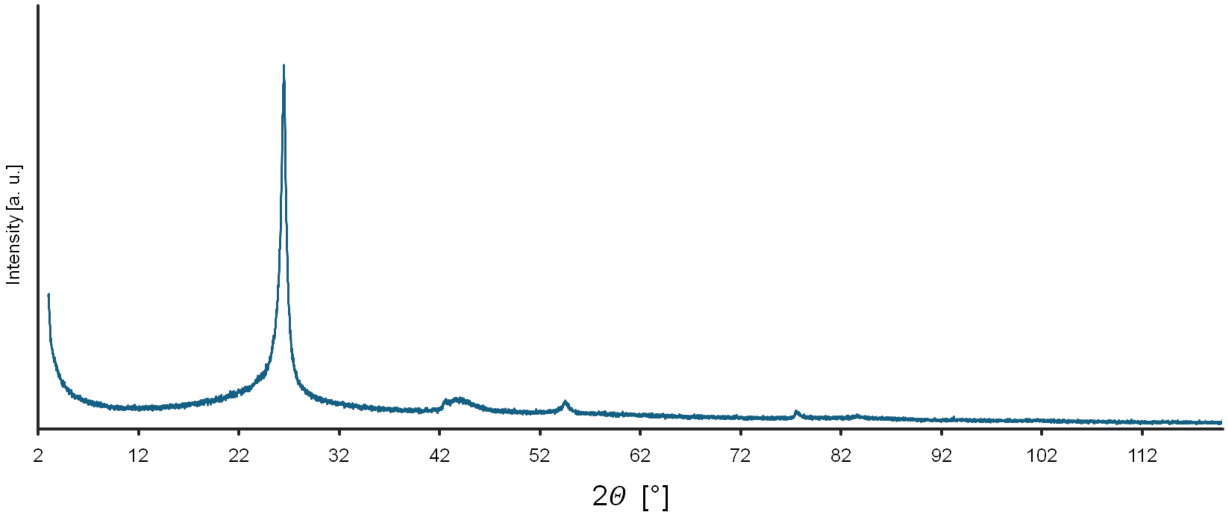

Figure S10. PXRD pattern of grafted nanomaterial **4**.

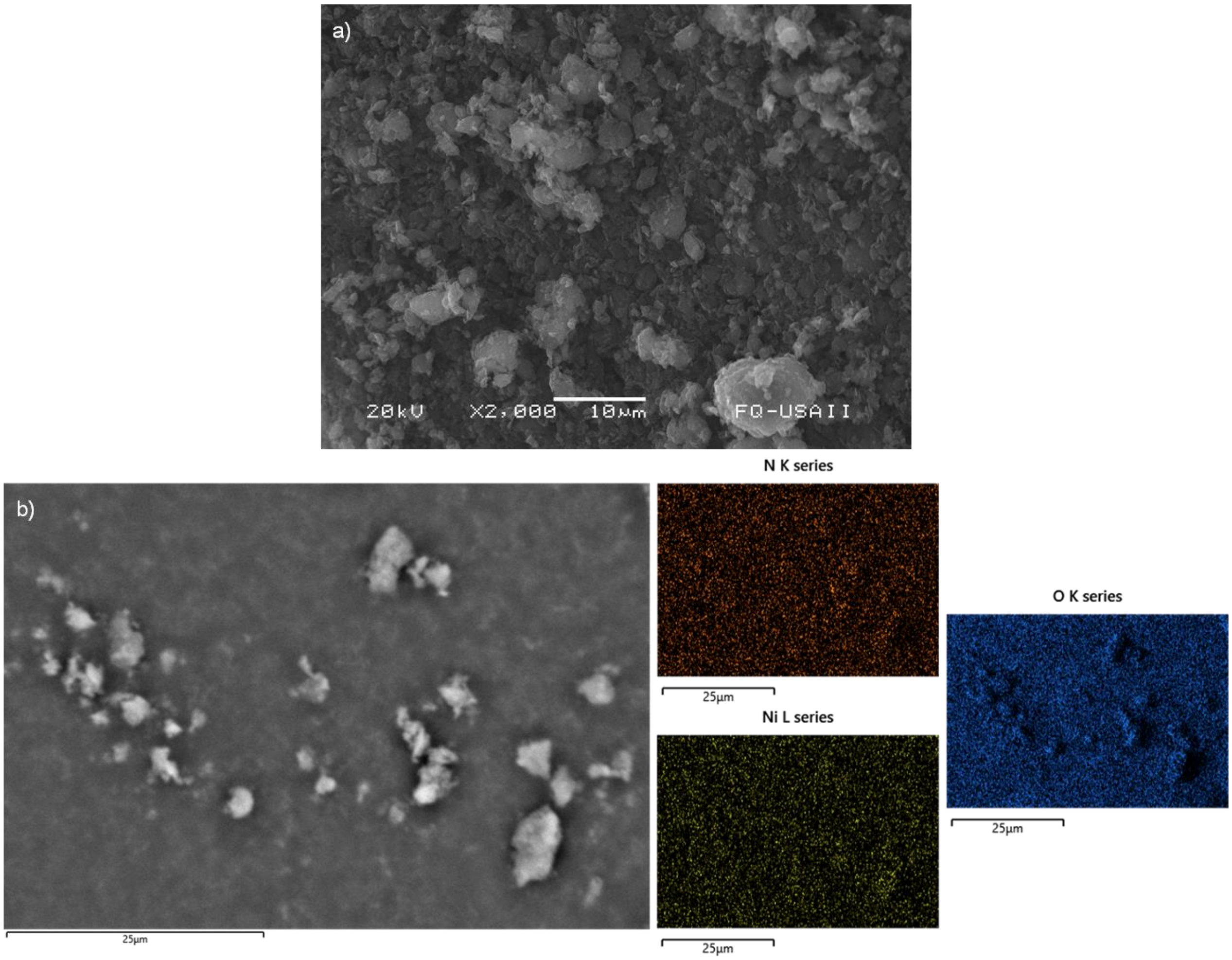

Figure S11. SEM micrograph (a) and elemental EDS mapping for O, N and Ni (b) of grafted nanomaterial **4**.

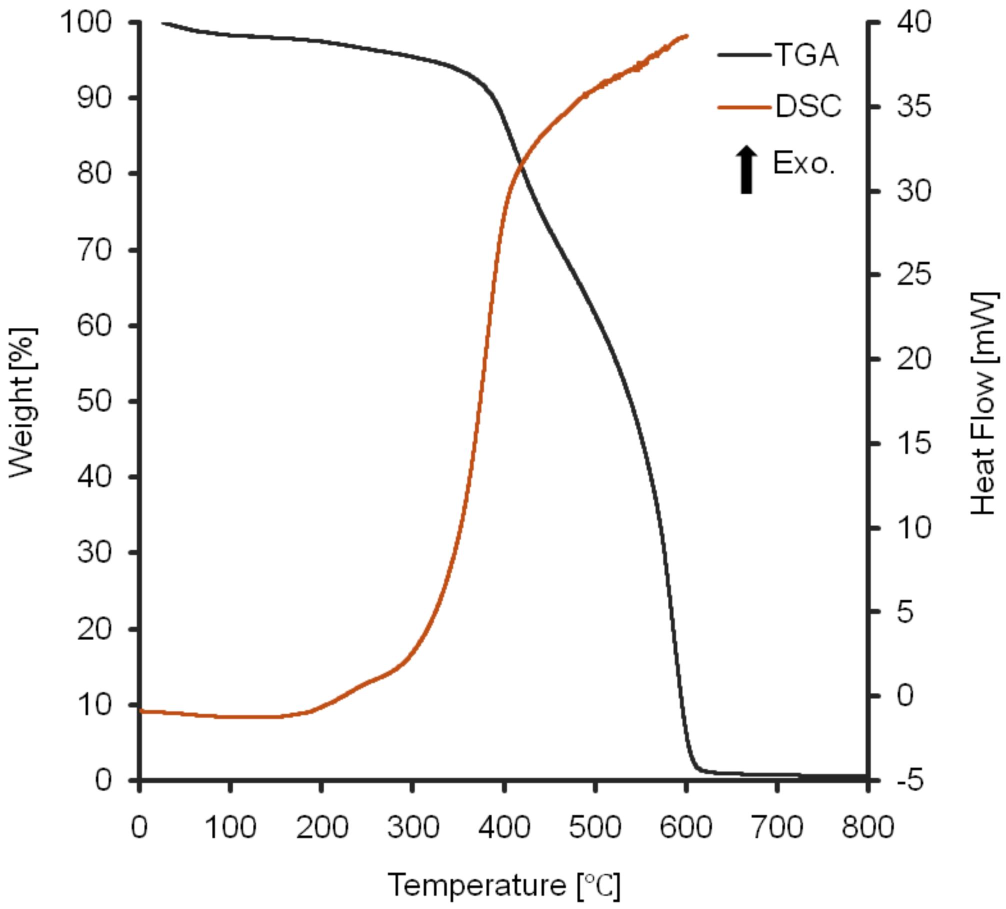

Figure S12. TGA (black) and DSC (orange) curves of grafted nanomaterial **4**, from 0 to 600 °C for DSC analysis and 800 °C for TGA.

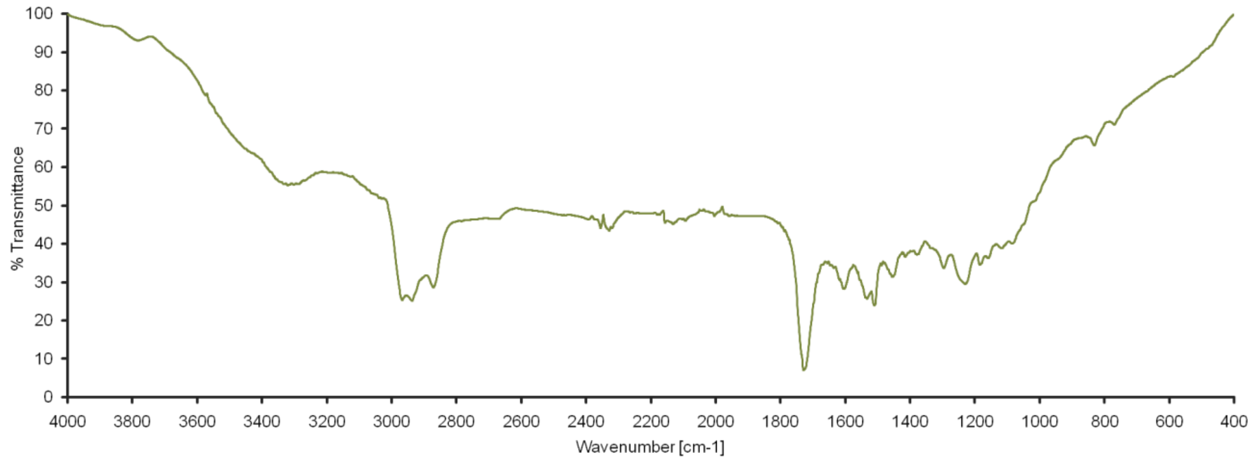

Figure S13. FT-IR spectra of grafted nanomaterial **5**.

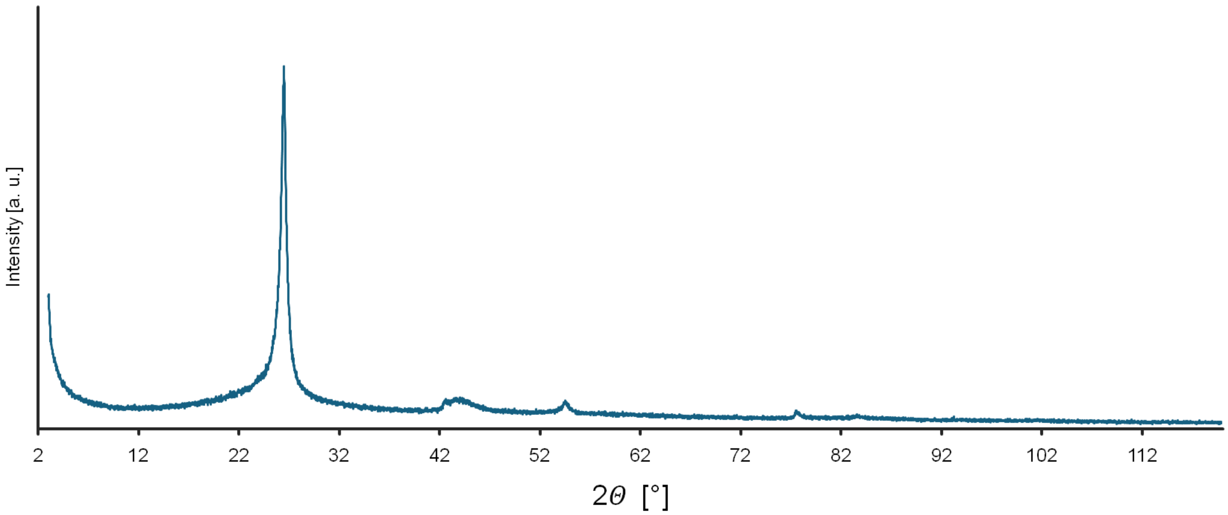

Figure S14. PXRD pattern of grafted nanomaterial **5**.

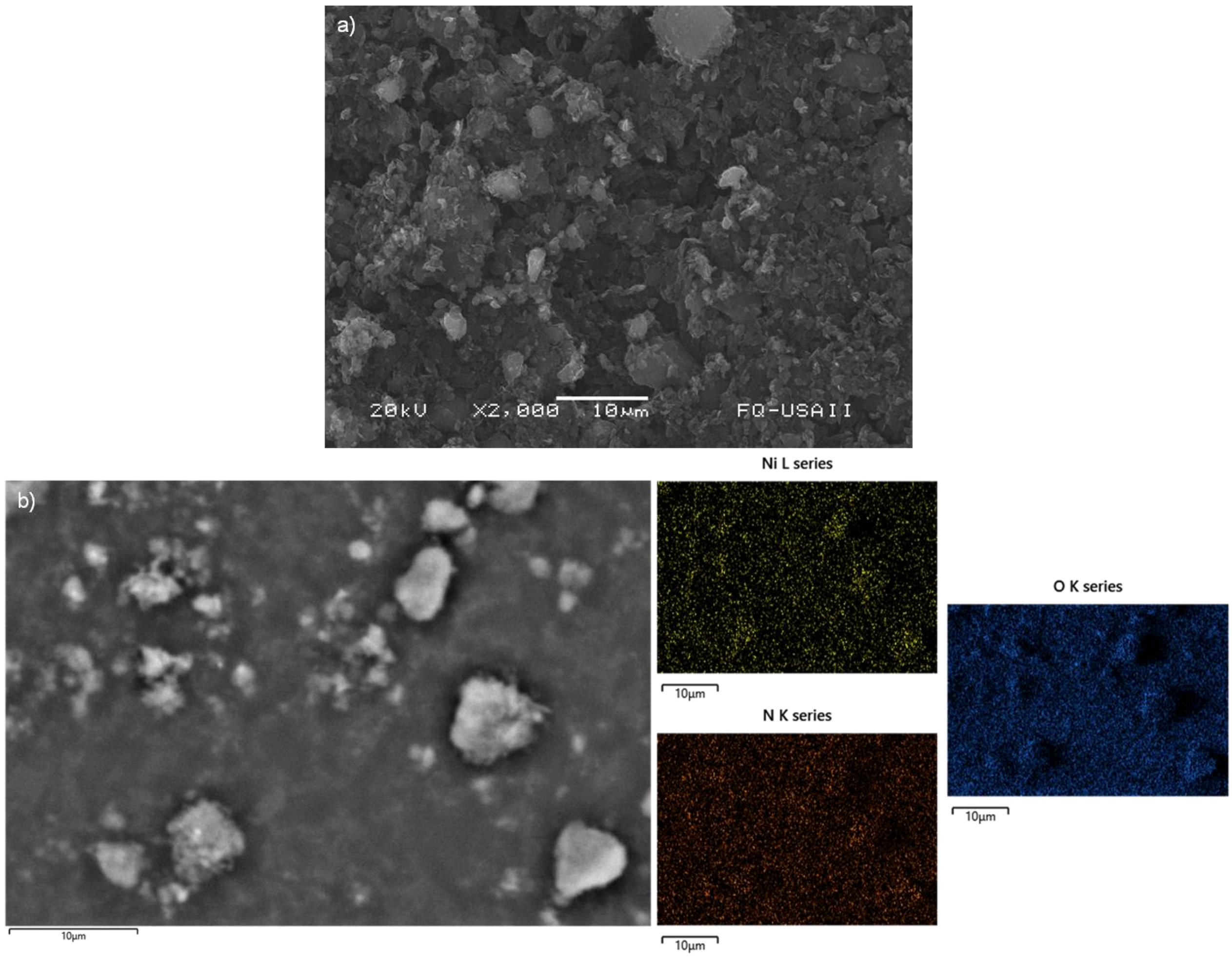

Figure S15.SEM micrograph (a) and elemental EDS mapping for O, N and Ni (b) of grafted nanomaterial **5**.

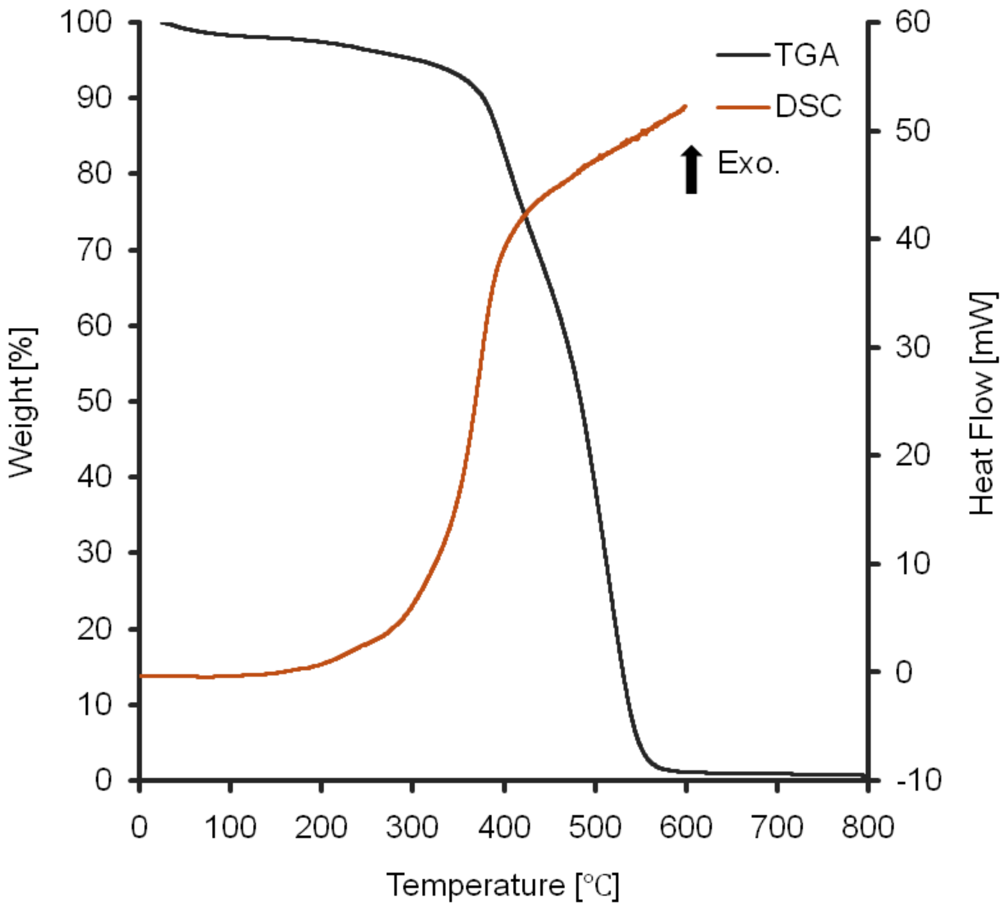

Figure S16. TGA (black) and DSC (orange) curves of grafted nanomaterial **5**, from 0 to 600 °C for DSC analysis and 800 °C for TGA.

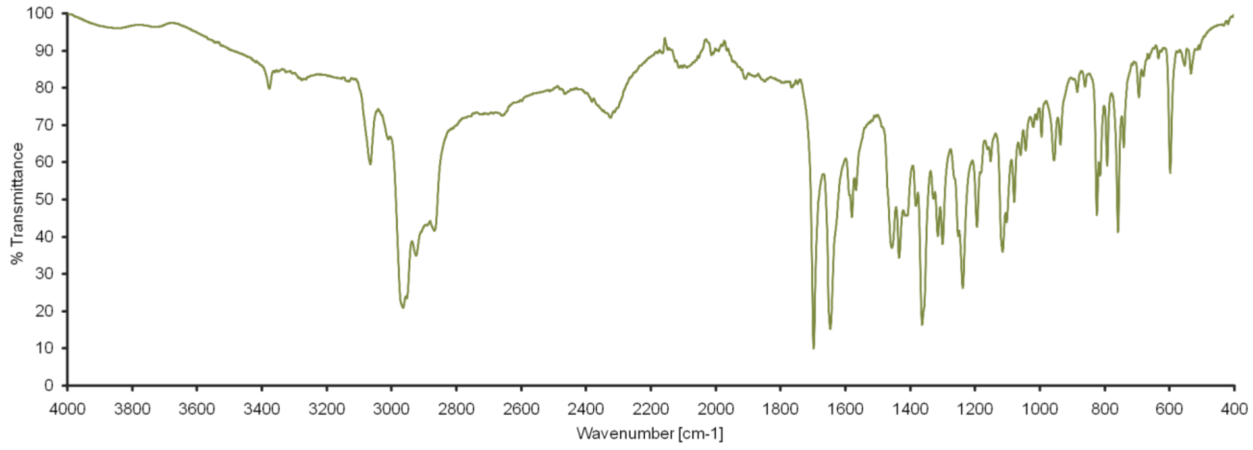

Figure S17. FT-IR spectra of grafted nanomaterial **6**.

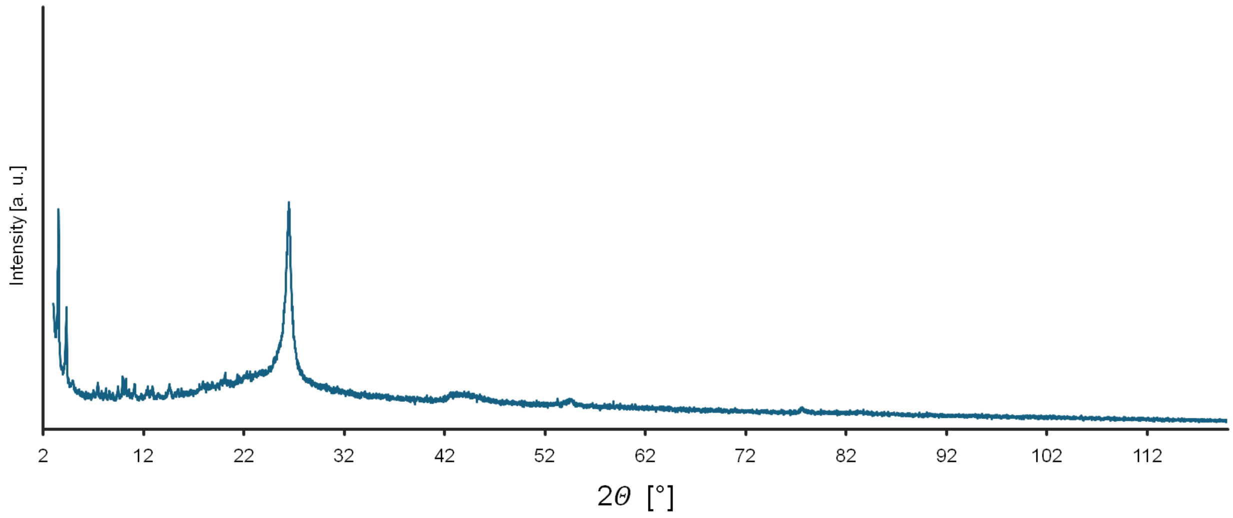

Figure S18. PXRD pattern of grafted nanomaterial **6.**

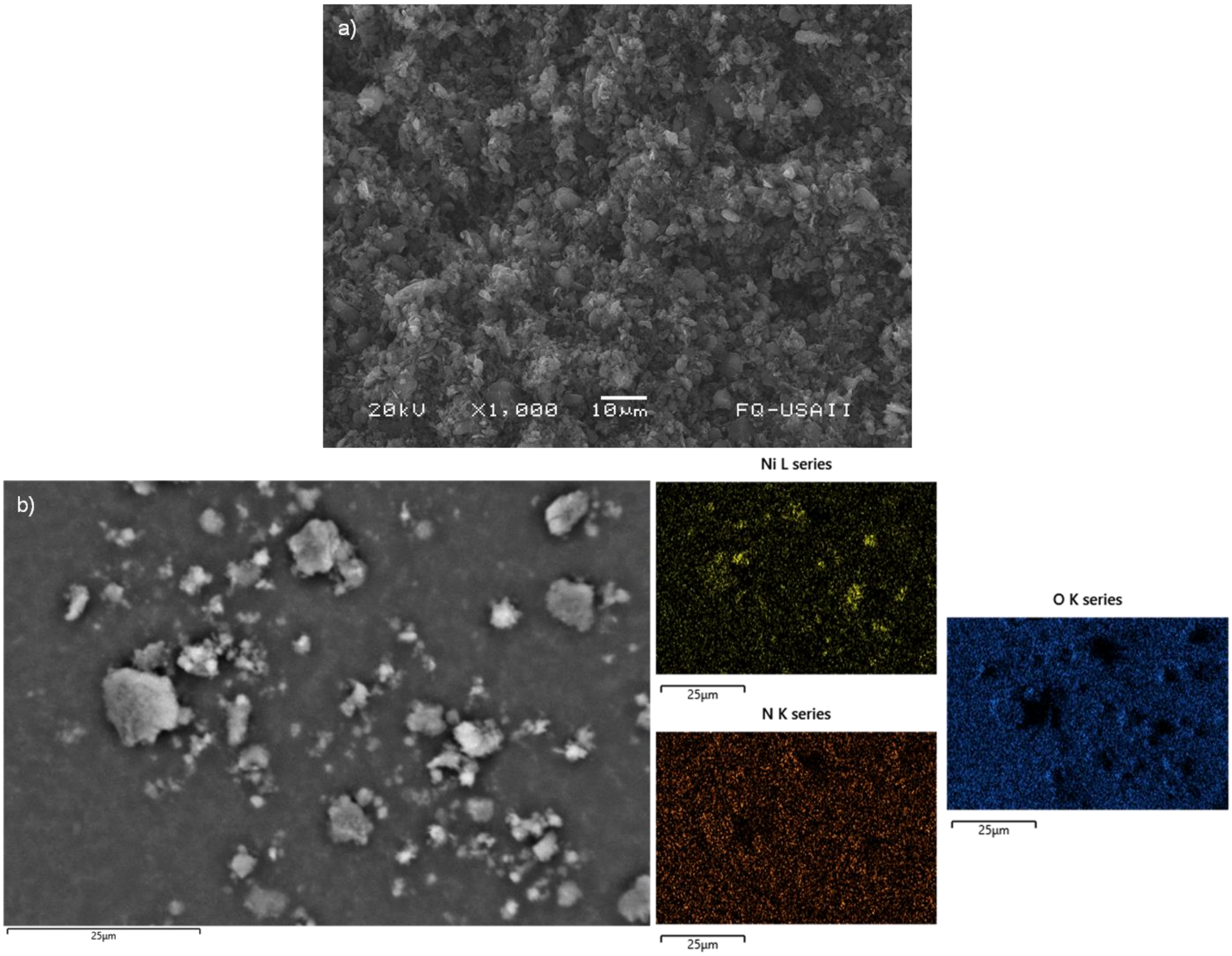

Figure S19. SEM micrograph (a) and elemental EDS mapping for O, N and Ni (b) of grafted nanomaterial **6**.

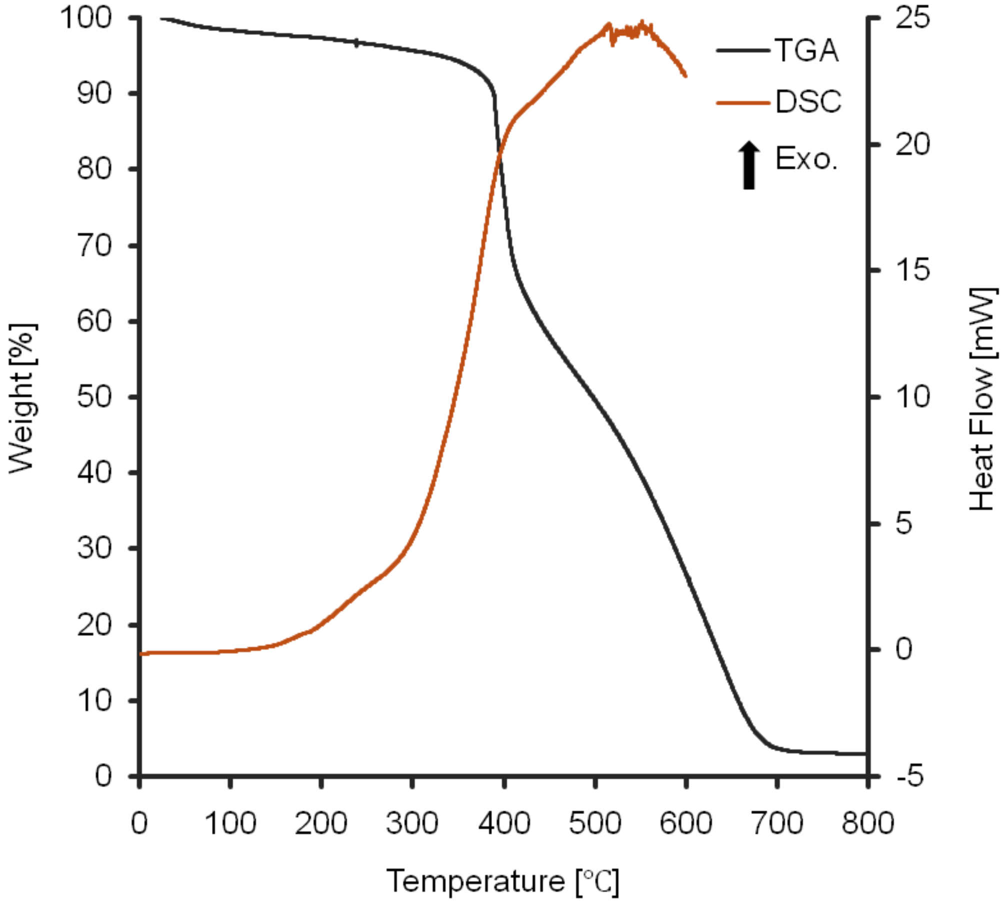

Figure S20. TGA (black) and DSC (orange) curves of grafted nanomaterial **6**, from 0 to 600 °C for DSC analysis and 800 °C for TGA.

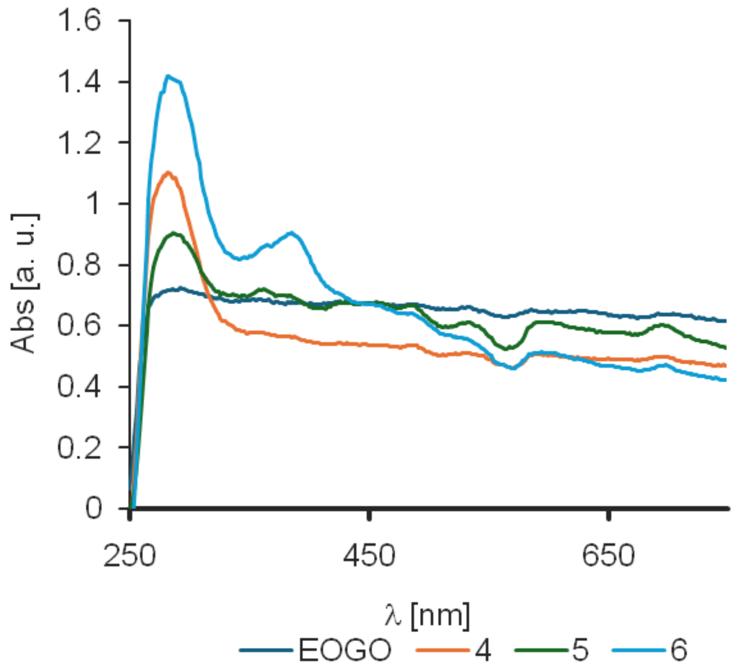

Figure S21. UV-Vis absorption spectra of EOGO (dark blue), complexes **4-6** (orange, green and light blue respectively).

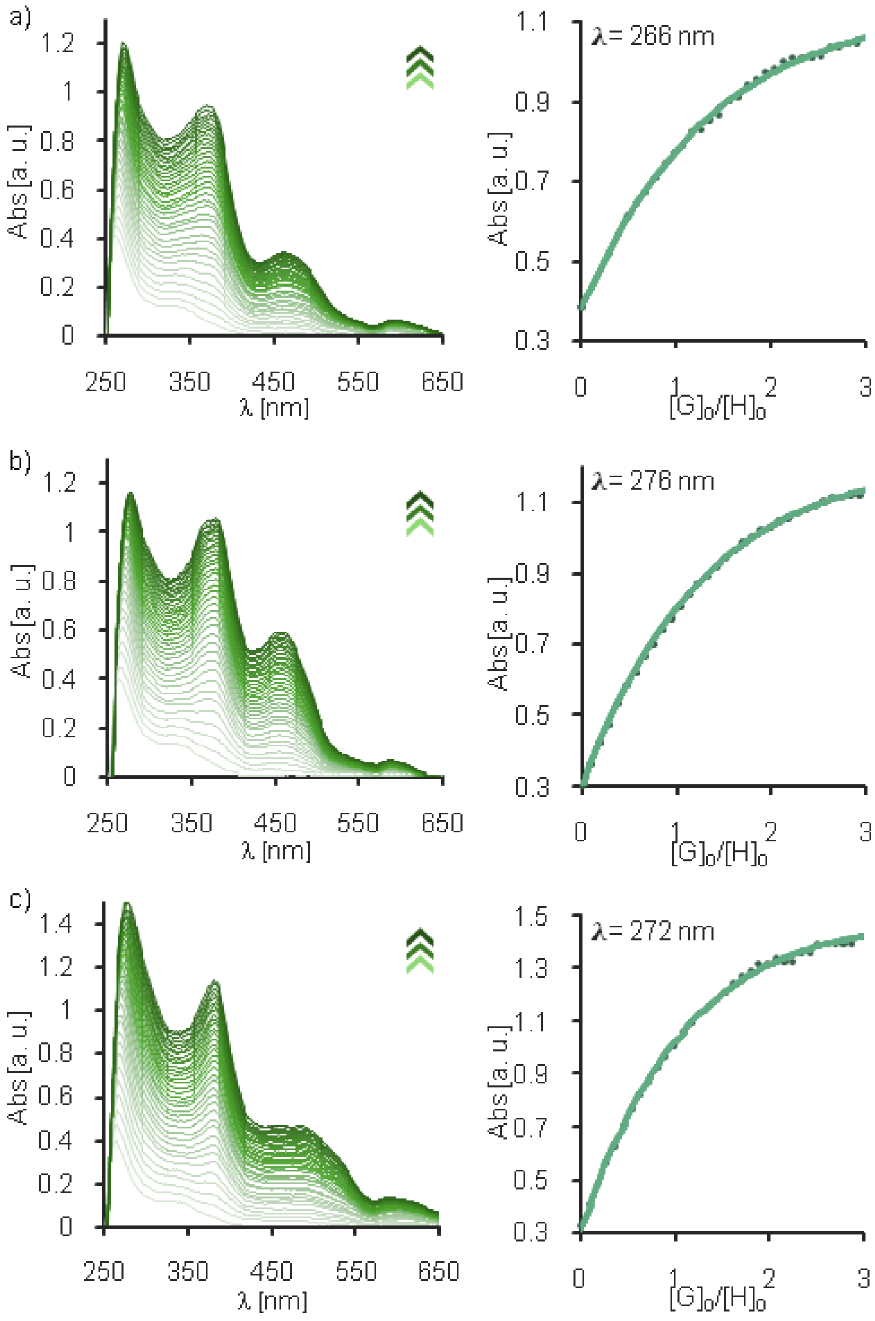

Figure S22. UV-Vis spectra from supramolecular titrations of Pd-PTA with each of the Ni-Salphen molecules **1**-**3** (left column), and example of a binding isotherm obtained for each system (right column); markers represent titration data corrected from dilution and green line is from data fitting using Bindfit; the abscissas at the isotherms in the right column represent [Ni-Salphen]/[Pd-PTA] ratio expressed in equivalents. (a) Pd-PTA vs. **1**. (b) Pd-PTA vs. **2**. (c) Pd-PTA vs. **3**.

Synthesis and characterization of [Pd-PTA]:[Ni-Salphen] supramolecules

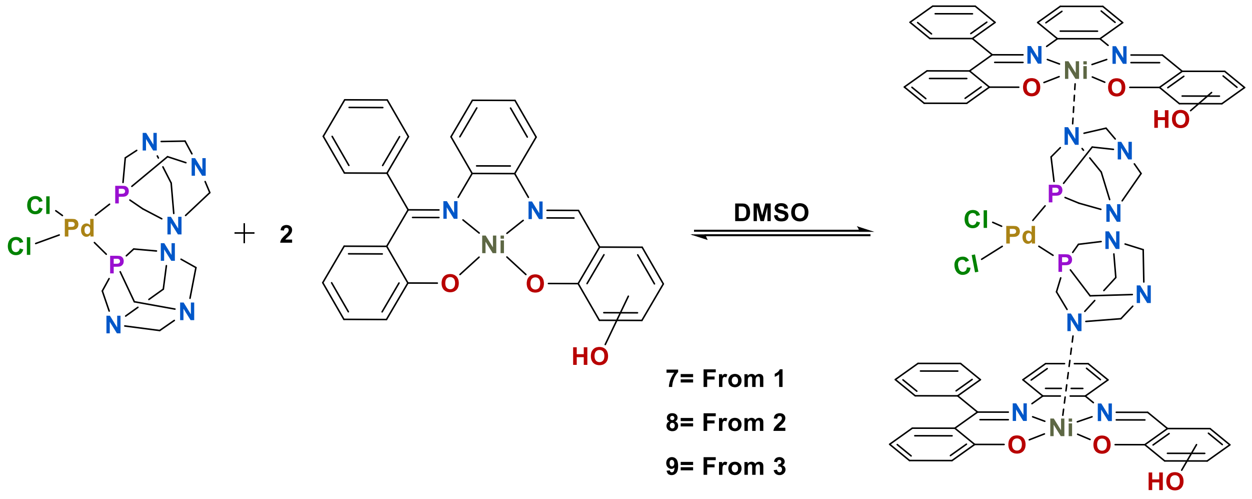

Scheme S3. Synthesis of [Pd-PTA]:[Ni-Salphen] supramolecules.

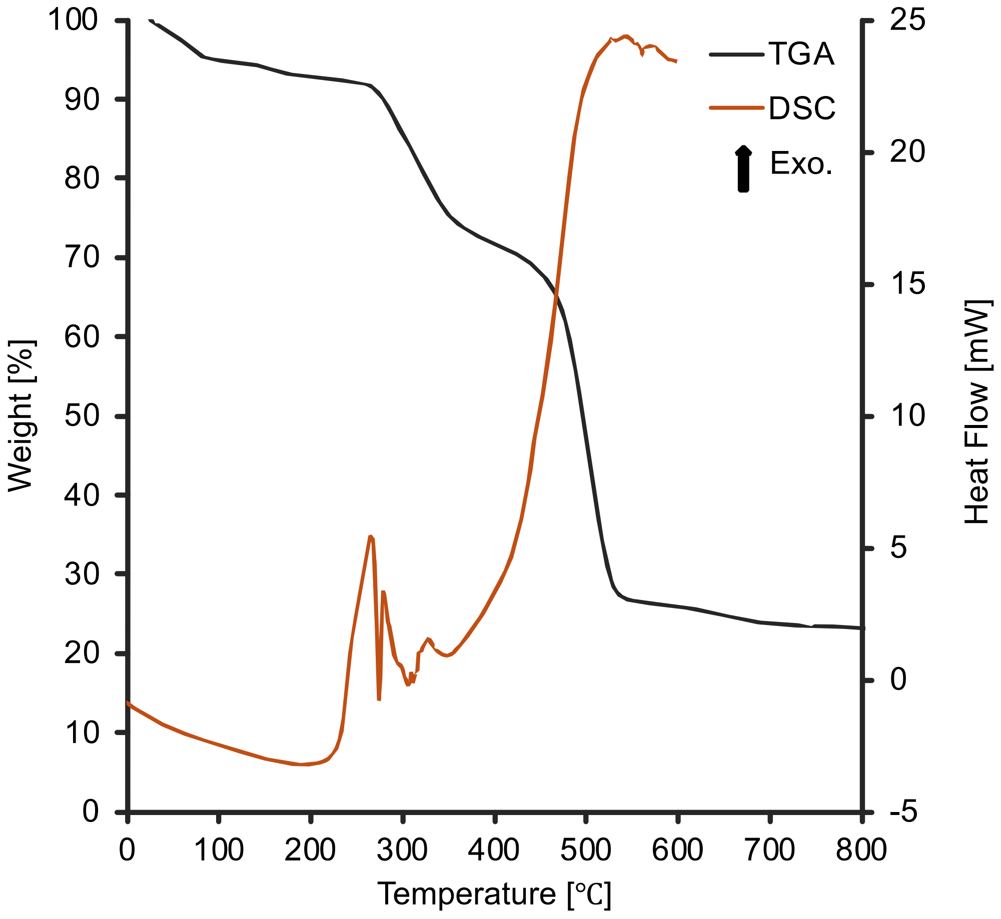

Figure S23. TGA (black) and DSC (orange) curves of supramolecule **7**, from 0 to 600 °C for DSC analysis and 800 °C for TGA.

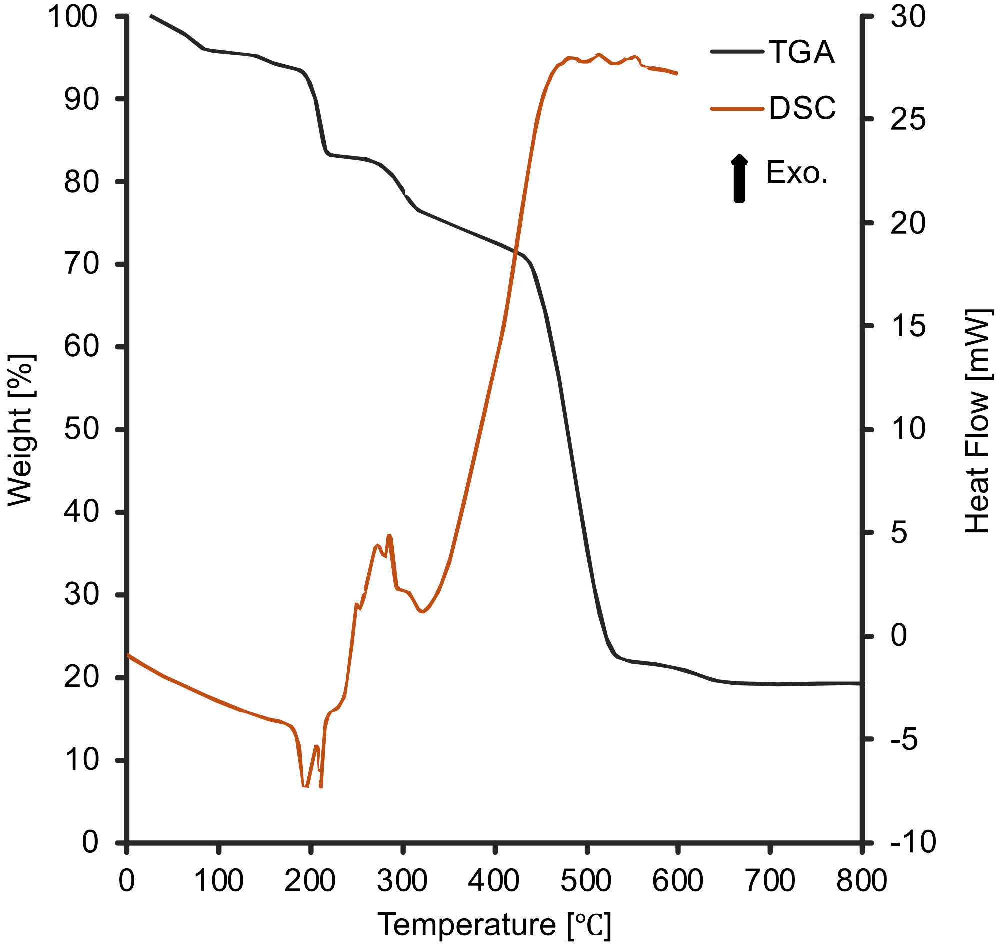

Figure S24. TGA (black) and DSC (orange) curves of supramolecule **8**, from 0 to 600 °C for DSC analysis and 800 °C for TGA.

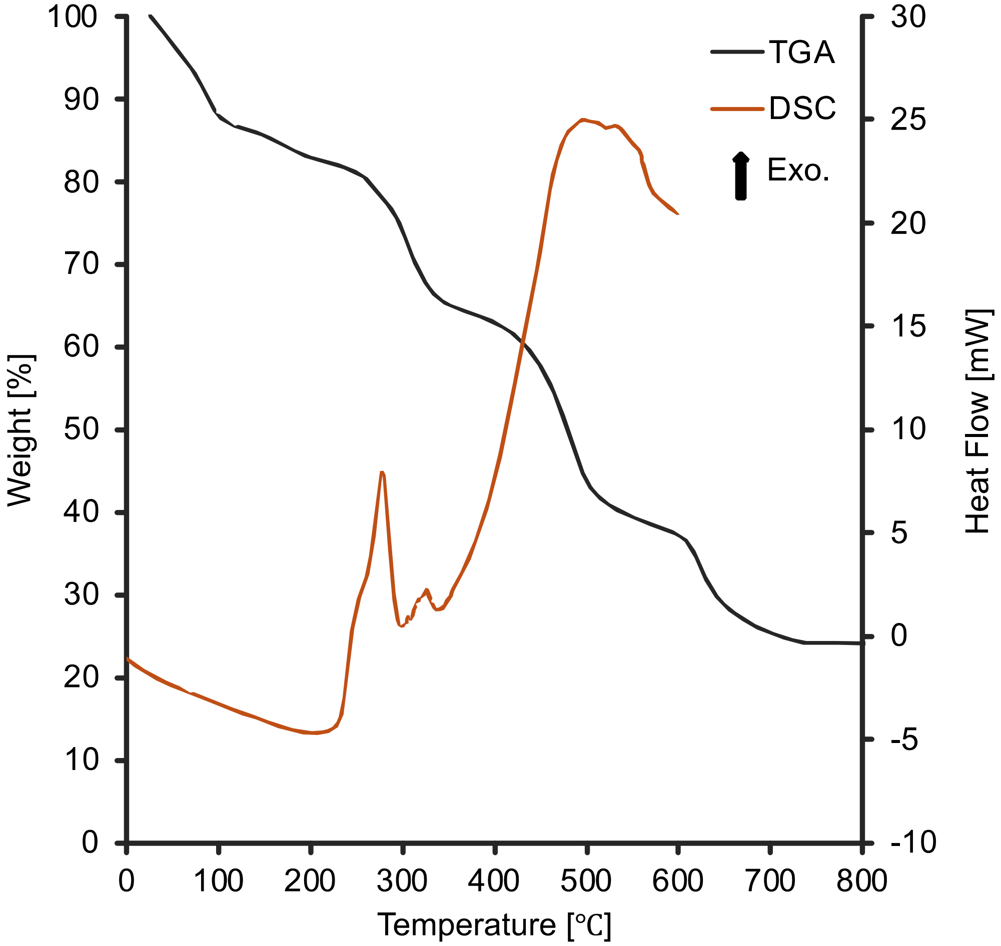

Figure S25. TGA (black) and DSC (orange) curves of supramolecule **9**, from 0 to 600 °C for DSC analysis and 800 °C for TGA.

UV-Vis spectrophotometric titrations of EOGO-*g*-[Ni-Salphen]:Pd-PTA systems

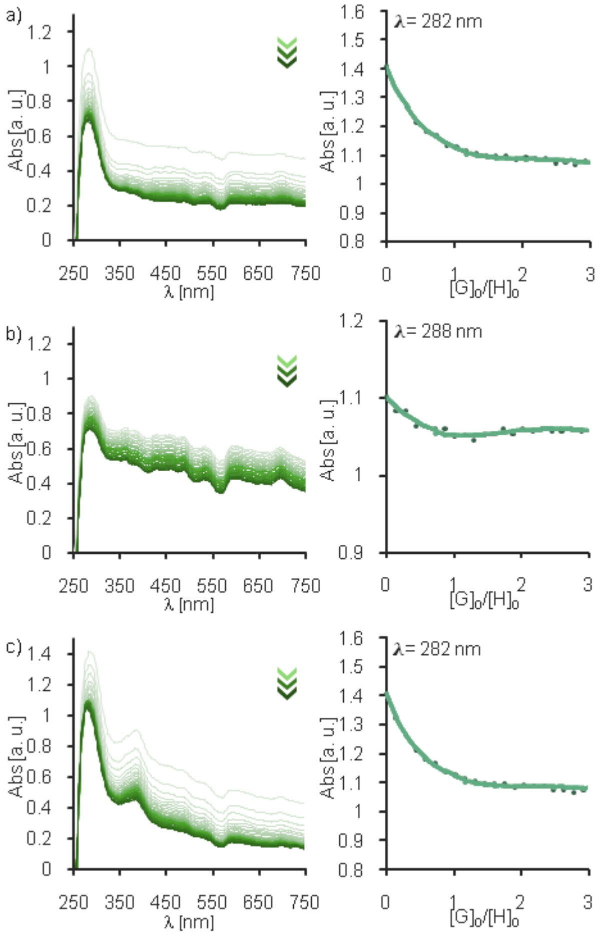

Figure S26. UV-Vis spectra from supramolecular titrations of [Ni] units in each hybrid material **4**-**6** with Pd-PTA (left column), and example of a binding isotherm obtained for each system (right column); markers represent titration data corrected from dilution and green line is from data fitting using Bindfit; the abscissas at the isotherms in the right column represent [Pd-PTA]/[Ni] ratio expressed in equivalents. (a) [Ni] in **4** vs. Pd-PTA; (b) [Ni] in **5** vs. Pd-PTA; (c) [Ni] in **6** vs. Pd-PTA.

Fluorimetric analysis of EOGO-*g*-[Ni-Salphen]:Pd-PTA systems

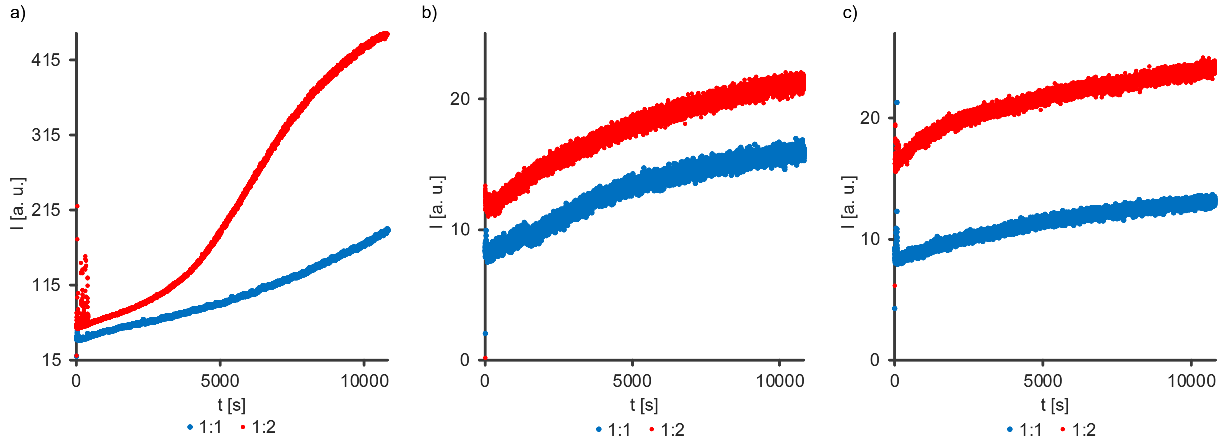

Figure S27. Kinetically monitored fluorimetry for materials **4**-**6** exposed to Pd-PTA at [Ni]:[Pd-PTA] stoichiometries 1:1 (blue) and 1:2 (red), measured at 548 nm; (a) **4**, (b) **5**, (c) **6**.

Synthesis and characterization of Supramolecular frameworks

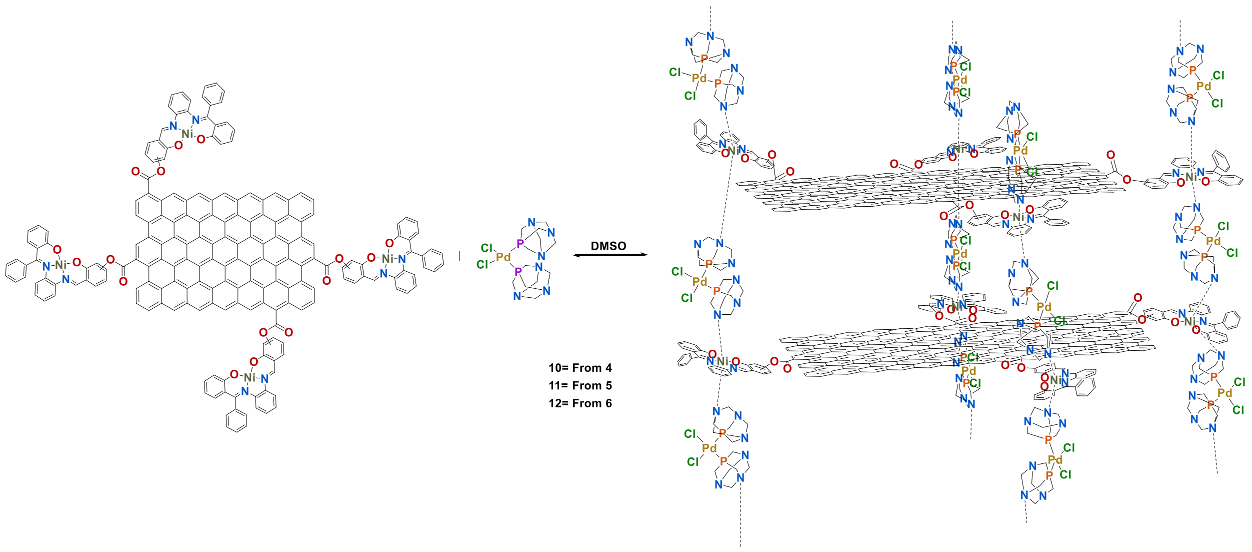

Scheme S4. Representation of the synthesis of the supramolecular frameworks.

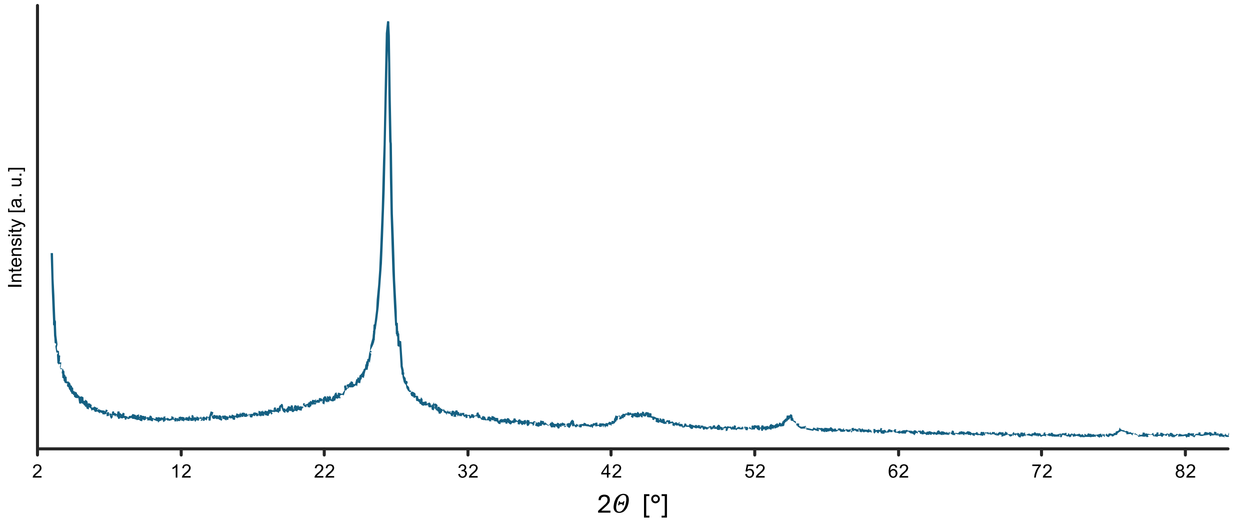

Figure S28. PXRD pattern of supramolecular framework **10**.

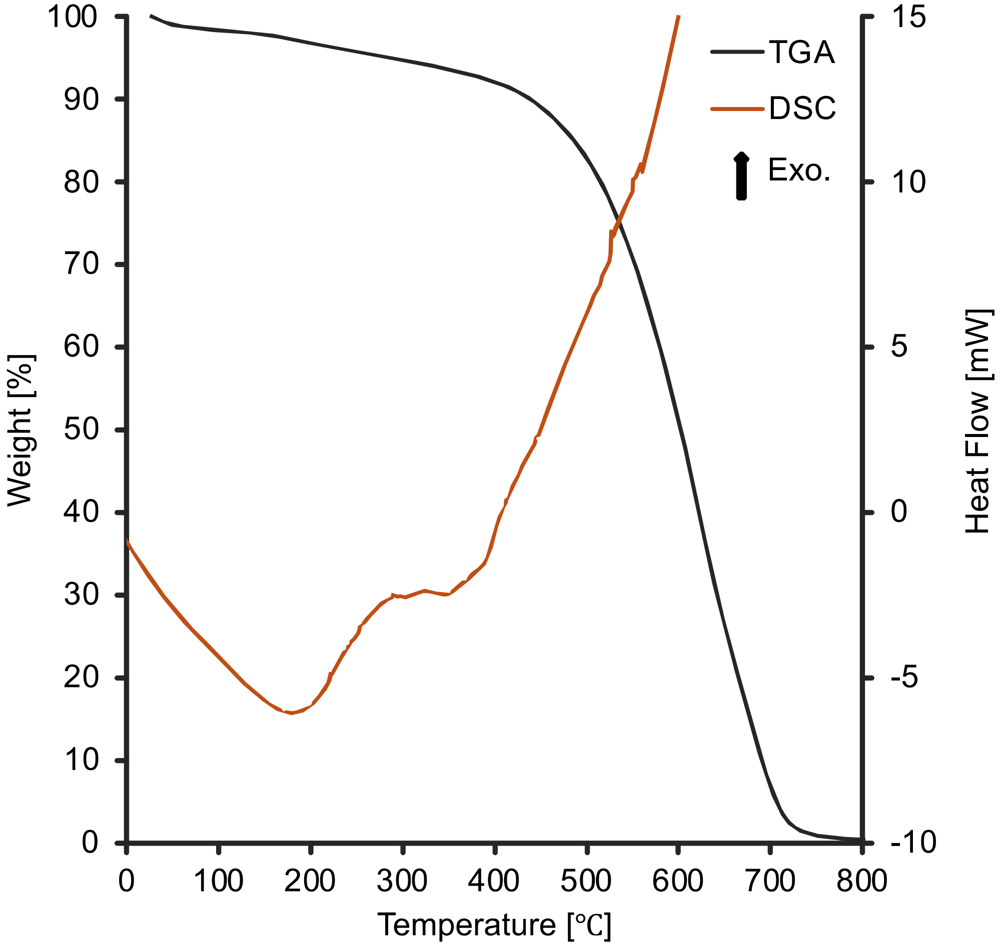

Figure S29. TGA (black) and DSC (orange) curves of supramolecular framework **10**, from 0 to 600 °C for DSC analysis and 800 °C for TGA.

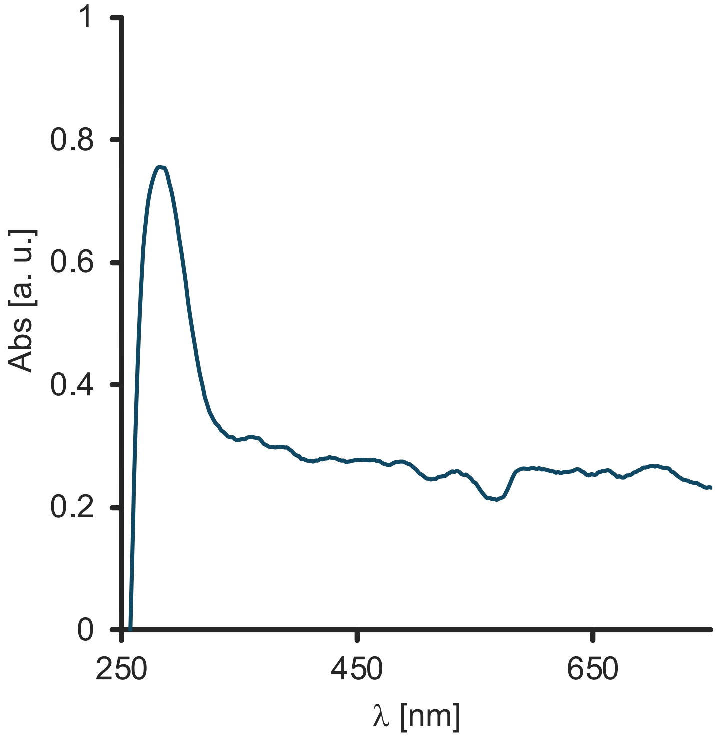

Figure S30. UV-Vis absorption spectrum of supramolecular framework **10**.

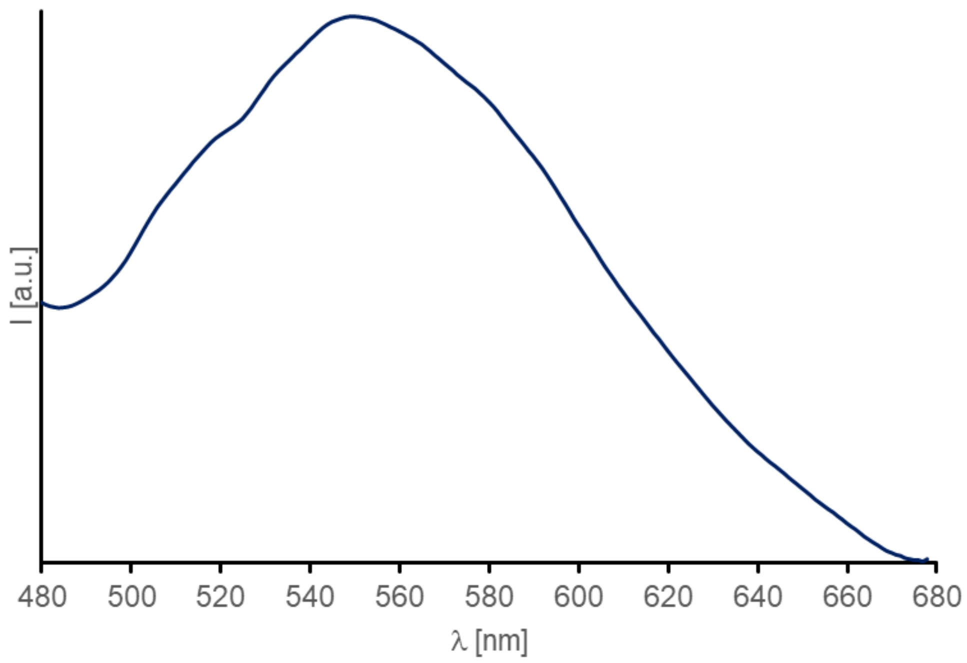

Figure S31. Fluorescence emission spectrum of supramolecular framework **10**.

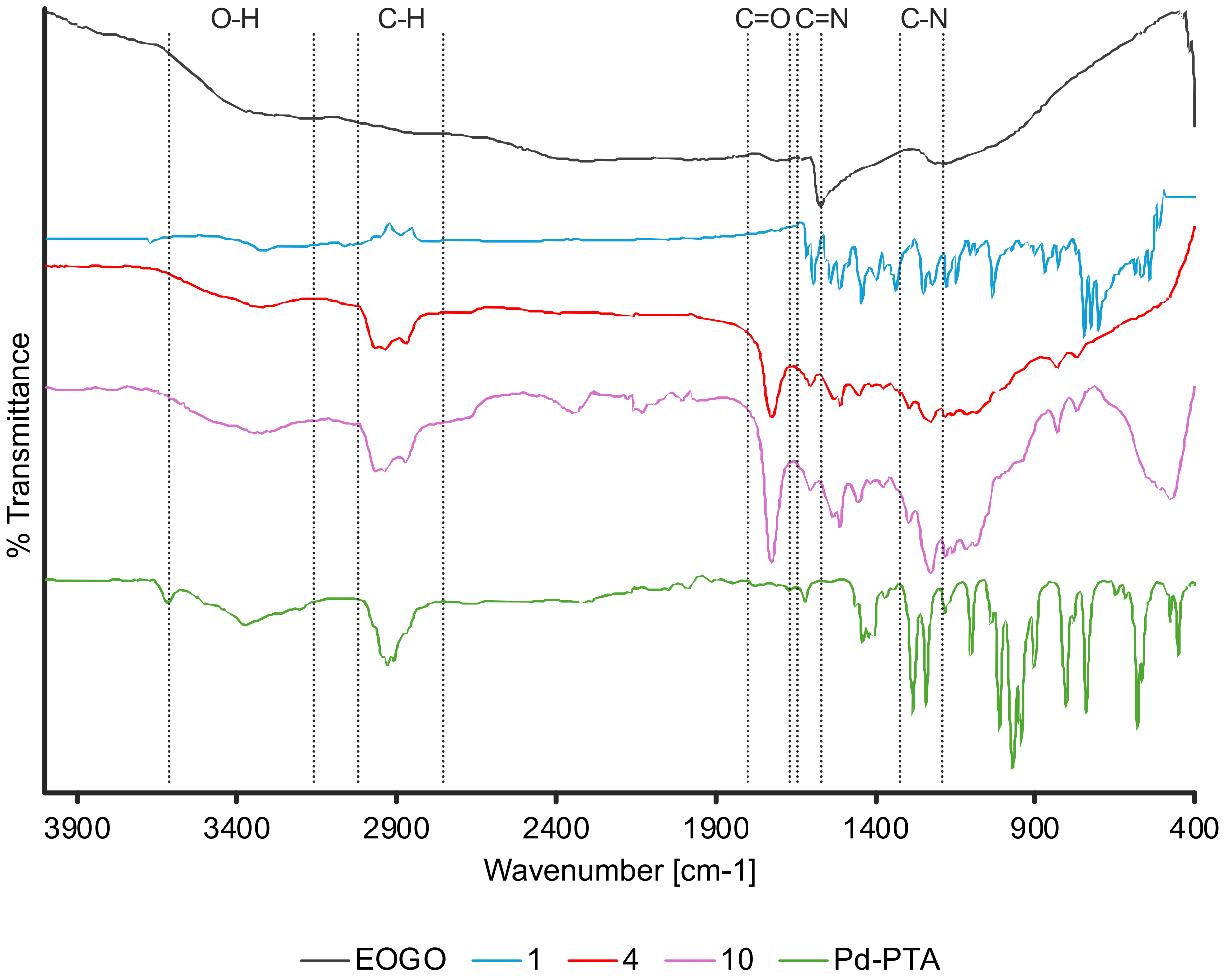

Figure S32. FTIR spectra comparison for EOGO (black), complex **1** (blue), grafted nanomaterial **4** (red); supramolecular framework **10** (purple) and Pd-PTA connector (green); regions delimited by dashed lines are for the bond types displayed accordingly above.

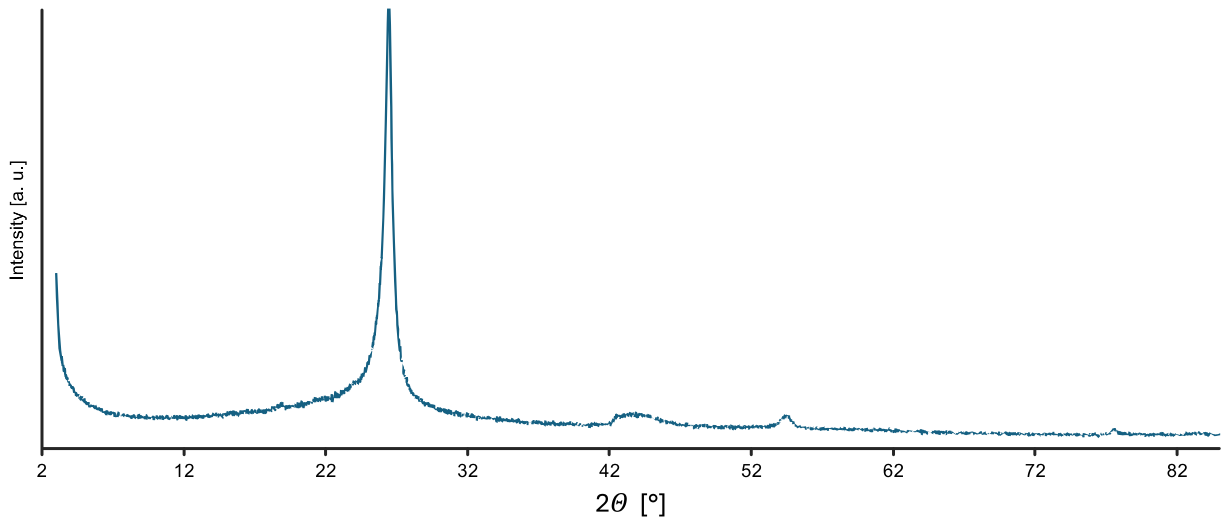

Figure S33. PXRD pattern of supramolecular framework **11**.

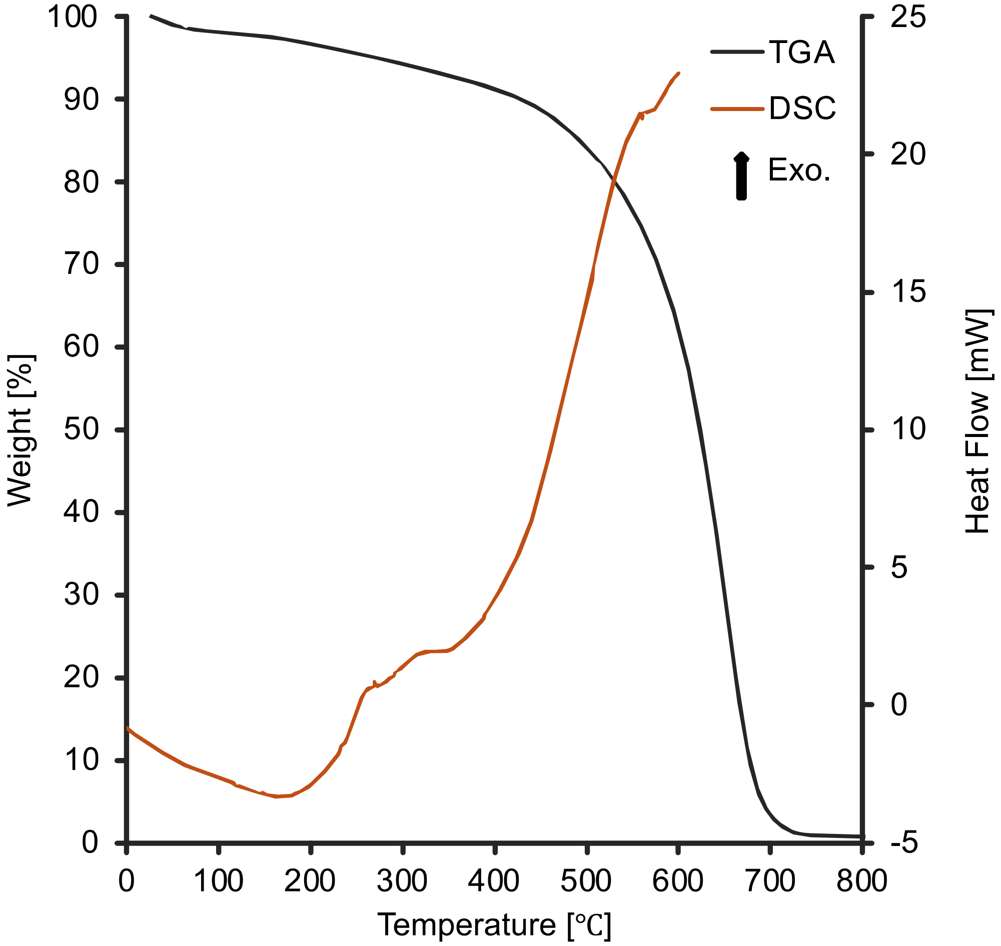

Figure S34. TGA (black) and DSC (orange) curves of supramolecular framework **11**, from 0 to 600 °C for DSC analysis and 800 °C for TGA.

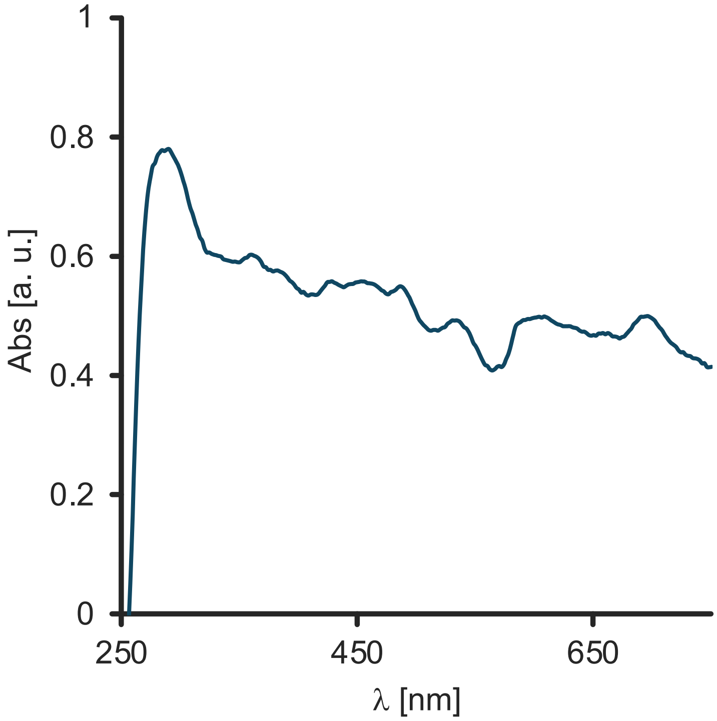

Figure S35. UV-Vis absorption spectrum of supramolecular framework **11**.

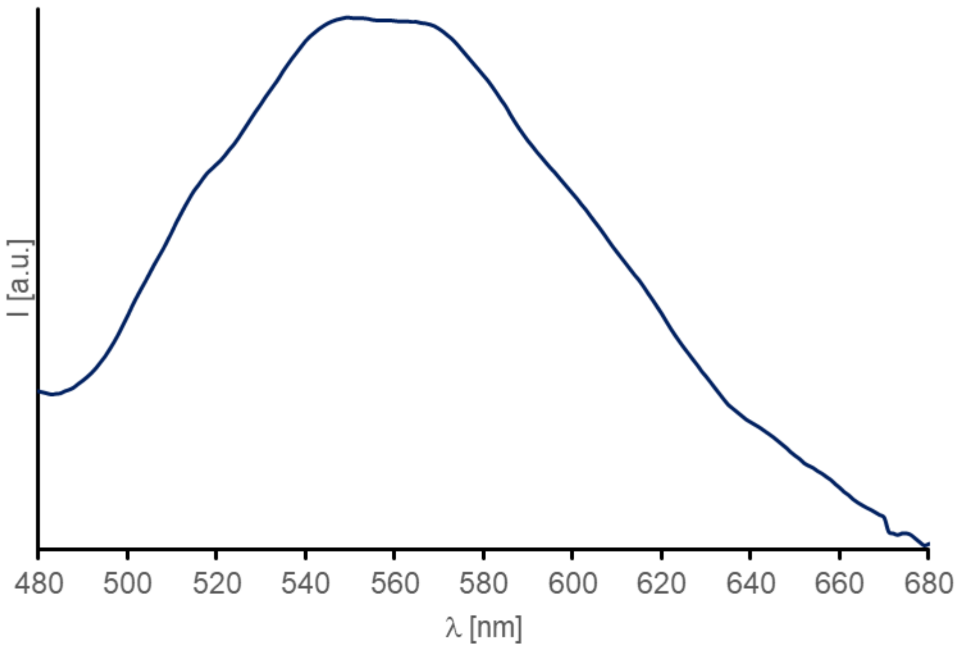

Figure S36. Fluorescence emission spectrum of supramolecular framework **11**.

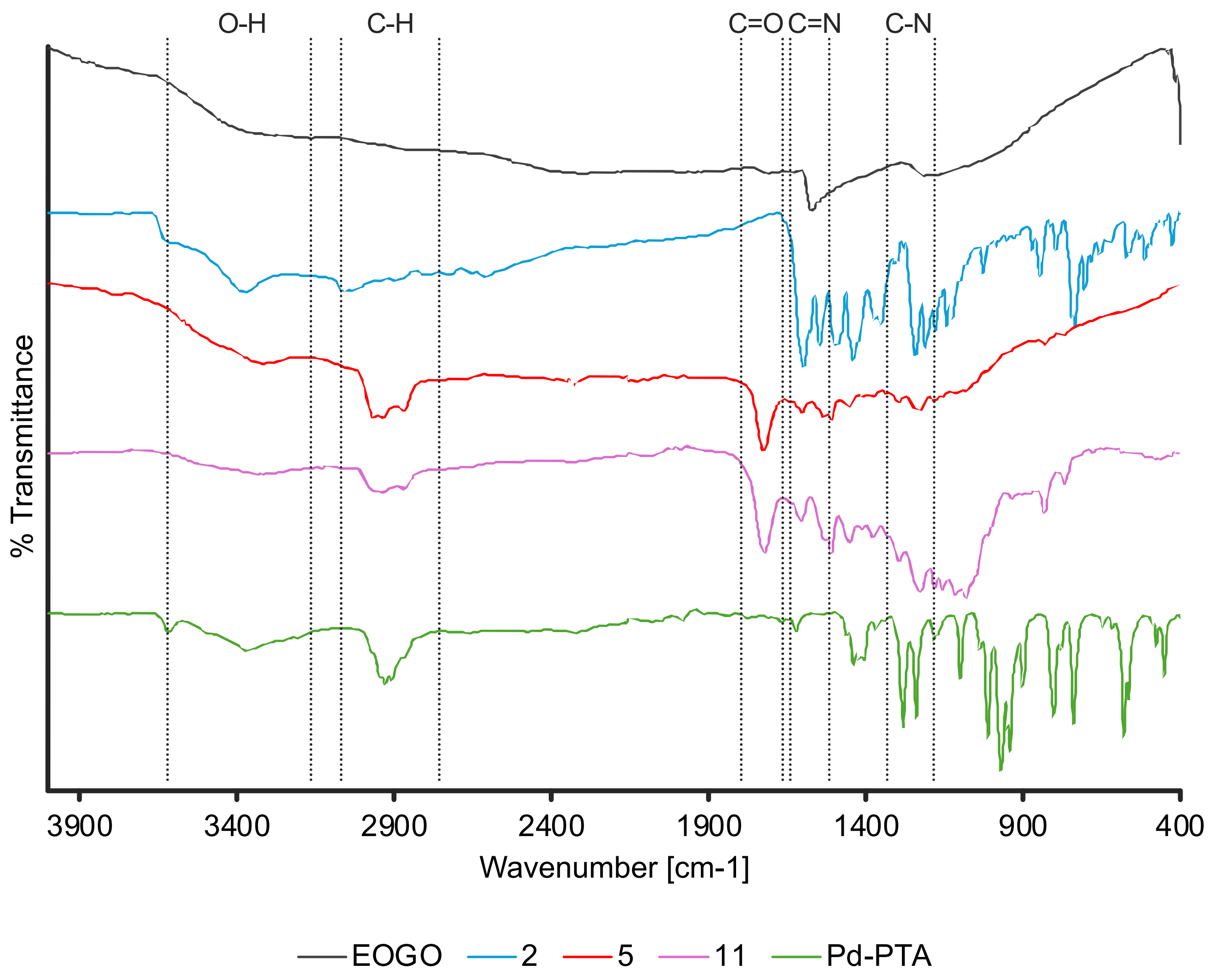

Figure S37. FTIR spectra comparison for EOGO (black), complex **2** (blue), grafted nanomaterial **5** (red), supramolecular framework **11** (purple) and Pd-PTA connector (green); regions delimited by dashed lines are for the bond types displayed accordingly above.

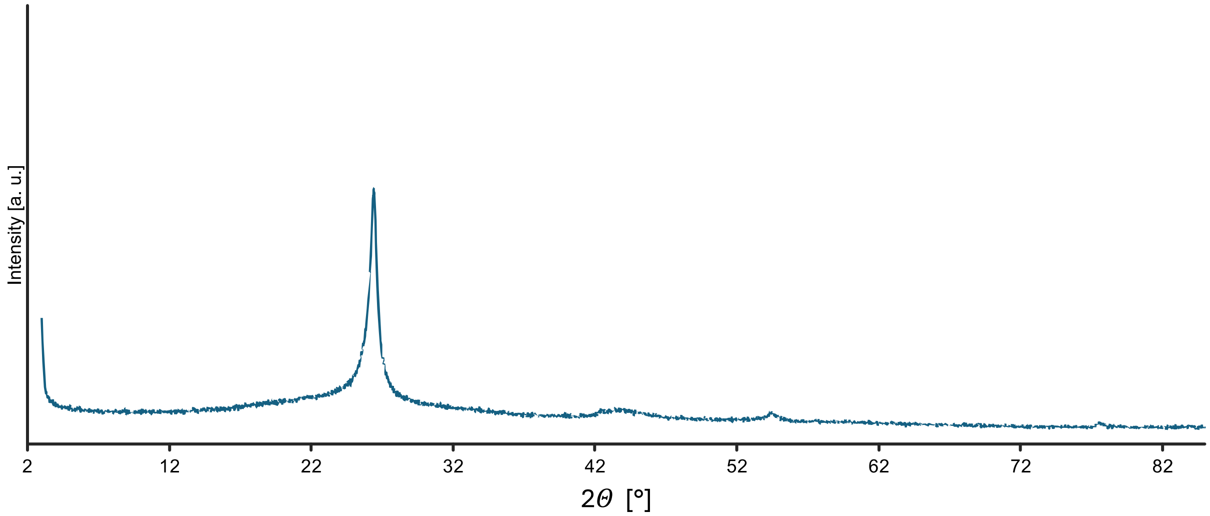

Figure S38. PXRD pattern of supramolecular framework **12**.

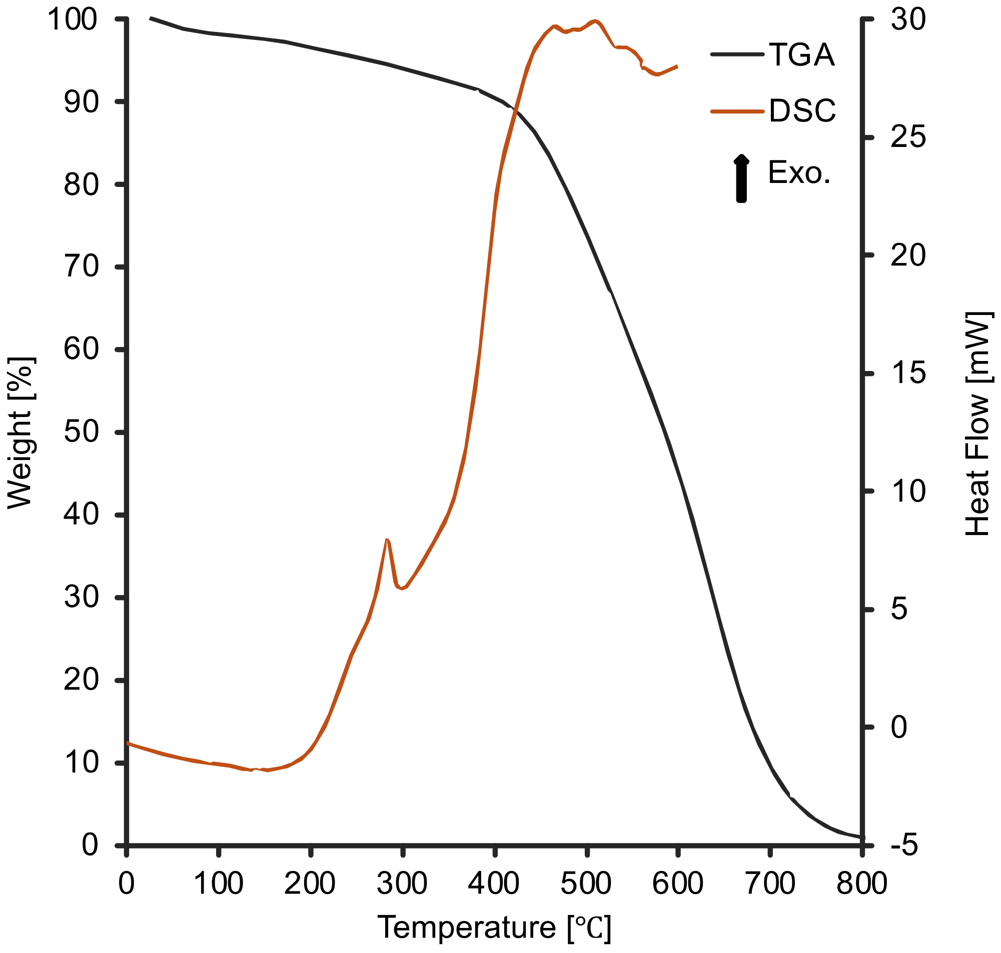

Figure S39. TGA (black) and DSC (orange) curves of supramolecular framework **12**, from 0 to 600 °C for DSC analysis and 800 °C for TGA.

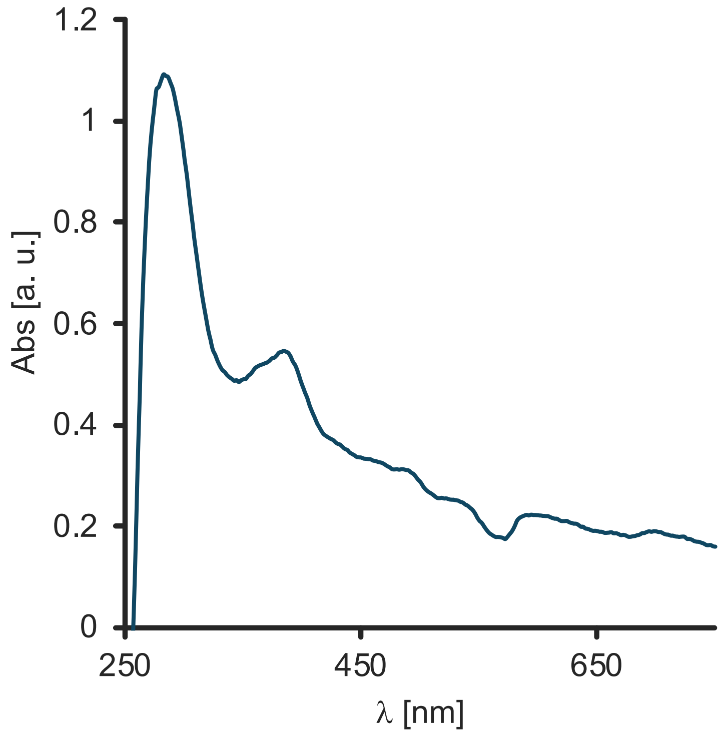

Figure S40. UV-Vis absorption spectrum of supramolecular framework **12**.

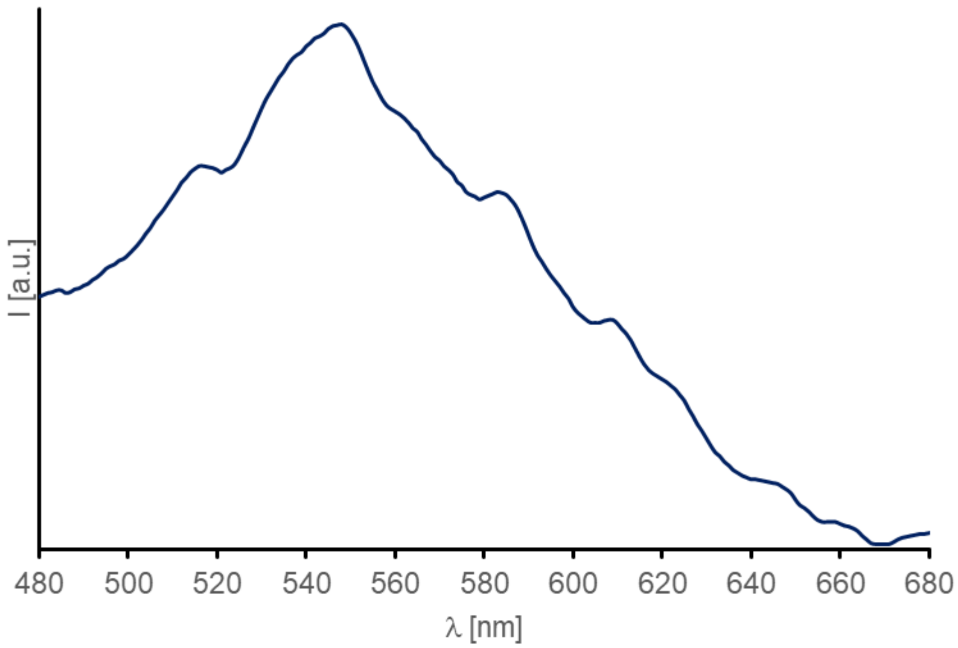

Figure S41. Fluorescence emission spectrum of supramolecular framework **12**.

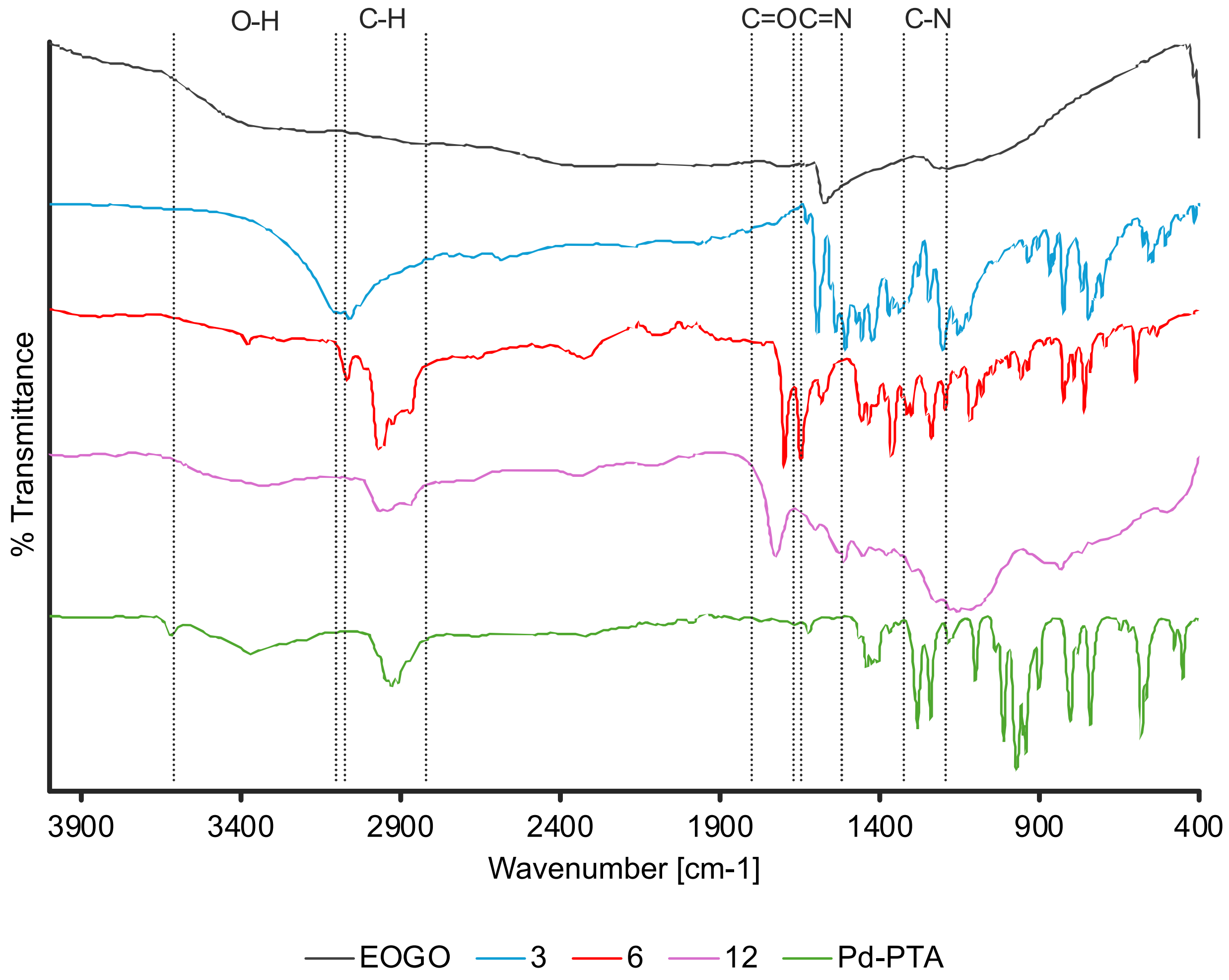

Figure S42. FTIR spectra comparison for EOGO (black), complex **3** (blue), grafted nanomaterial **6** (red), supramolecular framework **12** (purple) and Pd-PTA connector (green); regions delimited by dashed lines are for the bond types displayed accordingly above.

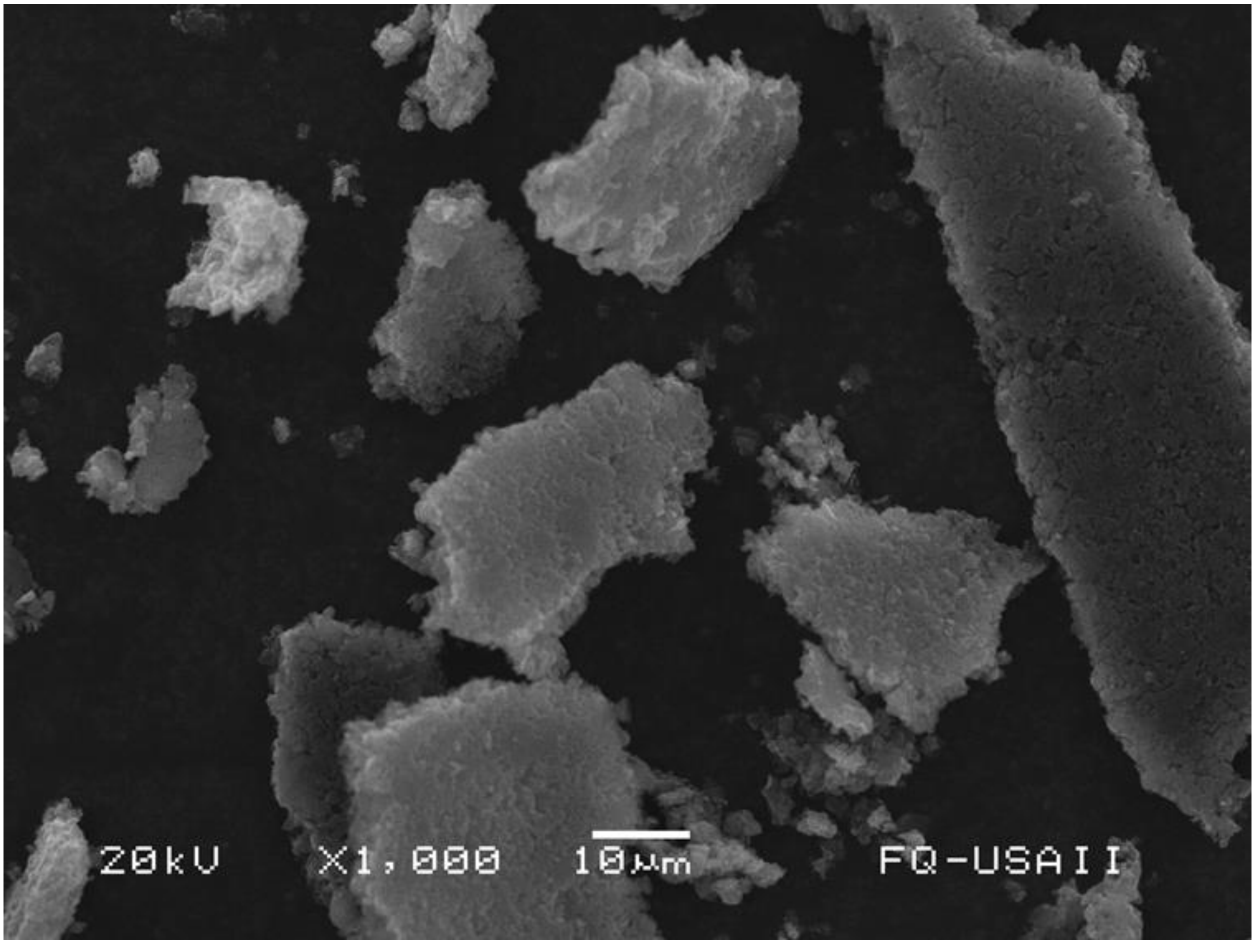

Figure S43. SEM micrograph of **12** showing the typical anisotropic assemblies obtained after the exposition of **6** to Pd-PTA.

Hill plots

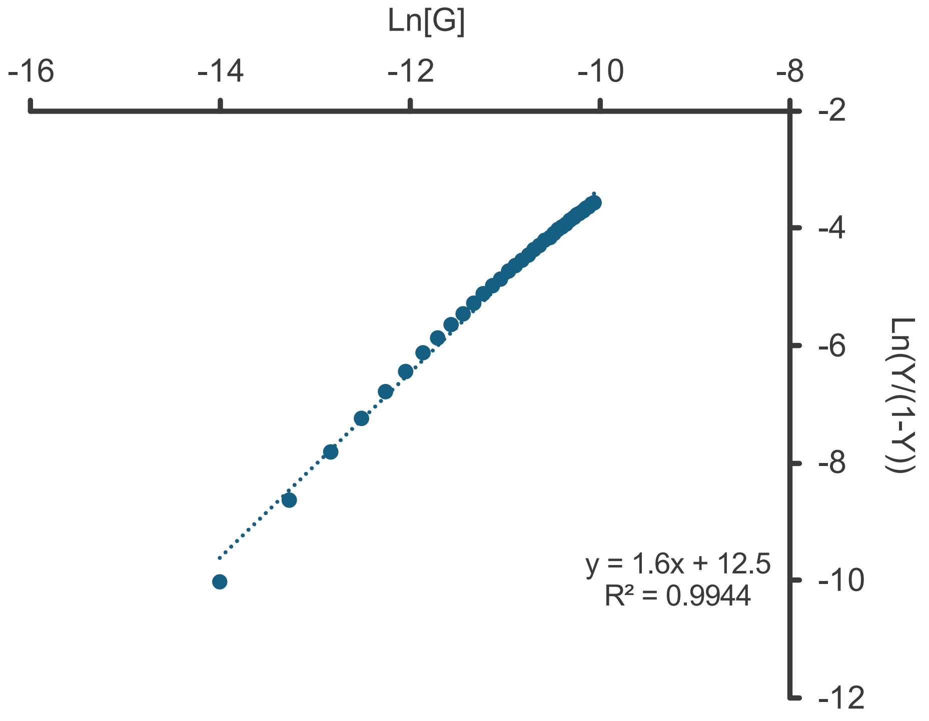

Figure S44. Hill plot for the association of Pd-PTA with **1**.

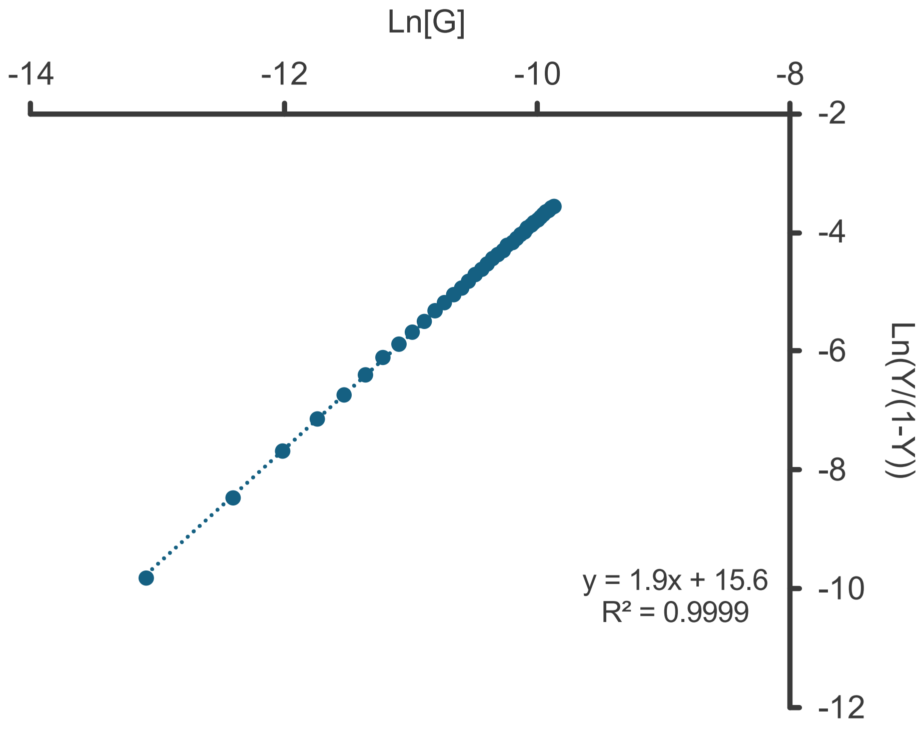

Figure S45. Hill plot for the association of Pd-PTA with **2**.

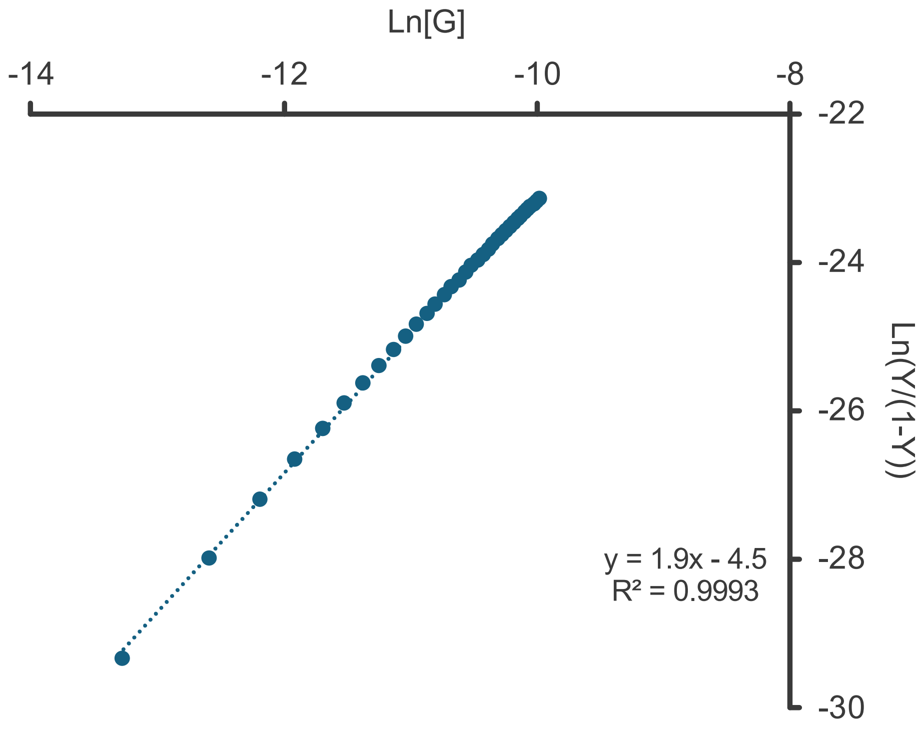

Figure S46. Hill plot for the association of Pd-PTA with **3**.

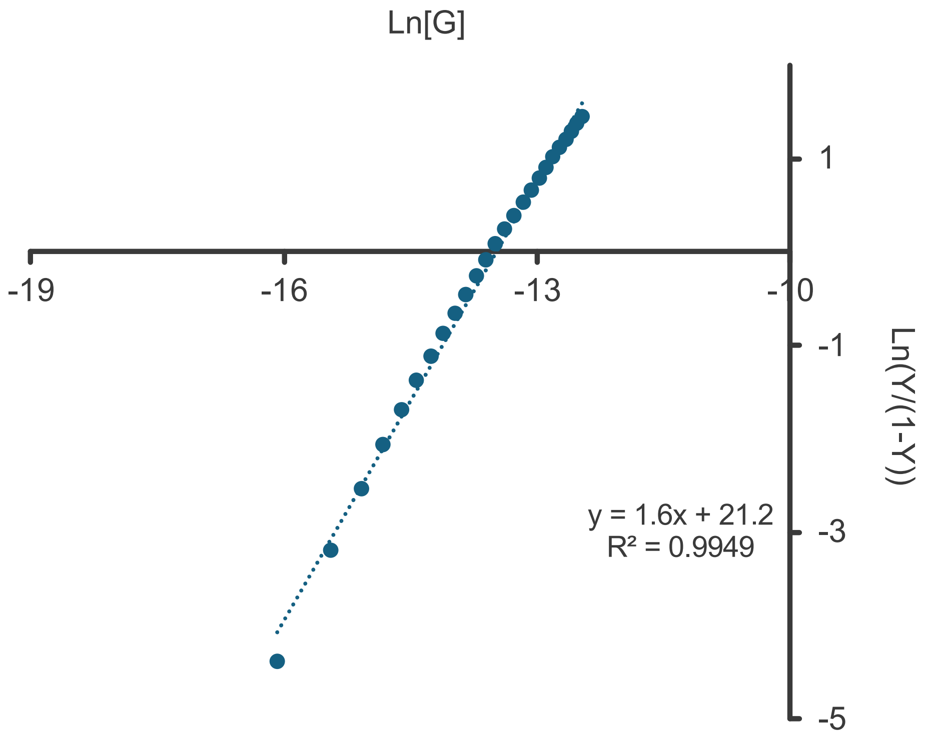

Figure S47. Hill plot for the association of [Ni] in **4** with Pd-PTA.

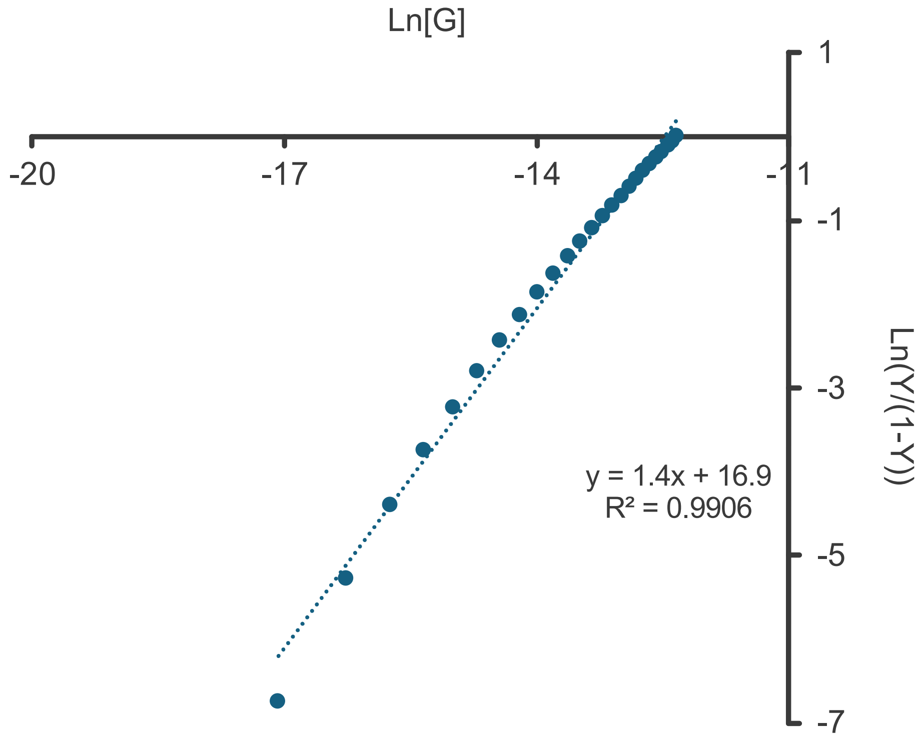

Figure S48. Hill plot for the association of [Ni] in **5** with Pd-PTA.

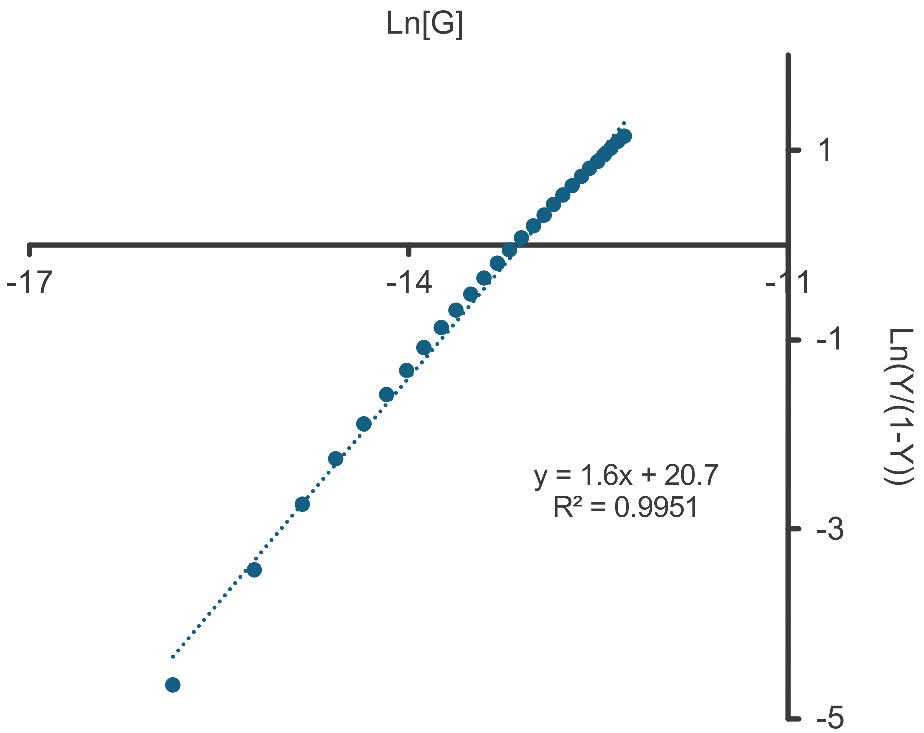

Figure S49. Hill plot for the association of [Ni] in **6** with Pd-PTA.

High resolution XPS spectra

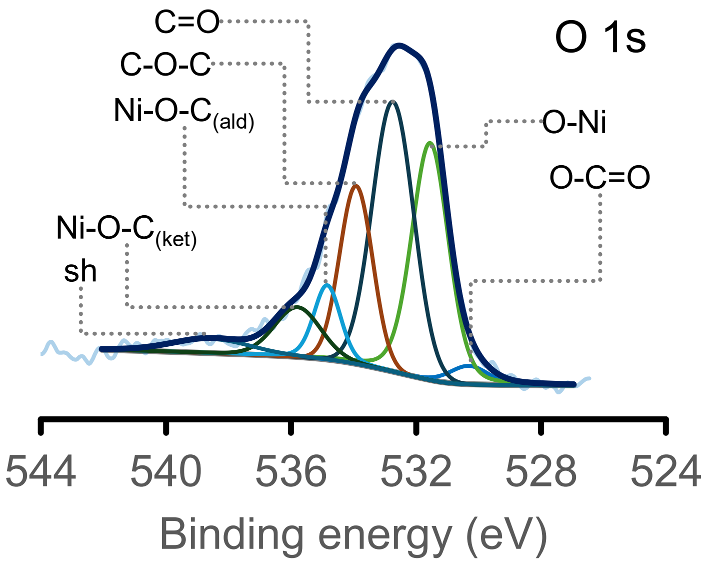

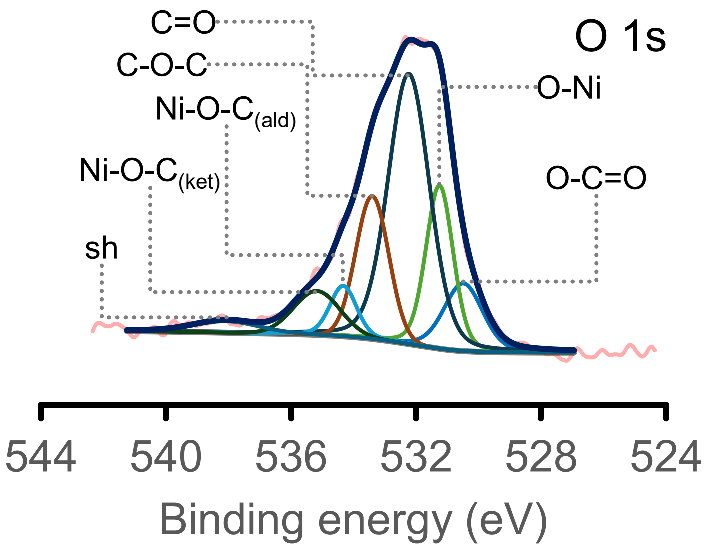

Figure S50. High-resolution XPS spectra in region O 1s for the grafted nanomaterial **6** (above) and GSF **12** (below).

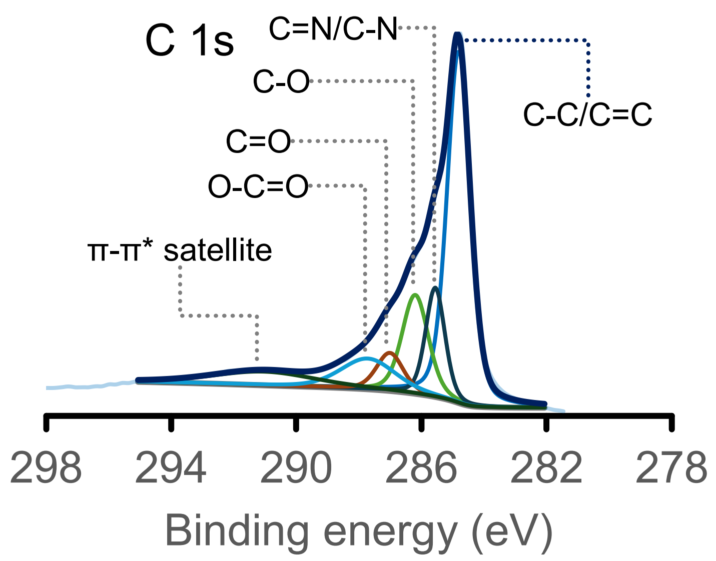

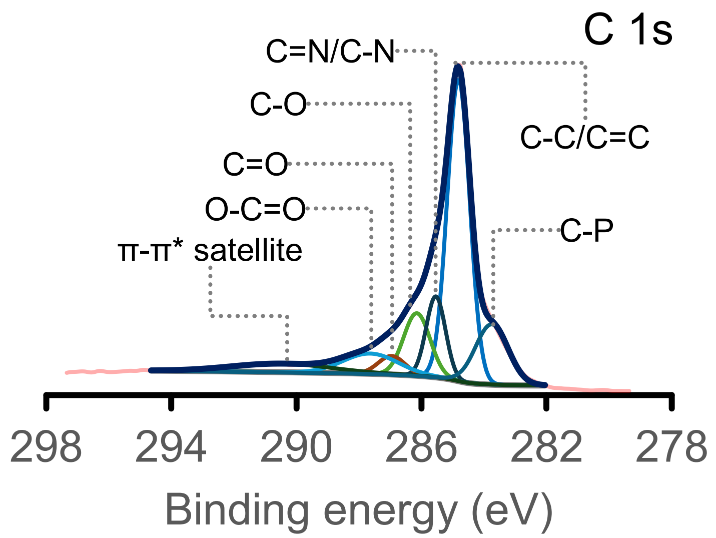

Figure S51. High-resolution XPS spectra in region C 1s for the grafted nanomaterial **6** (above) and GSF **12** (below).

Table S1. XPS analysis results from selected systems **6** and **12**.

		**6**		**12**	
Orbital	Chemical State	Binding energy (eV)	FWHM (eV)	Binding energy (eV)	FWHM (eV)
Ni $2p_{1/2}$	Ni(II) sq. pl.	873.25	2.20	872.71	1.67
	3d→4s oct.			875.28	1.96
Ni $2p_{3/2}$	Ni(II) sq. pl.	855.95	2.20	855.51	1.56
	3d→4s oct.			857.58	2.16
O 1s	sh	538.54	2.95	538.07	2.46
	O-C(N_2O_2 ket)	535.79	1.74	535.17	1.74
	O-C(N_2O_2 ald)	534.84	1.00	534.33	1.00
	C-O-C	533.91	1.24	533.4	1.24
	C=O	532.74	1.56	532.23	1.56
	O-Ni	531.55	1.39	531.25	1.03
	O-C=O	530.31	1.46	530.49	1.46
N 1s	sh	405.08	0.93	404.42	4.11
	C-N* π-π*	401.90	3.34	402.38	1.04
	N=C(ket)	400.98	1.80	401.19	1.21
	N=CH(ald)	399.97	1.46	400.16	1.23
	N-CH (amine)			399.33	1.31
	Ni-N-CH (amine)			397.89	1.04
Pd $3d_{3/2}$	Pd(II) (Pd-P/Pd-Cl)			343.77	1.57
Pd $3d_{5/2}$	Pd(II) (Pd-P/Pd-Cl)			338.50	1.38

Table S1. XPS analysis results from selected systems **6** and **12** (continues).

C 1s	π-π* satellite	291.04	3.85	290.50	3.74
	O-C=O	287.72	2.05	287.64	2.05
	C=O	287.01	1.00	286.96	1.00
	C-O	286.20	0.99	286.15	0.99
	C=N/C-N	285.56	0.73	285.55	0.73
	C-C/C=C	284.81	0.87	284.82	0.84
	C-P			283.76	1.24
$P\ 2p_{3/2}$	P-C			132.49	2.30
	P-Pd			130.08	2.54
